# Development of a Metastasis-Related Immune Prognostic Model of Metastatic Colorectal Cancer and Its Usefulness to Immunotherapy

**DOI:** 10.3389/fcell.2020.577125

**Published:** 2021-01-28

**Authors:** Zhiwen Luo, Xiao Chen, Yefan Zhang, Zhen Huang, Hong Zhao, Jianjun Zhao, Zhiyu Li, Jianguo Zhou, Jianmei Liu, Jianqiang Cai, Xinyu Bi

**Affiliations:** Department of Hepatobiliary Surgery, National Cancer Center/National Clinical Research Center for Cancer/Cancer Hospital, Chinese Academy of Medical Sciences and Peking Union Medical College, Beijing, China

**Keywords:** immune prognostic model, immunotherapy, disease recurrence, metastatic colorectal cancer, bioinformatics, real-world cohort

## Abstract

**Background:** Post-surgical recurrence of the metastatic colorectal cancer (mCRC) remains a challenge, even with adjuvant therapy. Moreover, patients show variable outcomes. Here, we set to identify gene models based on the perspectives of intrinsic cell activities and extrinsic immune microenvironment to predict the recurrence of mCRC and guide the adjuvant therapy.

**Methods:** An RNA-based gene expression analysis of CRC samples (total = 998, including mCRCs = 344, non-mCRCs = 654) was performed. A metastasis-evaluation model (MEM) for mCRCs was developed using the Cox survival model based on the prognostic differentially expressed genes between mCRCs and non-mCRCs. This model separated the mCRC samples into high- and low-recurrence risk clusters that were tested using machine learning to predict recurrence. Further, an immune prognostic model (IPM) was built using the COX survival model with the prognostic differentially expressed immune-related genes between the two MEM risk clusters. The ability of MEM and IPM to predict prognosis was analyzed and validated. Moreover, the IPM was utilized to evaluate its relationship with the immune microenvironment and response to immuno-/chemotherapy. Finally, the dysregulation cause of IPM three genes was analyzed in bioinformatics.

**Results:** A high post-operative recurrence risk was observed owing to the downregulation of the immune response, which was influenced by MEM genes (*BAMBI, F13A1, LCN2*) and their related IPM genes (*SLIT2, CDKN2A, CLU*). The MEM and IPM were developed and validated through mCRC samples to differentiate between low- and high-recurrence risk in a real-world cohort. The functional enrichment analysis suggested pathways related to immune response and immune system diseases as the major functional pathways related to the IPM genes. The IPM high-risk group (IPM-high) showed higher fractions of regulatory T cells (Tregs) and smaller fractions of resting memory CD4+ T cells than the IPM-low group. Moreover, the stroma and immune cells in the IPM-high samples were scant. Further, the IPM-high group showed downregulation of MHC class II molecules. Additionally, the Tumor Immune Dysfunction and Exclusion (TIDE) algorithm and GDSC analysis suggested the IPM-low as a promising responder to anti-CTLA-4 therapy and the common FDA-targeted drugs, while the IPM-high was non-responsive to these treatments. However, treatment using anti-CDKN2A agents, along with the activation of major histocompatibility complex (MHC) class-II response might sensitize this refractory mCRC subgroup. The dysfunction of *MEIS1* might be the reason for the dysregulation of IPM genes.

**Conclusions:** The IPM could identify subgroups of mCRC with a distinct risk of recurrence and stratify the patients sensitive to immuno-/chemotherapy. Further, for the first time, our study highlights the importance of MHC class-II molecules in the treatment of mCRCs using immunotherapy.

## Introduction

Colorectal cancer is among the most commonly diagnosed cancers and a leading cause of cancer-related deaths globally. Further, during the development of CRC, 40–50% cases show metastasis (mCRC) (Reissfelder et al., 2009), especially to the liver, which accounts for the highest morbidity and mortality in colorectal cancer (Amano et al., 2014; Leung et al., 2016).

Resection of metastatic lesions is considered the only curative treatment for mCRC and increases the 5-year survival rate to 30–50% (Fong, 1999; Reissfelder et al., 2009), although only in selected cases. Despite the advances in treatment modalities, such as neoadjuvant or adjuvant chemotherapy (Sadot et al., 2015; Kim et al., 2019), the recurrence rate of mCRC within 2 years is almost 50% (Ryuk et al., 2014; Xiong et al., 2018). Thus, the recurrence of mCRC is heterogeneous. Therefore, several criteria, such as the Fong's clinical risk score (CRS), have been constructed to select mCRC cases with a better prognosis after surgery. These criteria, which are mainly based on radiological and clinicopathological parameters, such as size, number of tumors, and response to neoadjuvant chemotherapy, can predict prognosis after resection (Fong, 1999; Wang et al., 2017). However, the prognostic factors landscape for predicting the outcome of mCRC is changing (Spolverato et al., 2013). During the past few decades, biological, and genomic alterations have been studied in cancer cells to identify subgroups with specific prognoses and distinct treatment responses and to find potential drug targets (Volinia and Croce, 2013; Xiong et al., 2018). Further, numerous prognosis-predicting models are now relying on combining the clinicopathological factors with tumor-specific molecular markers to aid in the clinical decision-making process by the cumulative assessment of multiple tumor factors within a single scoring system. Although it is known that the understanding that malignant phenotype of cancer cells is determined by their intrinsic activities, surroundings, and the recruitment and activation of immune cells in the tumor-related microenvironment has increased (Ben-Baruch, 2003; Zhang et al., 2016; Xiong et al., 2018), existing prediction models consider the role of intrinsic factors only. Thus, it is unclear whether these models would comprehensively represent the malignancy of mCRC from the perspective of extrinsic factors. Moreover, most of the existing models have been unable to explain the biology of cancers accurately and have failed to be translated into useful therapeutic approaches.

Further, immune diseases could promote the development and progression of cancer. The cancer cells can stimulate a specific immune response, thus enriching an appropriate microenvironment for their growth (Long et al., 2019). Moreover, the host's immune status can alter the function and composition of the tumor-infiltrating cells (TIC) and determine the clinical outcome. For instance, it has reported that immune-related TIC could predict the overall survival of cancer (Long et al., 2019), and the immune microenvironment could determine the clinical outcome in CRC patients (Xiong et al., 2018; Ye et al., 2019). However, few studies have systematically focused on mCRC, this progressive CRC subtype, so the relationship of its immune phenotype with its recurrence after surgery is still unclear. Here, we hypothesized that some recurrence-related genes in mCRC might interact with immune-related genes, which could elicit a significant immune response and provide an adequate microenvironment for the development and progression of mCRC. Further, such a microenvironment could change the response to adjuvant therapy, to prompt the post-operative recurrence of mCRC. Therefore, there might be a gene signature to stratify the specific malignant phenotypes, representing the altered intrinsic activities of the tumor cells and the tumor-related microenvironment comprehensively, thus predicting the recurrence risk of mCRC.

Therefore, we set to identify a gene model to elucidate and predict recurrence in mCRC patients.

## Materials and Methods

### Data Acquisition

The data in this manuscript was composed of two parts from Gene Expression Omnibus (GEO) and The Cancer Genome Atlas (TCGA) and with 942 cases in GEO as reported in *Acquisition of the Microarray Data* and 56 cases in TCGA as reported in *Acquisition of the RNA-Sequencing Data*.

### Acquisition of the Microarray Data

The gene expression profile matrix files from GSE72968 and GSE72969 based on GPL570 (22 M0 and 102 M1 samples), GSE39582 based on GPL570-55999 (376 M0 and 54 M1 samples), GSE41258 based on GPL96 (125 M0 and 88 M1 samples), GSE81558 based on GPL15207 (5 M0 and 18 M1 samples), and GSE71222 based on GPL570 platform (126 M0 and 26 M1 samples) were downloaded from the GEO database (https://www.ncbi.nlm.nih.gov/geo/) to analyze the different colorectal cancer samples. The entire gene expression data were log2 transformed, and average RNA expression values were considered in case of duplicate data. Next, only genes with an average expression value >1 was retained, while the low-abundance RNA reads were discarded. Because The M1 colorectal cancer samples from four datasets, *viz*. GSE72968 and GSE72969 (*n* = 102) and GSE39582 (*n* = 54) and GSE41258 (*n* = 88), included survival information, GSE72968 and GSE72969 (*n* = 102) were integrated into the training cohort, while GSE39582 (*n* = 54) and GSE41258 (*n* = 88) were integrated into the MEM validation cohort. The sva package (version: 3.30.1; http://bioconductor.org/packages/release/bioc/html/sva.html) was used to eliminate batch effects, and the scale method of the limma R package (Version 3.38.3; http://www.bioconductor.org/packages/release/bioc/html/limma.html) helped in normalizing the data.

### Acquisition of the RNA-Sequencing Data

Gene expression data and the corresponding clinical datasheets for 56 mCRC samples were obtained from The Cancer Genome Atlas (TCGA) website (https://portal.gdc.cancer.gov/repository) (up to May 1, 2019) as the TCGA mCRC cohort. The sequencing data were obtained using the Illumina HiSeq_RNA-Seq and Illumina HiSeq_miRNA-Seq platforms. The analysis reported herein completely satisfies the TCGA publication requirements (http://cancergenome.nih.gov/publications/publicationguidelines). The gene symbols were annotated based on the Homo_sapiens.GRCh38.91.chr.gtf file (http://asia.ensembl.org/index.html). Log2 transformation was performed for all gene expression data. The function of the trimmed mean of M values (TMM) normalization method of the edgeR package (Version 3.24.3; http://www.bioconductor.org/packages/release/bioc/html/edgeR.html/) of the R software (Version 3.5.2; https://www.r-project.org/) was applied to normalize the data.

### Patients in the CICAMS CRLM Cohort and Sample Collection

From January to August 2016, a total of 60 frozen, surgically resected tumor tissues were obtained from patients with pathological diagnosis of colorectal cancer liver metastasis at the National Cancer Center/National Clinical Research Center for Cancer/Cancer Hospital, Chinese Academy of Medical Sciences and Peking Union Medical College. Total RNA was extracted from these frozen samples using TRIzol reagent (Thermo, #15596-018) according to the standard protocols. Then, total RNA samples were reverse transcribed to single-stranded complementary DNA (cDNA) using a Prime Script RT reagent kit (Promega, # A5001). The cDNA samples were prepared for quantitative real-time polymerase chain reaction (qRT-PCR). This project was approved by the Institutional Review Boards of the National Cancer Center/National Clinical Research Center for Cancer/Cancer Hospital, Chinese Academy of Medical Sciences and Peking Union Medical College, and the requirement for informed consent was waived due to the study's retrospective nature. qRT-PCR was used to detect the expression of the IPM genes in frozen tissue samples from patients with mCRC. qRT-PCR was performed using Bestar qPCR MasterMix (DBI Bioscience, #DBI-2043) and was assessed by Agilent Mx3000. The relative abundance of mRNA for each of the three genes was normalized to glyceraldehyde 3-phosphate dehydrogenase (GAPDH) and z-score transformed. The primer sequences used to amplify the three genes are shown below.

**Table:** 

**Gene primer**	**Sequences**
GAPDH-F	TCAAGAAGGTGGTGAAGCAGG
GAPDH-R	GCGTCAAAGGTGGAGGAGTG
CDKN2A1-F	GGTTTTCGTGGTTCACATCCC
CDKN2A1-R	AGACGCTGGCTCCTCAGTA
SLIT2-F	CTGGGGAGCGGGTAGATAGG
SLIT2-R	ATCGCAAGGTGACTCCGTTT
CLU-F	TGCACGTCACCAAGTAACCA
CLU-R	GAGCAGCAGAGTCGAGTGTT

### Analysis of Differentially Expressed Genes to Identify Genes Involved in Metastasis

We comprehensively compared the M1 and M0 colorectal cancer samples to identify differentially expressed genes (DEGs) using the robust rank aggregation (RobustRankAggreg) R package, and the thresholds were set as |log2-fold change (FC)| > 1.0 and false discovery rate (FDR) <0.05.

### Development and Validation of MEM

The expression profiles of the DEG obtained from the training cohort were analyzed to build a MEM using the following methods. Univariate, least absolute shrinkage and selection operator (LASSO), and multivariate Cox regression analyzes were employed to investigate the correlation between progression-free survival (PFS) of patients and the expression levels of each DEG. The expression of genes was considered statistically significant when the *P* < 0.05 in the univariate Cox regression analysis. For highly correlated genes, the traditional Cox regression model could not be used directly; thus, LASSO with L1-penalty, a popular method for determining interpretable prediction rules that handle the collinearity problem, was used. For the LASSO-penalized Cox regression selection operator, we subsampled the dataset with 1,000 times replacement and selected the markers with repeat occurrence frequencies above 900. The tuning parameters were determined based on the expected generalization error estimated from 10-fold cross-validation and information-based criteria, Akaike Information Criterion/Bayesian Information Criterion (AIC/BIC), and the largest value of lambda was adopted such that the error was within one standard error of the minimum, called “1 – se” lambda. Finally, a multivariate Cox regression analysis was conducted to assess the contribution of a gene as an independent metastasis factor correlated with PFS. A stepwise method was employed to further select the best model. A three-gene-based metastasis risk score was established based on a linear combination of the regression coefficient derived from the multivariate Cox regression model (β), multiplied with its expression level. The Metastatic Index (MI) was calculated as, MI = (β1^*^expression level of *BAMBI*) + (β2^*^ expression level of *F13A1*) + (β3^*^expression level of *LCN2*). The optimal cutoff value was determined using the X-tile 3.6.1 software (Yale University, New Haven, CT, USA). The thresholds for the scores obtained from the MEM applied to classify patients into low- and high-recurrence risk clusters were defined as the scores that yielded the largest χ^2^-value in the Mantel–Cox test. The training patients with survival data were separated into low- and high-recurrence risk clusters based on the optimal cutoff value. The Kaplan–Meier (K–M) survival curves for cases with low or high metastasis risk were generated. The time-dependent receiver operating characteristic (ROC) curve analyses were performed to evaluate the predictive power, and the validation cohort was used to confirm the outcome of MEM. Five representative supervised machine learning (ML) algorithms, including decision tree (DT), random forest (RF), supporting vector machine (SVM), neural network (NN), and a conventional logistic regression (LR) algorithm were used to test the potential of MEM to predict the recurrence of mCRC.

Based on the MEM criteria, gene set enrichment analysis (GSEA) was used to determine whether immune pathways in different mCRC clusters differ from each other according to corresponding immune-related genes. The additional details have been provided in Supplementary Materials.

### Development and Validation of MEM-Related IPM

DEG analysis was used to find the differentially expressed immune genes. In order to investigate the function of the MEM-related immune genes, we constructed an IPM to reveal the significance of the MEM in predicting the recurrence of mCRC. The prognostic value of differentially expressed immune genes for predicting PFS was defined using univariate Cox regression analysis, where *P* < 0.05 was considered a significant association. Next, LASSO was used to identify the key immune prognostic genes. Finally, an IPM was constructed utilizing the regression coefficients derived from the multivariate Cox regression analysis to multiply the expression level of each immune gene. The X-tile 3.6.1 software was recruited to determine the optimal cutoff for mCRC patients classified into low- and high-recurrence risk groups. The log-rank test and K–M survival analyzes were used to assess the predictive ability of MEM-related IPM, which were validated in the TCGA mCRC cohort.

### GO Terms Semantic Analysis to Identify Hub Gene

We used a GOSemSim R package (http://www.bioconductor.org/packages/release/bioc/html/GOSemSim.html) to perform a semantic similarity measure and predict the function, position, interaction, and correlation of the hub gene from the MEM and IPM genes identified in our analysis.

### Estimation of the Immune Environment

Cell-Type Identification by Estimating Relative Subsets of Known RNA Transcripts (CIBERSORT) deconvolution analysis was performed to estimate and elucidate the fractions of 22 human hematopoietic cell phenotypes in IPM subtypes. Further, the Estimation of Stromal and Immune cells in Malignant Tumor tissues Using Expression Data (ESTIMATE) algorithm was used to quantitate the infiltration of stroma and immune cells in IPM low- and high-risk groups.

### Expression of HLA Subtype Genes Between the IPM Groups

Before recognition by T cells, the tumor antigen must be processed and combined with major histocompatibility complex (MHC) class I molecules. Thus, the expression of the human leukocyte antigen (HLA) can affect T-cell recognition of tumor antigen (Rooney et al., 2015) and influence the local immune status. Therefore, we analyzed the expression of all HLA subtype genes between low- and high-risk IPM groups using the Wilcoxon test.

### Immuno- and Chemotherapeutic Response

The TIDE algorithm and subclass mapping analysis were utilized to predict the response of IPM risk groups to immune checkpoint blockades, as described previously (Hoshida et al., 2007; Jiang et al., 2018). Further, we analyzed the largest publicly available pharmacogenomics database to predict the chemotherapeutic response for each sample [the Genomics of Drug Sensitivity in Cancer (GDSC), https://www.cancerrxgene.org/]. Our prediction process was realized by “pRRophetic” package (Geeleher et al., 2014).

### Independence and Importance of the IPM From Traditional Clinical Features

All samples with complete clinical information, including age, gender, tumor location, tumor, node, and metastasis (TNM) stage system, regimen, PFS, and OS, were subjected to subsequent analyzes. Further, univariate and multivariate Cox regression analyzes were conducted to validate whether the predictions of the prognostic model were independent of traditional clinical features for patients with mCRC. A decision curve analysis (DCA) was used to compare the prediction performance of IPM with traditional clinical features.

### The Clinical Value of IPM in a Real-World Cohort (CICAMS CRLM Cohort)

IPM risk score of each patient was calculated with the expression of *CDKN2A, SLIT2*, and *CLU* based on IPM formula. By using the X-tile program, the optimal cutoff of risk score was determined to stratify patients at low or high risk for IPM. Continuous variables were transformed into categorical ones based on their cutoff value for recurrence within 6 months in ROC. All variables were presented as frequency (%). The association of clinicopathological factors with IPM risk levels was assessed by means of logistic regression analysis. Variables significant on bivariate analysis were subsequently included in the multivariable logistic regression model, and a stepwise selection method was used (input selection method). The prognostic value of IPM was tested by K–M analysis in CICAMS colorectal liver metastasis (CRLM) cohort.

### Investigate the Upstream Potential Reason for the Dysregulation of Three IPM Genes

Transcription factor (https://amp.pharm.mssm.edu/chea3/#top) enrichment analyses was performed to identify putative transcription factors involved in regulating three IPM immune genes in core pathway analysis using interactions experimentally verified in human tissues. The putative transcription factor was investigated preliminarily by bioinformatics.

### Statistical Analysis

All statistical tests were executed in RStudio software (running environment R 3.5.2), GraphPad Prism 8.0, SPSS 25, and Xtile 3.6.1. The R packages used in this study were survival, forestplot, glmnet, rms, foreign, survminer, regplot, stdca, timeROC, caret, mice, randomForest, ROCR, e1071, kernlab, rpart, pec, party, doMC, randomForestSRC, sva, edgR, GOSemsim, RobustRankAggreg, pRRophetic, ggplot2, cowplot, pheatmap, ggDCA, and so on. For all statistical analyzes, a *P* < 0.05 was considered statistically significant.

## Results

### Differentially Expressed Genes to Identify Genes Involved in Metastasis

**Figure 1:**
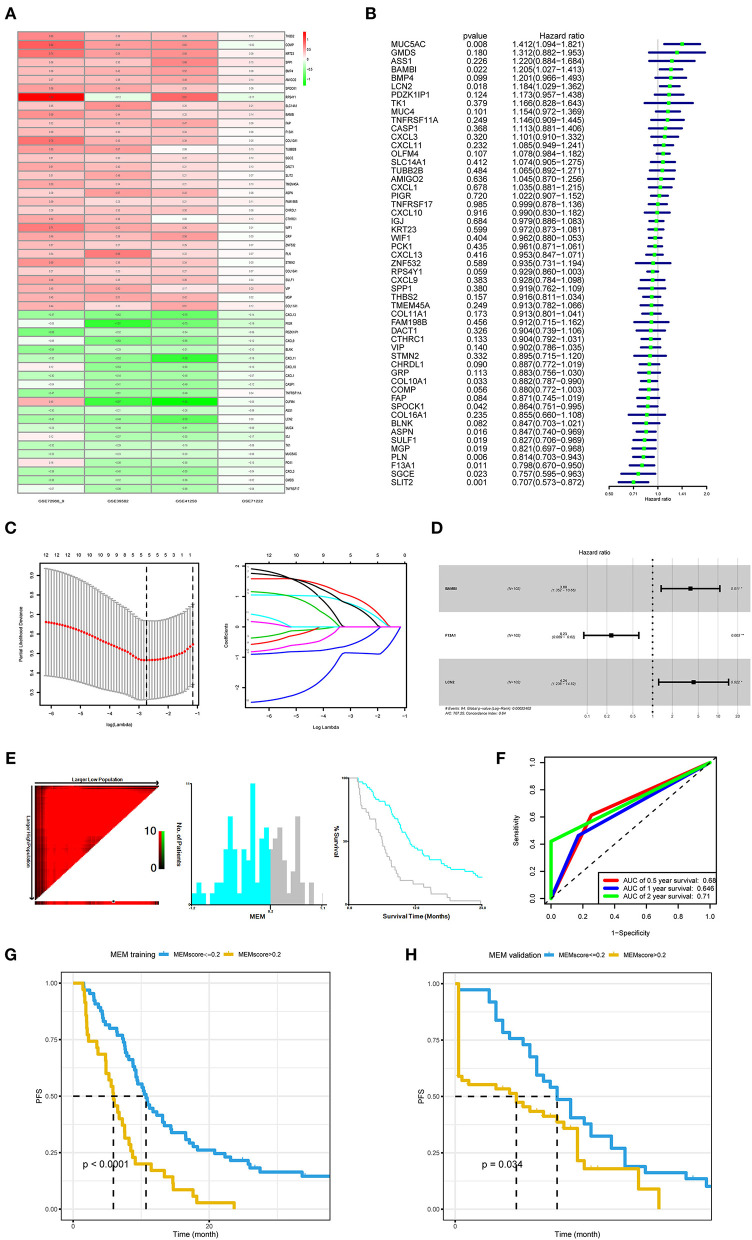
Development and validation of the metastasis evaluation model (MEM). **(A)** American Joint Committee on Cancer (AJCC) M1 stage colorectal cancer samples and AJCC M0 stage colorectal cancer samples comprehensively compared to identify differentially expressed genes (DEGs) using robust rank aggregation (RobustRankAggreg) R package, and the thresholds were |log2-fold change (FC)| >1.0 and false discovery rate (FDR) <0.05. **(B–D)** Univariate Cox, least absolute shrinkage and selection operator (LASSO), and multivariate Cox regression analyses were employed to investigate the correlation between the patient's progression-free survival (PFS) and DEGs of M1 colorectal cancer. **(E)** The optimal cutoff value (−0.2) of the MEM level found using X-tile 3.6.1 software (Yale University, New Haven, CT, USA). **(F)** Time-dependent receiver operating characteristic curve (ROC) analysis conducted to evaluate the predictive power of the prediction model. **(G)** The Kaplan–Meier (K–M) survival curves for cases with a low or high metastasis risk in training cohort produced to show MEM's prediction ability (*P* < 0.0001). **(H)**. The K–M survival curves for cases with a low- or high-metastasis risk in the MEM validation cohort approve MEM's prediction ability (*P* = 0.034).

There were 53 (32 upregulated, 21 downregulated) differentially expressed genes between American Joint Committee on Cancer (AJCC) M1 stage colorectal cancers vs. AJCC M0 stage colorectal cancers from a comprehensive analysis of the microarray datasets with RobustRankAggreg methods (Figure 1A, Supplementary Table 1).

### The MEM Predicts the PFS of mCRC Patients After Surgery

**Figure 2:**
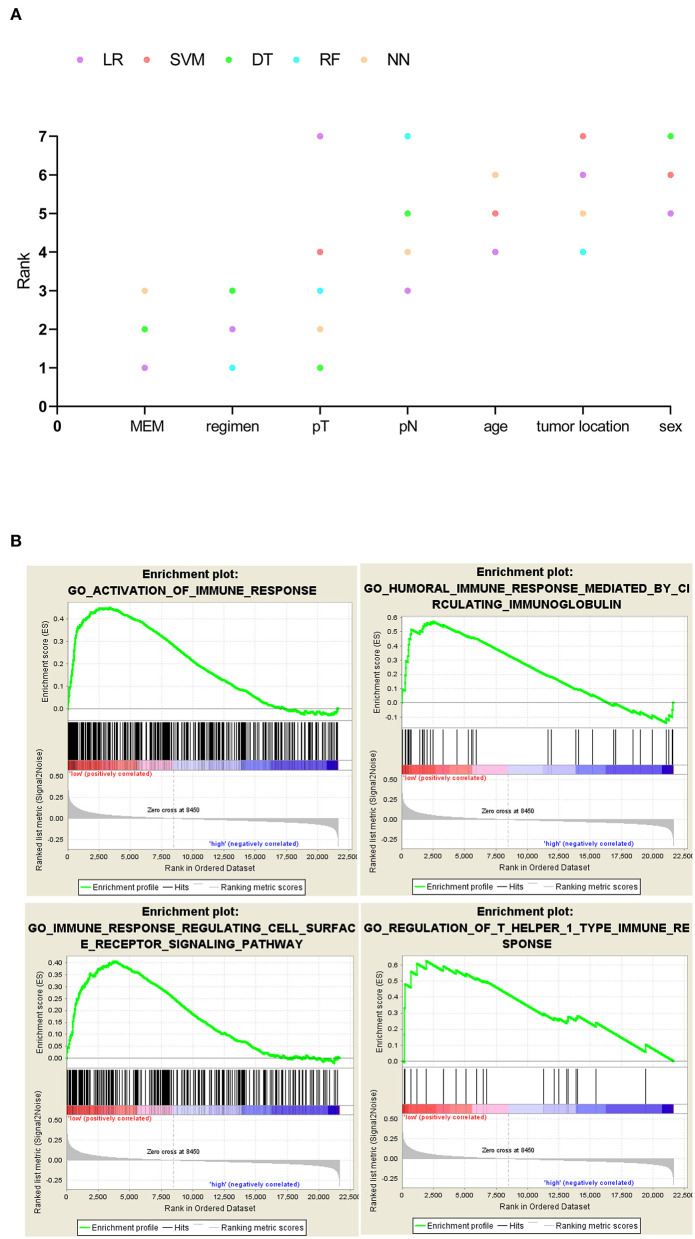
**(A)** Five machine learning variable evaluators to test metastasis evaluation model's (MEM's) predictive importance for 1-year recurrence. MEM ranked above the other common clinicopathological characteristics such as the American Joint Committee on Cancer (AJCC) stage. **(B)** MEM-high-risk metastatic colorectal cancers (mCRCs) suppressed in immune-related biological processes.

In the training cohort (*N* = 102), with three prognostic metastasis-related genes, *BAMBI, F13A1*, and *LCN2* (Figures 1B–D), MEM was used for stratifying the mCRC into high- and low-recurrence risk clusters with a forum, MEM level = (0.2613^*^ normalized expression level of *BAMBI*) + (−0.3311^*^ normalized expression level of *F13A1*) + (0.2836 ^*^ normalized expression level of *LCN2*). The X-tile diagrams produced the optimal cutoff value (=0.2) for the MEM level (Figure 1E). Thirty-seven patients with mCRC (MEM level > 0.2) were classified as MEM-high risk cluster, while the other 65 (MEM level ≤ 0.2) were assigned to the low-risk cluster. The AUCs for MEM were 0.68, 0.646, and 0.71 for 0.5, 1, and 2-year PFS rates, respectively, indicating high sensitivity and specificity for MEM (Figure 1F). The K–M PFS curves of these clusters were significantly different (*P* < 0.0001; Figure 1G). Further, in the validation cohort, MEM could stratify different risk clusters with the same cutoff value (Figure 1H). Moreover, the five machine learning variable evaluators suggested MEM as the top-ranked among common clinicopathological characteristics, such as the T/N stage in the AJCC stage system (Figure 2A), indicating high predictive performance and good clinical value of MEM.

### GSEA Predicts a Positive Association Between Immune Phenotype and MEM Subgroups

While the pathogenic role of the three MEM genes in cancer prognosis has been demonstrated previously, their combined effect on the immune profile of mCRC has not been studied. The GSEA analysis of mCRC samples indicated that the MEM low-recurrence risk cluster (MEM-low) was significantly enriched with 292 biological processes, including 33 immune-related biological processes, of which 4 classic immune processes were ACTIVATION OF IMMUNE RESPONSE, HUMORAL IMMUNE RESPONSE MEDIATED BY CIRCULATING IMMUNOGLOBULIN, IMMUNE RESPONSE REGULATING CELL SURFACE RECEPTOR SIGNALING PATHWAY, and REGULATION OF T HELPER 1 TYPE IMMUNE RESPONSE (Figure 2B, Supplementary Table 2). However, the MEM high-recurrence risk cluster (MEM-high) was enriched only in four immune-related biological processes. Thus, MEM-low mCRC could be considered to have a more activated immune phenotype (Supplementary Table 2).

### IPM Predicts the PFS of mCRC Based on Immune Status

**Figure 3:**
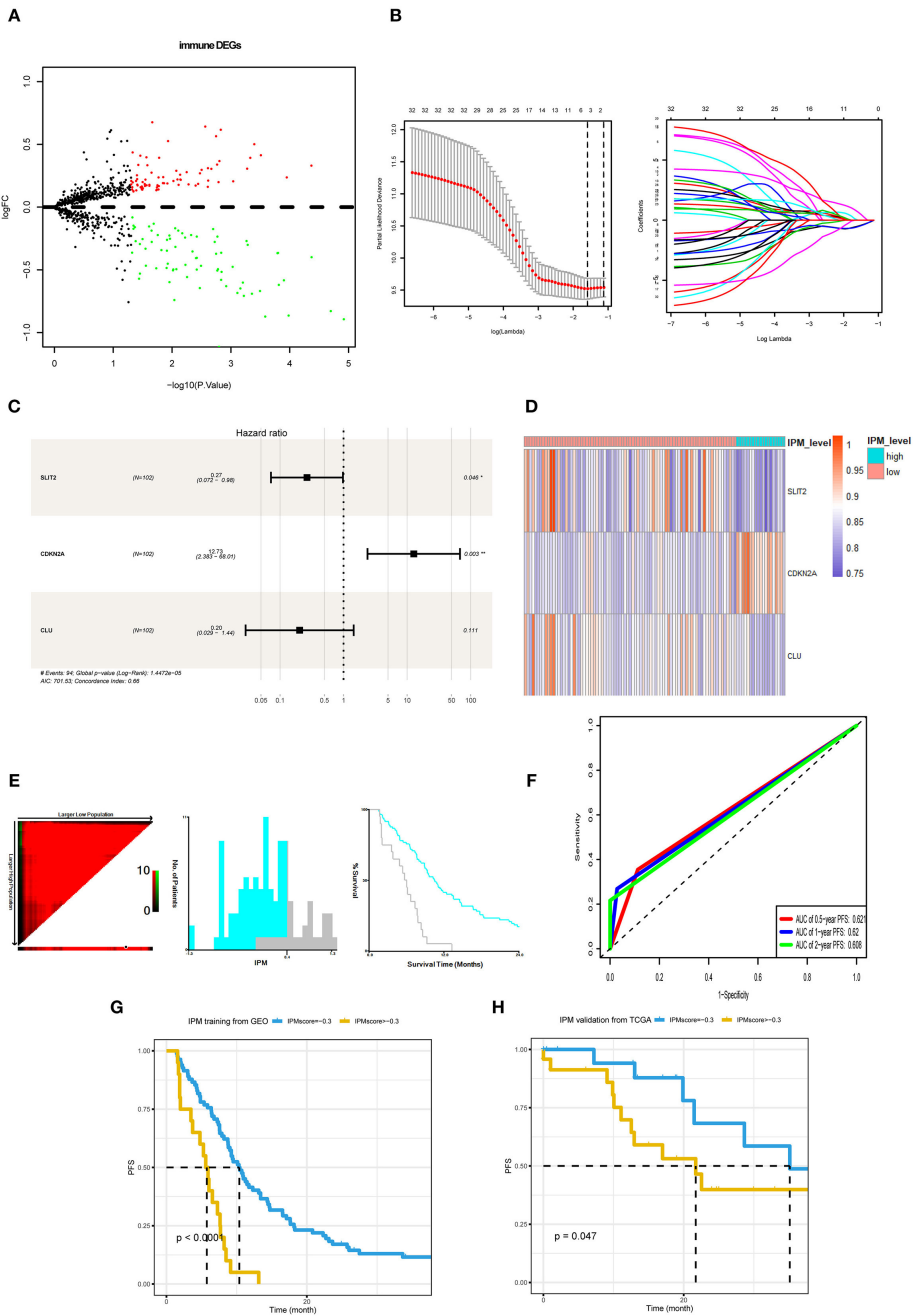
Development and validation of immune prognostic model (IPM). **(A)** Immune-related genes in the immune biological process of gene set enrichment analysis (GSEA) results compared between metastasis evaluation model (MEM)-low and MEM-high risk clusters. **(B,C)** Least absolute shrinkage and selection operator (LASSO) and multivariate Cox regression analyses were employed to investigate the correlation between the patient progression-free survival (PFS) and immune-related differentially expressed genes (DEGs). **(D)** A heat map shows the expression of *SLIT2, CDKN2A*, and *CLU* in the IPM level. **(E)** The optimal cutoff value (0.4) of the IPM score found using X-tile 3.6.1 software (Yale University, New Haven, CT, USA). **(F)** Time-dependent receiver operating characteristic (ROC) curve analysis was conducted to evaluate the predictive power of the prediction model. **(G)** The K–M survival curves for cases with a low or high metastasis risk in training cohort produced to show IPM's prediction ability (*P* < 0.0001). **(H)** The K–M survival curves for cases with a low- or high-recurrence risk in the Cancer Genome Atlas (TCGA) validation cohort approve IPM's prediction ability (*P* = 0.047).

Considering that the recurrence risk might be related to the immune status, with immune genes in the above differentially enriched GSEA processes, we identified three prognostic immune-related genes, *viz. CDKN2A, SLIT2*, and *CLU* (Figures 3A–D). Further, we developed an IPM to predict PFS of mCRC patients (IPM risk score = normalized expression level of *CDKN2A* ^*^ 2.544 + normalized expression level of *SLIT2* ^*^ (−1.327) + normalized expression level of *CLU* ^*^ (−1.5929). The Xtile software derived a cutoff point (0.4) in the training cohort to classify patients into IPM-low- and high-recurrence risk groups across all mCRC cases (Figure 3E). The AUCs for PFS were 0.68, 0.646, 0.71, 0.70, and 0.692 at 6 months, 1, 2, 3, and 5 years, respectively (Figure 3F). The IPM high-risk group had a shorter PFS than the low-risk group (Figure 3G). Moreover, in the TCGA mCRC validation cohort, the IPM risk score could also stratify different risk groups with its best cutoff value (−0.3) (Figure 3H).

**Figure 4:**
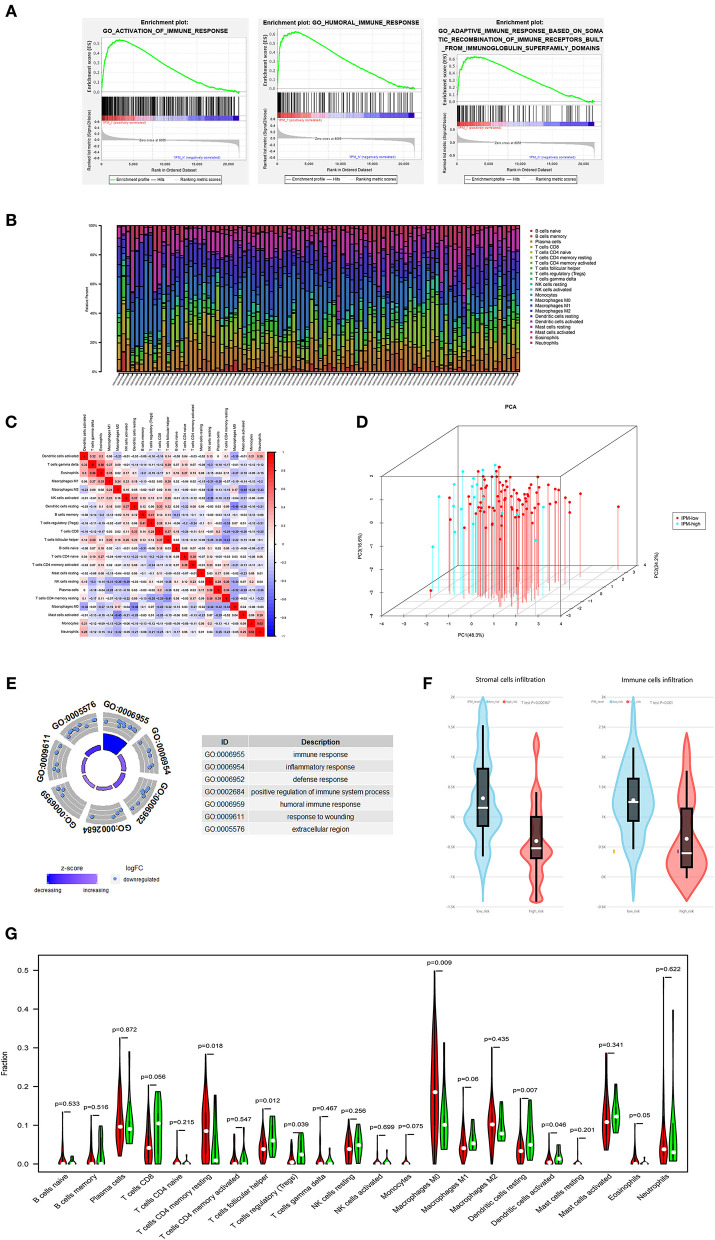
**(A)** The local immune status may confer an intense immune phenotype in the IPM-low-risk group and a weakened immune phenotype in the IPM-high-risk group. **(B)** Within and between groups, the proportion of immune cells in metastatic colorectal cancers (mCRCs) varies. Therefore, variations in the proportions of tumor-infiltrating immune cells might represent an intrinsic feature that could characterize individual differences. **(C)** Proportions of some subpopulations of tumor-infiltrating immune cells are correlated. **(D)** The samples of IPM low- and high-risk mCRCs patients clearly separated into two discrete groups based on principal component analysis, indicating that two groups are distinctly different in immune infiltrating cells. **(E)** IPM high-recurrence risk mCRCs are at immunosuppression status. **(F)** The Estimation of Stromal and Immune cells in Malignant Tumor tissues Using Expression Data (ESTIMATE) algorithm showing that the stroma and immune cells infiltration in IPM high-risk groups are less than those in the low-risk group. **(G)** The IPM high-risk mCRCs have significantly higher proportions of Tregs and lower proportions of resting memory CD4+ T cells.

Further, the GSEA between the 83 IPM low-risk and 19 IPM high-risk mCRC in the training cohort revealed that the low-risk group was associated with 35 immune-related biological processes, such as (the top 5): GO_HUMORAL_IMMUNE_RESPONSE (NES = 2.54,size = 144), GO_ADAPTIVE_IMMUNE_RESPONSE_BASED_ON_SOMATIC_RECOMBINATION_OF_IMMUNE_RECEPTORS_BUILT_FROM_IMMUNOGLOBULIN_SUPERFAMILY_DOMAINS(NES = 2.51, size = 123), GO_HUMORAL_IMMUNE_RESPONSE_MEDIATED_BY_CIRCULATING_IMMUNOGLOBULIN (NES = 2.49, size = 38), GO_ADAPTIVE_IMMUNE_RESPONSE (NES = 2.48, size = 246), GO_ACTIVATION_OF_IMMUNE_RESPONSE (NES = 2.42, size = 380) (*P* < 0.05; Figure 4A, Supplementary Table 3). On the contrary, the IPM high-risk mCRC did not associate with any immune-related processes. Hence, IPM could indicate the local immune status of mCRC, where an intense immune phenotype associated with low-risk mCRC and a weakened immune phenotype with high-risk mCRC.

**Figure 5:**
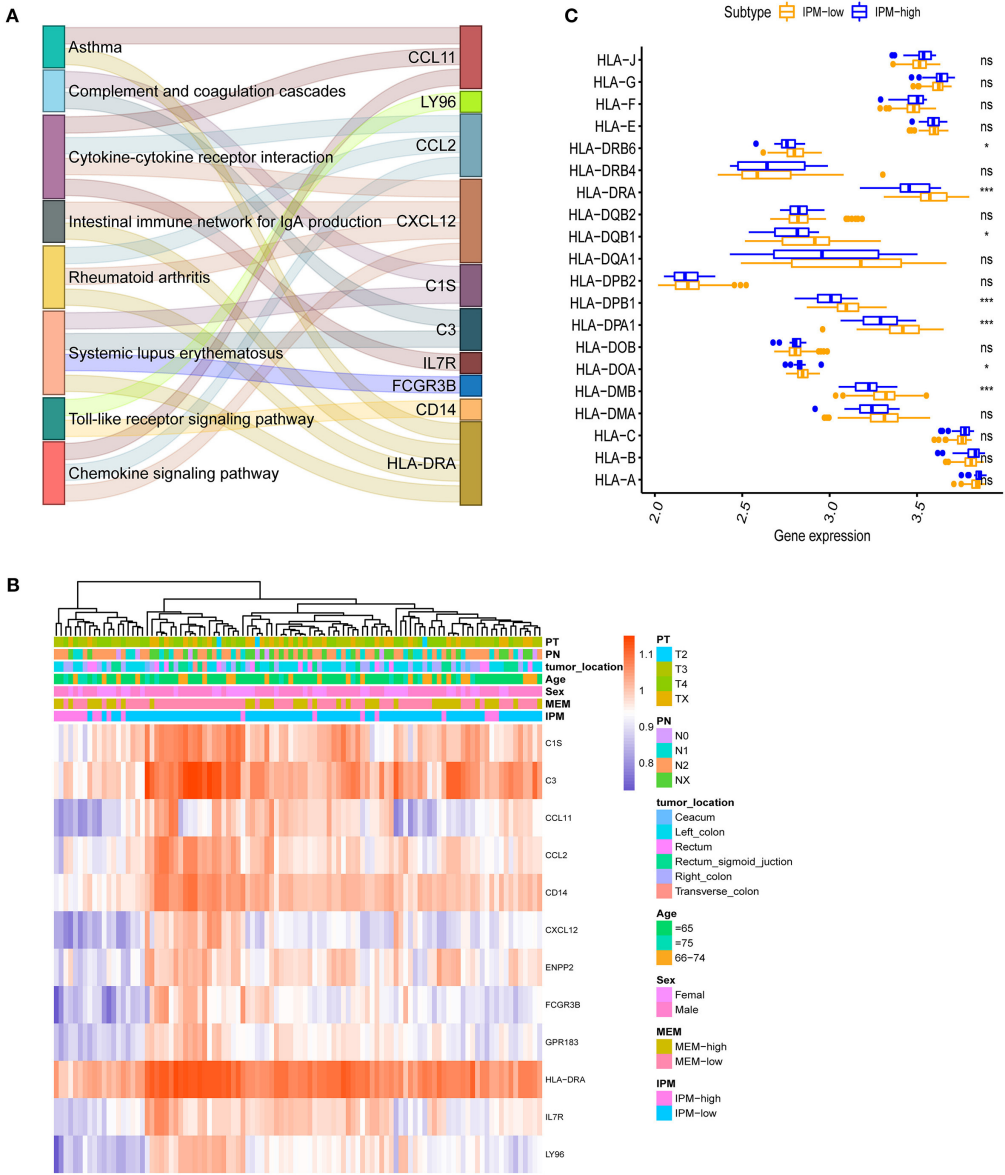
**(A)** Immune differentially expressed genes (DEGs) in the immune prognostic model (IPM) criterion. The immune genes are differentially expressed between the groups at IPM low risk and high risk for metastatic colorectal cancers (mCRCs) (*P* < 0.05) and 12 genes identified and subjected to KEGG analyses to find that the immune genes related to the IPM in the training dataset are mainly enriched in the immune response and immune system diseases pathway. **(B)** *HLA-DRA* is downregulated in IPM-high group mCRCs. **(C)** Major histocompatibility complex (MHC) class-II molecules are all downregulated in IPM-high group mCRCs.

In the GO and KEGG enrichment analysis, the immune genes related to IPM in the training cohort were mainly enriched in the immune response biological process and immune system disease pathway (Figures 4E, 5A). Additionally, the human leukocyte antigen DR isotype (HLA-DR) was found to be downregulated in the IPM-low group (Figure 5B). According to HLA-DRA's alteration and considering it belongs to HLA family, the expression of the HLA can affect T-cell recognition of tumor antigen (Rooney et al., 2015) and influence the local immune status. Therefore, we analyzed the expression of all HLA subtype genes between low- and high-risk IPM groups and found that the expression of MHC class I molecules (*HLA-A, HLA-B*, and *HLA-C*) were not significantly different, whereas the MHC class II molecules, *HLA-DR, HLA-DP*, and *HLA-DQ* were all downregulated in the IPM high-risk group (Figure 5C), indicating the ignored function of MHC class II molecules in mCRC's immune status.

### Immune Landscape of IPM Groups

Our analysis suggested that the proportion of immune cells in mCRC varies within and between the groups (Figure 4B), and the proportions of some subpopulations of tumor-infiltrating immune cells are correlated (Figure 4C), indicating that changes in the proportion of TICs may represent intrinsic characteristics that can describe the individual differences. Moreover, the IPM high-risk mCRC showed significantly higher infiltrating proportions of Tregs, T follicular helper cells, and resting dendritic cells, and lower proportions of CD4+ memory T cells and resting macrophages (M0), than the low-risk mCRC patients (*P* < 0.05; Figure 4G). Additionally, the ESTIMATE algorithm showed lesser infiltration of stroma and immune cells in the IPM high-risk group than in the IPM low-risk group (*P* = 0.000167 and *P* = 0.001, respectively), attributing the immunosuppressive microenvironment to poor outcomes in high-risk patients (Figure 4F). Furthermore, the samples of IPM groups could be divided into two discrete spot groups based on the principal component analysis (Figure 4D). Therefore, these results indicate that an abnormal immune infiltration and its heterogeneity in mCRC samples can be used as prognostic indicators and immunotherapy targets and have important clinical significance. Furthermore, the risk of mCRC recurrence was related to the immune phenotype.

### Sensitivity of the IPM Subtypes to Immuno-/Chemotherapy

**Figure 6:**
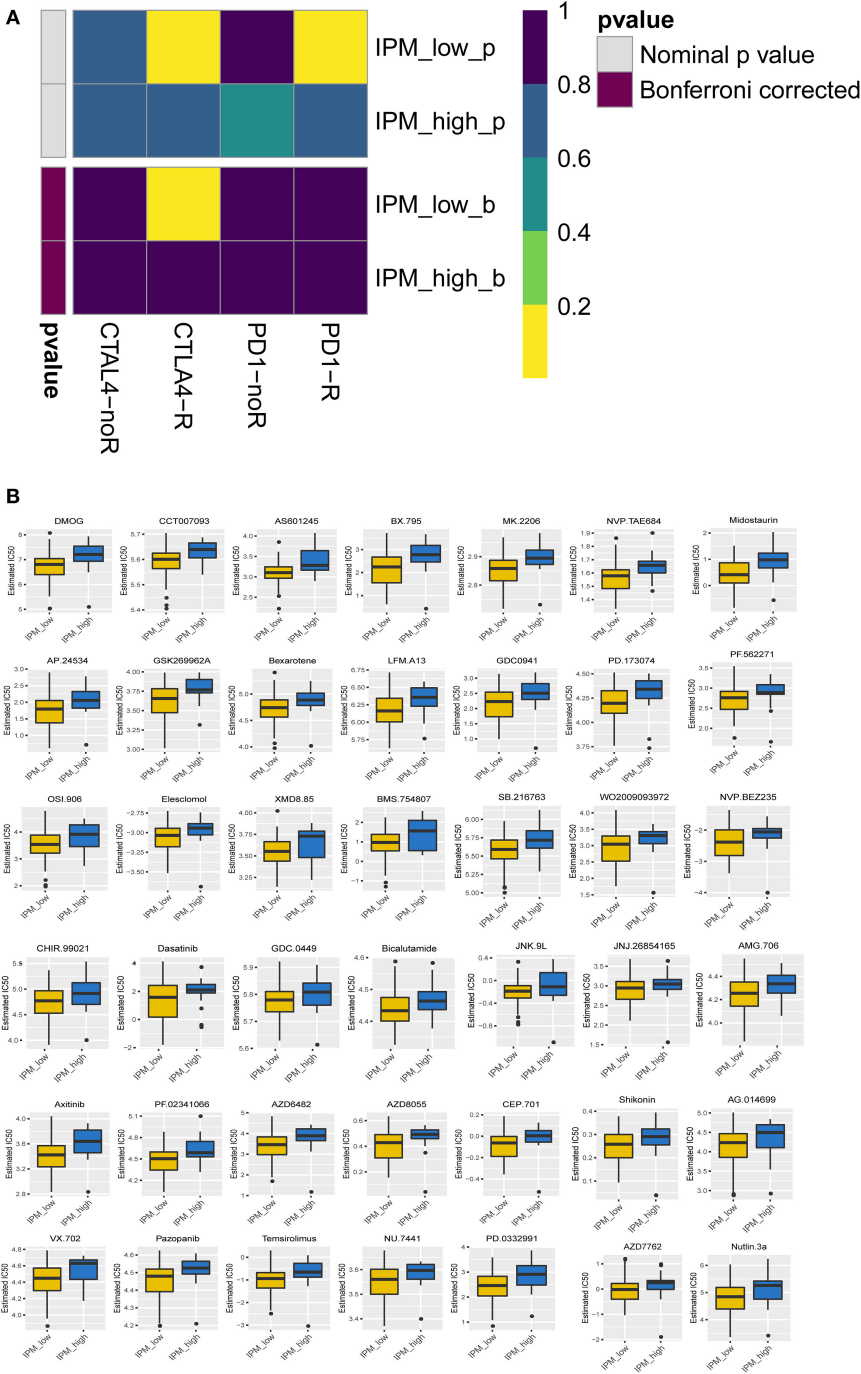
Differential putative chemotherapeutic and immunotherapeutic response. **(A)** The immune prognostic model (IPM)-low group has a more promising response to anti-CTLA-4 therapy (Bonferroni corrected *P* = 0.005). **(B)** IPM-low group is more sensitive to targeted drugs.

The TIDE algorithm was employed to predict the likelihood of response to anti-PD-1 and anti-CTLA-4 immunotherapy, although the results demonstrated no difference in response to immunotherapy between the IPM-low (39/83) and IPM-high (7/19) samples (*P* = 0.456). Considering the lower accuracy of predicting the response in colorectal cancers than in melanomas (as described in the TIDE introduction), we utilized a subclass mapping algorithm to compare the RNA profiles of the IPM-risk groups with another published dataset containing 47 cases of melanoma that responded to anti-PD-1 and anti-CTLA-4 immunotherapies (Roh et al., 2017). The results revealed that the IPM-low group was more likely to respond to anti-CTLA-4 immunotherapy (Bonferroni-corrected *P* = 0.005), but the IPM-high group was not sensitive to these immune checkpoint inhibitors (Figure 6A). Further, the IC50 values of the IPM-low and IPM-high groups were predicted with the GDSC data, and our analysis indicated no targeted drugs with a significant response sensitivity against the IPM-high group (Figure 6B).

### The Correlation Between Immune Checkpoint Modulators and IPM Recurrence Risk Groups

**Figure 7:**
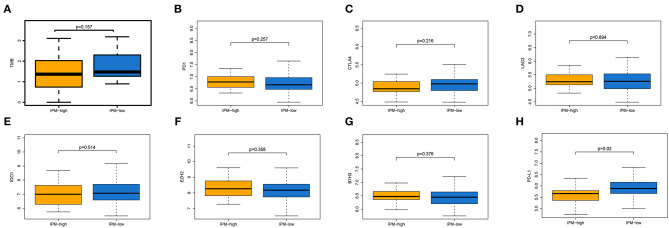
Expression of immune inhibiting factors between two immune prognostic model (IPM) recurrence risk groups. The TMB **(A)**, and the expression levels of *PD-1* **(B)**, *CTLA-4* **(C)**, *LAG3* **(D)**, **IDO (E)**, **EZH2 (F)**, and *B7H3* **(G)** were not significantly different between the two IPM recurrence risk groups (*P* > 0.05), but PD-L1 **(H)** was differently significant (*P* = 0.02).

Immune checkpoint proteins play a vital role in cancer immunotherapy. The difference in immune checkpoint modulators between IPM low- and high-risk groups with mCRC was estimated. Modulators *B7H3, LAG3, TIM-3, CTLA-4, PD-1*, and *IDO* were not significantly different between the two IPM recurrence risk groups (*P* > 0.05; Figures 7A–G), but PD-L1 was different significantly (*P* = 0.02; Figure 7H), indicating that the suppressed immune status might not be mainly influenced to immune checkpoint modulators.

### The IPM Is Independent of Conventional Clinical Characteristics With Better Net Benefits in Clinical Practice

**Figure 8:**
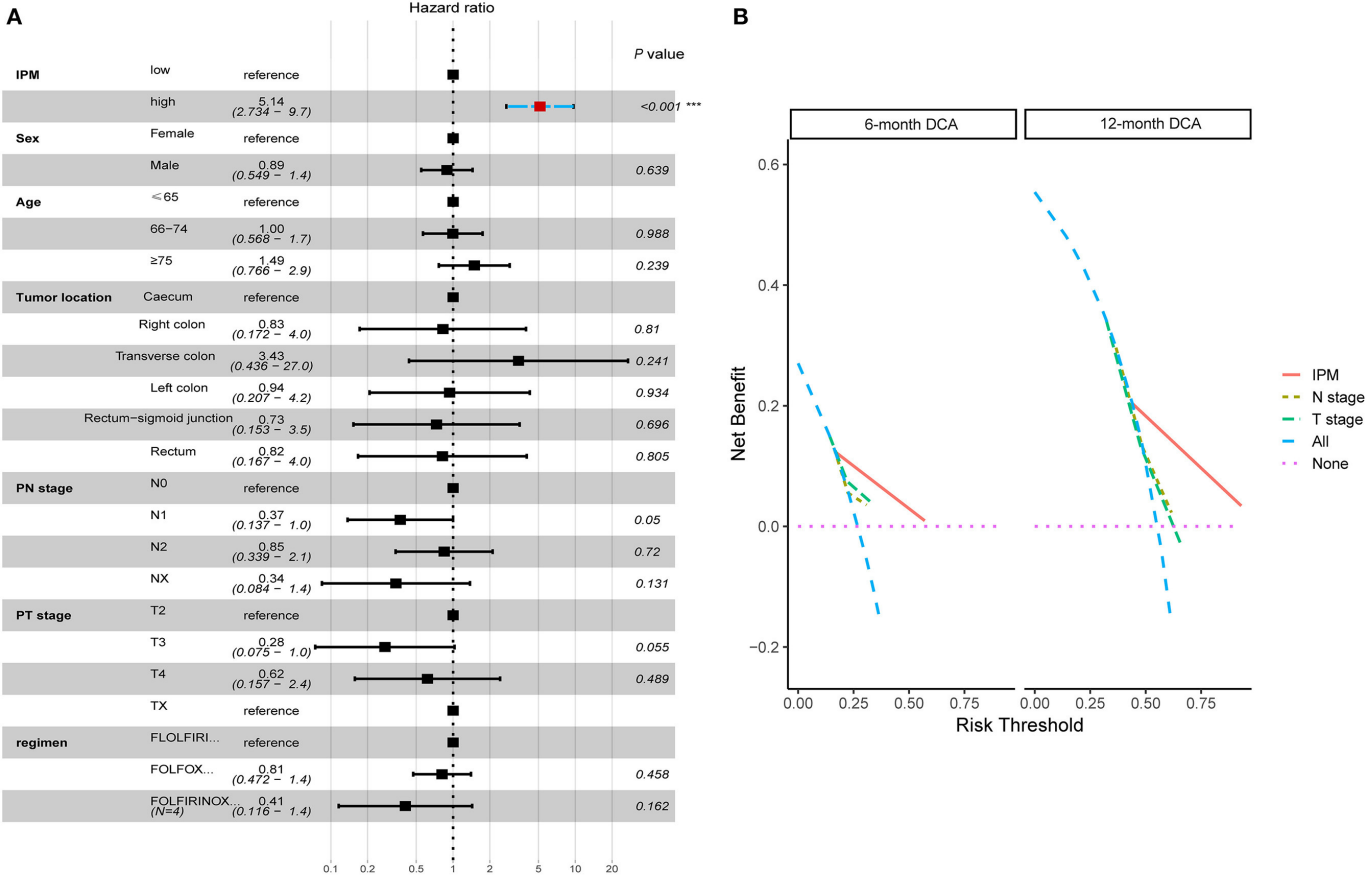
The immune prognostic model (IPM) is independent of conventional clinical characteristics with better net benefits in clinical practice. **(A)** The multivariate Cox regression analysis indicated that the IPM was significantly correlated with the survival information (*P* < 0.001) and the highest median risk score (HR = 8.45, 95% CI = 3.987–17.90). **(B)** The net benefits of the IPM outperforms other conventional clinical characteristics.

The univariate and multivariate Cox regression analyzes were conducted to explore whether the prognostic value of the IPM was independent of other clinical factors in the training cohort. After adjusting for clinical characteristics, the IPM continued to be an independent prognostic factor, thus confirming its robustness for predicting the recurrence of mCRC (Figure 8A). Additionally, the multivariate Cox regression analysis indicated that the IPM was significantly correlated with the survival information (*P* < 0.001) and the highest median risk score (HR = 8.45, 95% CI = 3.987–17.90) (Figure 8A). Furthermore, the DCA compared the net benefits of the IPM and conventional clinical characteristics (Figure 8B). Collectively, these results indicated that the IPM was independent of conventional clinical characteristics and performed better than conventional clinical characteristics to predict survival.

### IPM Stratifies a Refractory CRLM Subtype in a Real-World Cohort

**Figure 9:**
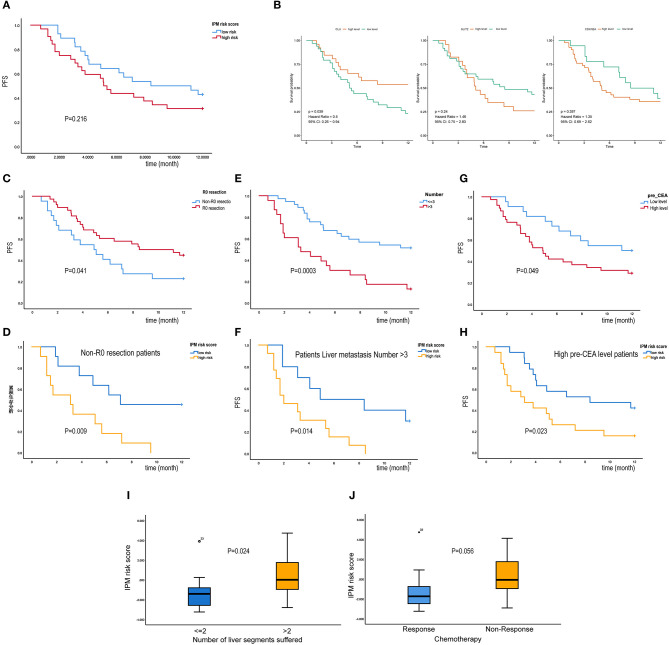
Immune prognostic model (IPM) stratifies a refractory colorectal liver metastasis (CRLM) subtype in a real-world cohort (CICAMS CRLM cohort). **(A)** A difference trend in PFS was found between the high- and low-risk group although without significance (*P* = 0.216). **(B)** K–M survival curves of three IPM genes. **(C–H)** When applied for subcategories of patients with commonly known high-recurrence risk [liver metastasis number >3, high preoperative carcinoembryonic antigen (CEA), and non-R0 resection] at the time of diagnosis, the IPM risk score was predictive of significantly different PFS (*P* < 0.05). **(I)** IPM risk score was significantly higher in patients with more than 2 liver segments suffered (*P* = 0.024). **(J)** The relation between IPM risk score and Chemotherapy response, and non-response group tends to have a higher IPM risk score (*P* = 0.056).

To evaluate the robustness of IPM in predicting the risk of tumor recurrence for patients with mCRCs in clinical practice, we used qRT-PCR to further validate the specific signature in an independent cohort consisting of frozen tissue samples from 60 patients with CRLMs. The detail of clinical characteristics of this independent cohort are shown in Table 1. Using the same formula, the risk score of each patient was calculated. Patients were also divided into high- and low-risk groups (*N* = 32 and 28, respectively) by the given risk score, and the cutoff point was chosen through optimized risk value (−0.5). A difference trend in PFS was found between the high- and low-risk group although without significance (*P* = 0.216; Figure 9A). The Kaplan-Meier curve shows the prognostic relation with expression of *CLU* (HR = 0.5, *P* = 0.039), *SLIT2* (HR = 1.46, *P* = 0.24) and *CDKN2A* (HR = 1.35, *P* = 0.397) (Figure 9B). Further, when applied for subcategories of patients with commonly known high-recurrence risk [liver metastasis number >3, high preoperative carcinoembryonic antigen (CEA), and non-R0 resection] at the time of diagnosis, the risk score was predictive of significantly different PFS (*P* < 0.05; Figures 9C–H). Subsequently, we explored its relationship with clinicopathological variables. IPM risk score was significantly higher in patients with more than 2 liver segments suffered (*P* = 0.024, Figure 9I). We found that high-risk patients had a significant non-response to chemotherapy than the low-risk group in CICAMS CRLM cohort (*P* = 0.056, Figure 9J; *P* = 0.03, Table 1).

**Table 1:** Comparison of baseline characteristics and operative variables between patients with IPM low-risk and IPM high-risk in CICAMS CRLM cohort.

**Clinicopathological variable**	**IPM low-risk (*N* = 28)**	**IPM high-risk (*N* = 32)**	**Stat**	***P*-value**
Gender			0.904	0.342
Male	15	21		
Female	13	11		
Age				
≤ 48	4	10	2.402	0.121
>48	24	22		
Recurrence within 1 year			0.866	0.352
No	12	10		
Yes	16	22		
Primary tumor location			0.336	0.562
Left colon cancer	23	28		
Right colon cancer	5	4		
Liver metastasis segments suffered Number			6.548	0.01
≤ 2	18	10		
>2	10	22		
T stage			2.2946	0.4
T1	0	2		
T2	26	26		
T3	2	3		
T4	0	1		
N stage			3.802	0.149
N0	9	5		
N1	7	15		
N2	12	12		
Number of liver metastasis			0.152	6.96E-01
≤ 3	18	19		
>3	10	13		
Double lobes suffered			0.693	0.405
No	17	16		
Yes	11	16		
Size of liver metastasis			0.152	0.696
≤ 3 cm	18	19		
>3 cm	10	13		
Differentiation			0.155	0.925
Well	17	21		
Moderate	4	4		
Low	7	7		
Extrahepatic metastasis			0.39	0.533
No	25	30		
Yes	3	2		
Pre-operative CEA			0.463	0.496
≤ 5.96	9	13		
>5.96	19	19		
Pre-operative CA19-9			0.067	0.796
≤ 79.7	22	26		
>79.7	6	6		
Post-operative CEA			0.106	0.744
≤ 38.84	22	24		
>38.84	6	8		
Post-operative CA19-9			0.021	0.885
≤ 92.1	24	27		
>92.1	4	5		
Chemotherapy			0.005	0.944
Without	12	14		
With	16	18		
With chemotherapy				
Response	9	3		0.03^*^
Non-response	7	15		
Total hospitalized day			1.071	0.301
≤ 24 d	24	24		
>24 d	4	8		
Post-operative hospitalized day			1.837	0.175
≤ 12 d	22	20		
>12 d	6	12		
Surgical procedure			4.037	0.133
laparotomy	15	9		
laparoscopy assisted	3	5		
laparoscopy	10	18		
Sequence mode			1.558	0.212
Simultaneous	27	28		
Non-simultaneous	1	4		
Total number of resected lymph nodes			0	1
≤ 22	21	24		
>22	7	8		
Positive lymph node ratio			1.558	0.212
Negative	7	4		
Positive	21	28		
Surgery time			0.001	0.972
≤ 335	13	15		
>335	15	17		
Intraoperative blood loss volume			0.805	0.37
≤ 100	8	6		
>100	20	26		
Intraoperative blood transfusion			2.084	0.149
Without	23	21		
With	5	11		
Post-operative complication			1.35	0.245
No	19	17		
Yes	9	15		
Post-operative defecation time			1.408	0.235
≤ 4 d	17	24		
>4 d	11	8		
R0 resection			0.155	0.694
No	11	11		
Yes	17	21		

### *SLIT2* Functions as the Hub Gene in Immune-Related Recurrence of mCRC

**Figure 10:**
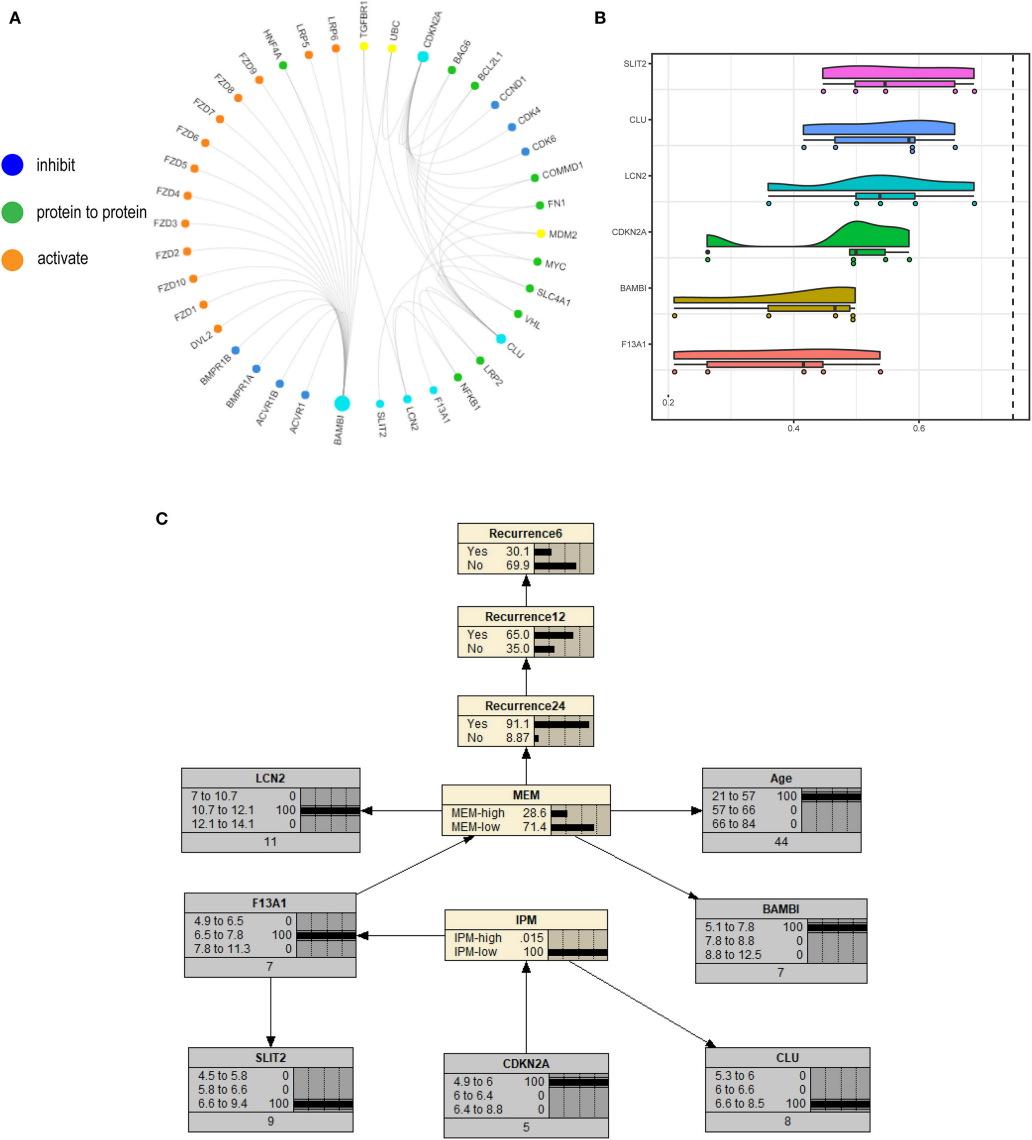
**(A)** A correlation web with these six genes based on the information from published papers is built, where they communicate with three genes (*TGF*β*R1, UBC*, and *MDM2*). **(B)** The GOSemSim analysis identified *SLIT2* to interact with the other five key genes frequently. **(C)** Bayesian network (BN) graph showing metastasis evaluation model (MEM) and immune prognostic model (IPM), using two related gene signatures for the recurrence prediction of metastatic colorectal cancers (mCRCs).

We constructed a correlation web with these six genes based on the information from published papers (Figure 10A), where they communicate with three genes (*TGF*β*R1, UBC*, and *MDM2*), revealing the possible recurrence mechanism of mCRC after integrated treatment using surgery and adjuvant chemotherapy. Further, the GOSemSim analysis identified *SLIT2* to interact with the other five key genes frequently, indicating it to be strongly associated with recurrence in mCRC and to communicate via multiple interactions and function as the hub gene (Figure 10B). We finally constructed a Bayesian network (BN) graph to show MEM and IPM, using two related gene signatures for the recurrence prediction of mCRCs (Figure 10C).

### Homeobox Gene *MEIS1* Identified as Transcription Factors of IPM Genes in mCRC

**Figure 11:**
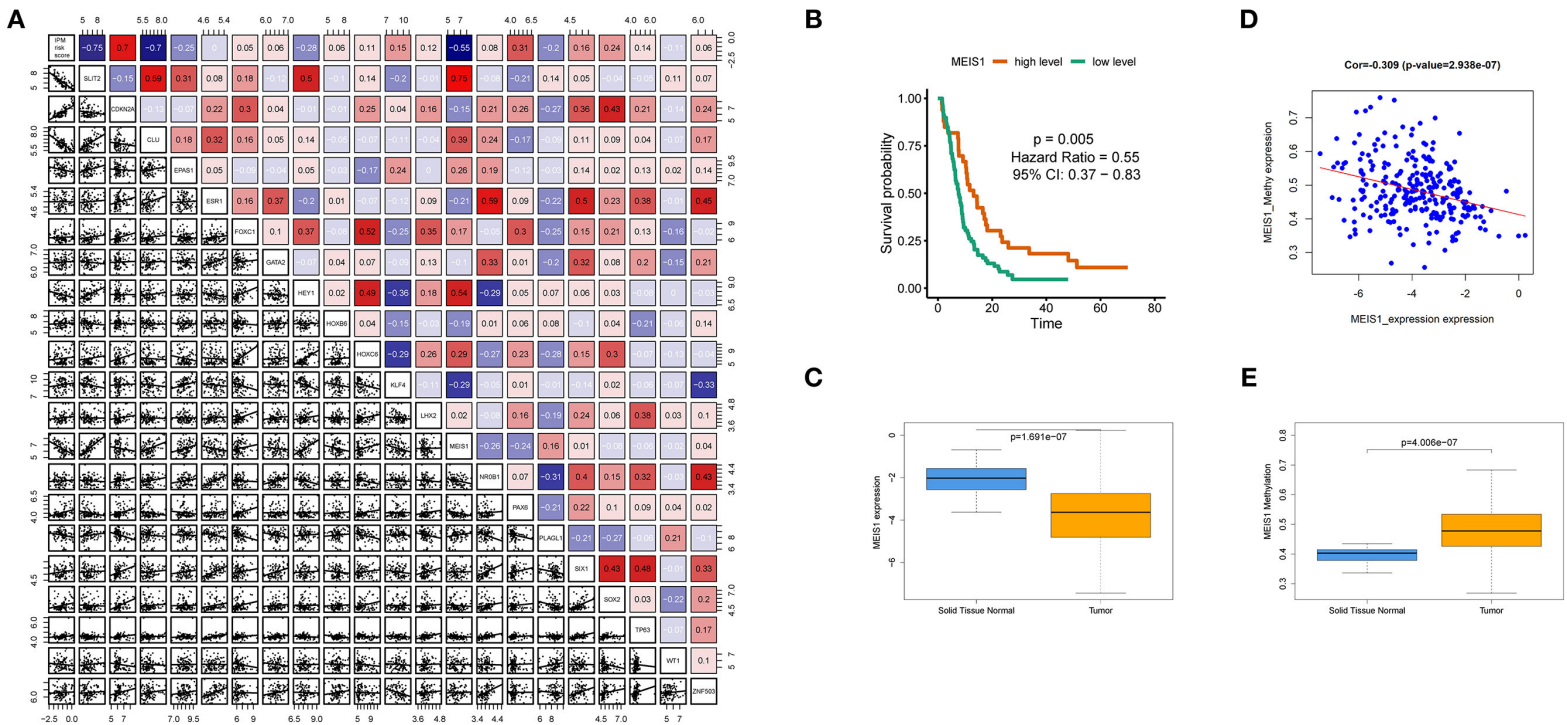
MEIS1 identified as transcription factors of immune prognostic model (IPM) genes in metastatic colorectal cancer (mCRC). **(A)** Putative transcription factors involved in regulating IPM genes expression; only *MEIS1* showed a positive correlation with *SLIT2* and *CLU* expression and a negative correlation with *CDKN2A* simultaneously (*P* < 0.05). **(B)** Decreased *MEIS1* level associated with worse progression-free survival (PFS) (HR < 0.55, *P* = 0.005). **(C)** MEIS1 level was significantly downregulated in the tumor tissue relative to the solid tissue normal (*P* < 0.001). **(D)** Expression of *MEIS1* has a negative association with *MEIS1* methylation (*P* < 0.05). **(E)** *MEIS1* methylation was more in tumor than the normal tissue in colon cancer (*P* < 0.001).

To investigate upstream regulation of the three IPM genes (*CDKN2A, SLIT2*, and *CLU*) showing prognostic efficacy, we performed bioinformatics enrichment analysis to identify putative transcription factors involved in regulating IPM genes expression. A total of 19 transcription factors (*ZNF503, KLF4, PLAGL1, PAX6, TP63, HOXB6, MEIS1, GATA2, SIX1, EPAS1, CTCF, NR0B1, SOX2, ESR1, HEY1, HOXC6, FOXC1, WT1*, and *LHX2*) were identified to be shared by three genes, with all but *CTCF* used for further analysis because there is no *CTCF* probe in the available data. We found that MEIS1 showed a stronger correlation with IPM risk score relative to the other transcription factors (Rho value = −0.55). Moreover, *MEIS1* showed a positive correlation with *SLIT2* and *CLU* expression and a negative correlation with *CDKN2A* (Figure 11A). Additionally, expression profiles and survival analyses revealed that *MEIS1* level was significantly downregulated in tumor tissue relative to solid tissue normal (*P* < 0.001; Figure 11C), with decreased MEIS1 level associated with worse PFS (HR < 0.55, *P* = 0.005; Figure 11B) in mCRC. Furthermore, we preliminarily used methylation data in TCGA to investigate the downregulation cause of *MEIS1* in colon cancer and found that the expression of *MEIS1* has a negative association with *MEIS1* methylation (Figure 11D); what is more, *MEIS1* methylation was more in tumor than normal tissue in colon cancer (*P* < 0.001; Figure 11E). Therefore, we hypothesized that *MEIS1* works as a transcription factor that mediates IPM genes and that, with methylated, *MEIS1* was downregulated, which could not thoroughly stimulate the expression of favored IPM genes (*SLIT2* and *CLU*), while that releases the expression of unfavored IPM gene (*CDKN2A*), thereby contributing to the initiation, development, and progression of colon cancer and worse prognosis.

## Discussion

Accumulating evidence suggests that bioinformatics analysis would be an effective method to find novel molecular biomarkers in early diagnosis, therapeutic process monitoring, and prognostic evaluation of cancer. Recurrence of mCRC after surgery remains a challenge, even with adjuvant chemotherapy. Moreover, the outcomes of mCRC are heterogeneous, and predicting models have failed to explain recurrence from the perspective of intrinsic cell activities and extrinsic immune microenvironment. Therefore, gene signatures with explainable recurrence associations might be of considerable benefit to the medical community.

Here, we identified two interactive gene signatures that predicted mCRC recurrence values. Further, IPM could help understand recurrence from intrinsic and extrinsic factors perspectives and identify responders of immune checkpoint inhibitors (ICIs) and chemotherapy. Moreover, it highlighted the suppression of MHC class II as the main reason for non-responsiveness. Further, in a real-world cohort, we validated these findings in specimen experiment *in vitro*. With preliminary bioinformatics investigation, we found a potential upstream dysregulation cause of IPM genes. Finally, this study produced preclinical evidence for inhibiting *CDKN2A* and activating MHC class II in the immunotherapy of mCRC. In conclusion, our study embraced many hints for futural researches on the field of immune suppression in mCRCs. Moreover, this kind of study can be used as a reference for understanding other cancers' predicting gene models to filter out the models with predicting performance as well as biological mechanism meaning.

First, our results indicate a feasible therapeutic strategy to shape the immune microenvironment and improve the post-operative prognosis of mCRC and may also allow the selection of patients for surgery-based integrated therapy. Three metastasis-related genes (*BAMBI, F13A1*, and *LCN2*) and three immune-related genes (*CDKN2A, SLIT2*, and *CLU*) serve as the two gene models, which together may provide better performance than alone, depending on their prognostic significance and immune properties. The *BAMBI* (Vanhara and Souček, 2013), *LCN2* (Wang and Zeng, 2014), *F13A1* (Vairaktaris et al., 2007), *CDKN2A* (Exner et al., 2015), *SLIT2* (Chen et al., 2013), and *CLU* (Shapiro et al., 2015) were reported to individually play a role in cancer development and progression. In our study, the high expression of *CDKN2A* was associated with unfavorable immuno-phenotype in patients with mCRC, which is in contrast to its known role as a tumor suppressor gene. A plausible explanation is that from an immune perspective, *CDKN2A* encodes several transcript variants to regulate the macrophage apoptotic process (González-Navarro et al., 2010) and downregulates B-cell proliferation to influence the humoral immune response. In addition to these, *CDKN2A* was confirmed in single-cell sequencing as one of the characteristic markers of some immune infiltrating cells, such as exhausted CD4+ T cell (Zheng et al., 2017), regulatory T (Treg) cell (Zheng et al., 2017), and natural killer T (NKT) cell (Young et al., 2018). Therefore, our study also inferred that the high expression of *CDKN2A* influences the immune microenvironment, downregulates the immune activity, and thus promotes the recurrence in mCRC. Moreover, this is possibly the first analysis to indicate an association of high expression of *F13A1* with a favorable prognosis in mCRC.

Second, our results indicate a high-recurrence risk of mCRC associated with its immunosuppression, which correlates with the downregulation of MHC class II molecules, high infiltration of Treg cells, and low levels of resting memory CD4+ T cells reservation.

Next, the MEM indicated a suppressed local immune state in high recurrence risk mCRC, and hence, we investigated a MEM-related IPM immune gene signature to elucidate how it might affect the expression of immune genes. Our IPM analysis showed that the low-recurrence risk group had an activated immune response, while the high-risk group was associated with an exhausted immune state. Additionally, the ESTIMATE algorithm validated this finding, suggesting that the stroma and infiltration of immune cells in the IPM high-risk group were less than those in the low-risk group. In the Gene Ontology (GO) and KEGG analysis, the two IPM groups differed at the immune pathway enrichment level, confirming the downregulation of immune response genes in high-recurrence risk mCRC. Furthermore, the IPM high-risk group showed higher fractions of Tregs and lower resting memory CD4+ T cells. Previous studies with metastatic melanoma have shown that the infiltration of CD8+ T cells in tumors and tumor margins positively correlates with a good prognosis (Tumeh et al., 2014). However, the CD8+ T cells contains multiple subpopulations, and even if CD8+ T cells infiltrate the tumor tissue, the Tregs in the tumor may lead to no response to treatment (Ngiow et al., 2015). Previous studies have confirmed that CD4+ T cells, upon differentiation, may acquire various functions, including blocking cytotoxic NK cells and activating CD8+ T cells, suppressing harmful immunological reactions to self- and foreign antigens, and aiding CD8+ T cells in tumor rejection (Crouse et al., 2015; Rosenberg and Huang, 2018; Long et al., 2019). Moreover, the cancer immunoediting hypotheses suggest that antitumor immune response during cancer development and progression is evaded by selecting fewer immunogenic cancer cells (immune selection) and establishing immunosuppressive networks (immune escape) (Long et al., 2019). Cancer cells have several immunosuppressive mechanisms, including increasing the levels of various immunosuppressive cells, such as Treg cells and macrophages, elevating levels of various immunosuppressive molecules, and decreasing the expression of cancer antigens, which together result in the dysfunctioning of CD8+ T cells to recognize cancer cells (Pardoll, 2012; Long et al., 2019). However, expression of *PD-L1* was found to be decreased in the high-recurrence risk group, although *PD-1, CTLA-4, LAG3, IDO1, EZH2*, and *B7H3*, were not differentially expressed in this subtype. Additionally, the tumor mutation burden in the TCGA validation cohort was not altered in the two IPM groups. Therefore, to a certain extent, altered infiltrations of Tregs and resting memory CD4+ T cells could indicate a high-risk recurrence of mCRC, although it might not be the most important factor; meanwhile, such mCRC may belong to a refractory subtype. Furthermore, as indicated in drug-sensitivity data mining, the IPM-low group was more likely to have a positive response to anti-CTLA-4 immunotherapy, while the IPM-high group showed no sensitivity to these ICIs. Moreover, we observed the IPM-high group to present more therapy resistance than the IPM-low group. However, we do not know the cause for the IPM-high risk group to be the refractory mCRC subtype.

Further investigation into immune-related DEG in the IPM-high risk group found *HLA-DRA*, a component of the MHC class II, which plays a crucial role in regulating immune response with CD4+ T cells, to be downregulated in the high-recurrence risk mCRC. Given the disorders of CD4+ T cells in the IPM high-risk group, we speculated that its helper immune response might be affected. Therefore, we assessed the expression of HLA in the low- and high-risk IPM groups. Our analysis implicated the expression of the MHC class-II molecules, *viz. HLA-DR, HLA-DP*, and *HLA-DQ*, to be downregulated in the IPM high-risk group compared to the low-risk group but with no changes in MHC class-I molecules. These MHC class-II molecules participate in the activation of CD4+ T cells as a helper for CD8+ T cells in antitumor response. The MHC class-II neoantigens may shape the tumor immunity and response to immunotherapy, indicating the ignored MHC-II neoantigens and CD4+ T cells as key factors that influence the response to immunotherapy (Alspach et al., 2019). Moreover, while the average objective response rate (ORR) of immune checkpoint inhibitor therapy has been estimated as 30%, the IPM high-risk group showed it as 70% (non-responsive mCRC) (Haslam and Prasad, 2019). This indicates that the immune response activated by effector T cells alone may not be enough to eliminate tumors; else, the average ORR of ICI therapy would not have been 30% (Haslam and Prasad, 2019). Furthermore, an experiment used algorithmic simulation prediction to find newer MHC class-II restricted antigen named mITGB1, and an MHC class-I neoantigen, mLAMA4, which can only be recognized by helper T cells and effector T cells, respectively. Their analysis indicated that cancer cells expressing mITGB1 or mLAMA4 alone could not induce an anticancer immune response, whereas their combined expression could decline tumor growth rates, probably via activating immune response (Ott et al., 2017).

Therefore, the IPM-high risk group was attributed to the disorders of MHC class-II molecules and CD4+ T cells infiltration. Further, our IPM could help predict the recurrence of mCRC, and the stratification of refractory mCRC may provide newer insights such as the activation of the MHC class-II may support treatment with ICIs and lead to better prognosis in mCRC.

Next, increasing evidence from clinical trials indicate that combined immunotherapies may enhance the response of cancer patients, as observed especially for gastrointestinal tumors that are characterized by a complex matrix, and considerable molecular and immunological differences (Wang et al., 2019). Since *CDKN2A* with an HR > 1 is a key contributor of high-risk mCRC and *CDKN2A* was confirmed as one of the characteristic markers of some immune infiltrating cells, such as exhausted CD4+ T cell (Zheng et al., 2017), regulatory T (Treg) cell (Zheng et al., 2017), and natural killer T (NKT) cell (Young et al., 2018). *CDKN2A* is highly expressed in the IPM high-risk group; we identified this kind of refractory CRLM. Besides, IPM high-risk group has more regulatory T (Treg) cell infiltration. In addition, our results indicate a high-recurrence risk of mCRC associated with its immunosuppression, which correlates with the downregulation of MHC class-II molecules, high infiltration of Treg cells, and low levels of resting memory CD4+ T cells reservation. Combined with the literature review, we could infer that exhausted CD4+ T cells might also highly infiltrate in IPM high-risk groups. Therefore, we hypotheses that an anti-CDKN2A agent along with activation of MHC class-II molecules might prevent immune status from regulatory T (Treg) cell inhibiting as well as exhausted CD4+ T cell's incapability. Thus, it could reverse the unfavorable prognosis of the IPM high-risk group, which meets an urgent clinical need in therapy design, representing the transformation value of our findings. However, this treatment modality should be thoroughly investigated for all mCRC cases to avoid recurrence in the future.

Finally, we observed the IPM be independent of conventional clinical characteristics with better clinical decision value, which enabled the construction of an interactive network using six genes and led to the identification of *SLIT2* as a hub gene. Further, we also constructed a BN graph for clinical practice. To widen the hints of this study, we found the potential upstream regulators attributing to the alteration of IPM risk score, and *MEIS1*'s methylation was identified. Former studies (Zhu et al., 2017) have reported that MEIS1 overexpression could induce non-apoptotic cell death of ccRCC cells via decreasing the levels of prosurvival regulators Survivin and *BCL-2*, and *MEIS1* attenuates *in vitro* invasion and migration of ccRCC cells with downregulated epithelial–mesenchymal transition (EMT) process. However, *MEIS1*'s function in colorectal cancer still needs further research. Additionally, MEM and its related IPM helped define malignant phenotypes of cancer cells from their intrinsic activities and the immune stromal infiltrating cells activated in the mCRC-related microenvironment.

However, our study has certain limitations. First, it is retrospective in design, and thus, the results should be further confirmed by prospective studies. Although our study had a large number of mCRC samples, the training cohort of 102 samples and two validation cohorts (N1 = 142, N2 = 56) are still a small-scale dataset. Additionally, detailed clinicopathological characteristics of each sample were not available; thus, the Fong's CRS criteria could not be utilized to confirm the accuracy of MEM and IPM in our study. In addition, the functional and mechanistic hypothesis should be conducted to support the clinical application of the IPM three genes individually and in combination.

In summary, for the first time, we identified and validated an IPM that is based on three immune genes and has independent prognostic significance especially for refractory mCRC patients, which improves the perspective of the current CRS system and reflects the overall intensity of the immune response in the mCRC microenvironment. This study is also the first to investigate the mechanism difference in clinically difficultly labeled metastasis risk level with developing MEM model. Furthermore, it highlighted for the first time the suppression of MHC class II as the main reason for non-responsiveness to immunotherapy. In addition to these, this study tried to find a potential upstream dysregulation cause of IPM genes for a widened hint for future study. Finally, this study produced preclinical evidence for inhibiting *CDKN2A* and activating MHC class II in the immunotherapy of mCRC. Last but not the least, this study can be used as a reference for understanding other cancers' predicting gene models to filter out the models with predicting performance as well as biological mechanism meaning.

## Conclusions

In summary, for the first time, our study found the post-operative recurrence of mCRC to be strongly correlated to the immune microenvironment using a high-throughput analysis. Moreover, IPM could identify subgroups of mCRC with different recurrence risks and stratify the mCRC samples sensitive to immuno-/chemotherapy with biologically explainable evidence. Furthermore, our analysis also highlights the importance of MHC class-II molecules in immunotherapy of mCRC.

## Data Availability Statement

Publicly available datasets were analyzed in this study. The data can be found in Gene Expression Omnibus (GEO: http://www.ncbi.nlm.nih.gov/geo/), The Cancer Genome Atlas (TCGA: http://tcgadata.nci.nih.gov/tcga/). GSE72968, GSE72969, GSE39582, GSE41258, GSE81558, and GSE71222 dataset was from GEO; COAD (Colon Adenocarcinoma) dataset was from TCGA.

## Ethics Statement

The studies involving human participants were reviewed and approved by Institutional Review Boards of National Cancer Center/National Clinical Research Center for Cancer/Cancer Hospital, Chinese Academy of Medical Sciences and Peking Union Medical College and the requirement. Written informed consent for participation was not required for this study in accordance with the national legislation and the institutional requirements.

## Disclosure

Preliminary results of this study were reported as posters in European Society for Medical Oncology Congress 2019 (ESMO 2019) and 2020 ASCO Annual Congress and published as an abstract in Annals of Oncology (Luo et al., 2019), with updated results in Journal of Clinical Oncology (Luo and Bi, 2020).

## Author Contributions

ZLu carried out data management and statistical analysis and specimen collection, qRT-PCR analysis, and drafted the manuscript. XC, YZ, ZH, HZ, JZha, ZLi, JZho, JL, and JC worked on data collection and specimen collection. ZLu and XB worked on polishing paper language. XC and YZ assisted on the statistical analysis. XB worked on designing and draft reviewing and performed project administration. All authors searched the literature, designed the study, interpreted the findings, and revised the manuscript.

## Conflict of Interest

The authors declare that the research was conducted in the absence of any commercial or financial relationships that could be construed as a potential conflict of interest.

## Supplementary Material

The GSEA results were attached in Supplementary Tables 1–3. The upstream transcript factor prediction results were attached in Supplementary Table 4. The BN netica file was attached.
