# Supplementary material for: Development of a Metastasis-Related Immune Prognostic Model of Metastatic Colorectal Cancer and Its Usefulness to Immunotherapy
Source: Front Cell Dev Biol. 2021 Jan 28;8:577125. doi: 10.3389/fcell.2020.577125 (PMC7876250; doi:10.3389/fcell.2020.577125)
Supplement: Supplementary file 1 [file Table_1.docx]

**Supplementary Table S1**

**Differentially expressed genes between M1 stage colorectal cancers vs. M0 stage colorectal cancers from a comprehensive analysis on 4 microarrays datasets, RobustRankAggreg methods.**

1. Up-regulation genes

| Name | Pvalue | adjPvalue | logFC |
| --- | --- | --- | --- |
| THBS2 | 5.39E-10 | 1.17E-05 | 0.381507 |
| COMP | 3.43E-09 | 7.45E-05 | 0.499949 |
| KRT23 | 5.27E-09 | 0.000114 | 0.48813 |
| SPP1 | 1.80E-08 | 0.000392 | 0.385589 |
| BMP4 | 2.01E-08 | 0.000438 | 0.355256 |
| AMIGO2 | 2.49E-08 | 0.000541 | 0.337511 |
| SPOCK1 | 4.85E-08 | 0.001054 | 0.370193 |
| RPS4Y1 | 5.09E-08 | 0.001105 | 0.494031 |
| SLC14A1 | 5.67E-08 | 0.001231 | 0.358801 |
| BAMBI | 9.25E-08 | 0.002009 | 0.343829 |
| FAP | 1.32E-07 | 0.002876 | 0.31074 |
| F13A1 | 1.36E-07 | 0.002955 | 0.274614 |
| COL10A1 | 1.72E-07 | 0.003744 | 0.41325 |
| TUBB2B | 1.72E-07 | 0.003744 | 0.328607 |
| SGCE | 2.00E-07 | 0.004353 | 0.232674 |
| DACT1 | 2.76E-07 | 0.005991 | 0.312868 |
| SLIT2 | 3.02E-07 | 0.006554 | 0.269662 |
| TMEM45A | 3.55E-07 | 0.007717 | 0.290187 |
| ASPN | 3.83E-07 | 0.008309 | 0.340527 |
| FAM198B | 4.20E-07 | 0.00912 | 0.235065 |
| CHRDL1 | 4.93E-07 | 0.010707 | 0.251258 |
| CTHRC1 | 5.12E-07 | 0.011112 | 0.254498 |
| WIF1 | 6.83E-07 | 0.014845 | 0.400424 |
| GRP | 7.39E-07 | 0.016047 | 0.360111 |
| ZNF532 | 7.90E-07 | 0.01717 | 0.248207 |
| PLN | 9.22E-07 | 0.020032 | 0.329535 |
| STMN2 | 1.21E-06 | 0.026319 | 0.321688 |
| COL16A1 | 1.22E-06 | 0.026606 | 0.200663 |
| SULF1 | 1.38E-06 | 0.029899 | 0.308693 |
| VIP | 1.38E-06 | 0.029899 | 0.332509 |
| MGP | 1.42E-06 | 0.030842 | 0.350884 |
| COL11A1 | 1.85E-06 | 0.040235 | 0.316474 |

2. Down-regulation genes

| Name | Pvalue | adjPvalue | logFC |
| --- | --- | --- | --- |
| CXCL13 | 9.54E-11 | 2.07E-06 | -0.49233 |
| PIGR | 3.37E-10 | 7.32E-06 | -0.49387 |
| PDZK1IP1 | 2.35E-09 | 5.11E-05 | -0.34291 |
| CXCL9 | 9.10E-09 | 0.000198 | -0.3421 |
| BLNK | 1.27E-08 | 0.000275 | -0.31017 |
| CXCL11 | 1.66E-08 | 0.000361 | -0.50474 |
| CXCL10 | 2.16E-08 | 0.000468 | -0.25805 |
| CXCL1 | 1.04E-07 | 0.002264 | -0.31404 |
| CASP1 | 1.52E-07 | 0.003295 | -0.26535 |
| TNFRSF11A | 1.95E-07 | 0.004231 | -0.3568 |
| OLFM4 | 2.03E-07 | 0.004419 | -0.47516 |
| ASS1 | 4.11E-07 | 0.008935 | -0.21507 |
| LCN2 | 6.06E-07 | 0.013157 | -0.42214 |
| MUC4 | 7.11E-07 | 0.015438 | -0.29455 |
| IGJ | 1.29E-06 | 0.028071 | -0.18979 |
| TK1 | 1.29E-06 | 0.028094 | -0.21459 |
| MUC5AC | 1.36E-06 | 0.029644 | -0.18331 |
| PCK1 | 1.42E-06 | 0.030842 | -0.16853 |
| CXCL3 | 1.65E-06 | 0.035855 | -0.33516 |
| GMDS | 2.24E-06 | 0.048732 | -0.27647 |
| TNFRSF17 | 2.24E-06 | 0.048732 | -0.21651 |
