# Supplementary material for: Development of a Metastasis-Related Immune Prognostic Model of Metastatic Colorectal Cancer and Its Usefulness to Immunotherapy
Source: Front Cell Dev Biol. 2021 Jan 28;8:577125. doi: 10.3389/fcell.2020.577125 (PMC7876250; doi:10.3389/fcell.2020.577125)
Supplement: Supplementary file 2 [file Table_2.docx]

**Supplementary Table S2**

1. GSEA for MEM-low recurrence cluster

| NAME | SIZE | NES | NOM p-val | FDR q-val |
| --- | --- | --- | --- | --- |
| GO_EXTRACELLULAR_STRUCTURE_ORGANIZATION | 289 | 2.468736 | 0 | 0 |
| GO_COLLAGEN_FIBRIL_ORGANIZATION | 36 | 2.365421 | 0 | 0 |
| GO_REGULATION_OF_SEQUESTERING_OF_CALCIUM_ION | 97 | 2.263108 | 0 | 7.60E-04 |
| GO_REGULATION_OF_LEUKOCYTE_CHEMOTAXIS | 90 | 2.25464 | 0 | 0.001127 |
| GO_MYD88_DEPENDENT_TOLL_LIKE_RECEPTOR_SIGNALING_PATHWAY | 32 | 2.252366 | 0 | 9.01E-04 |
| GO_POSITIVE_REGULATION_OF_TUMOR_NECROSIS_FACTOR_SUPERFAMILY_CYTOKINE_PRODUCTION | 54 | 2.174423 | 0 | 0.001125 |
| GO_REGULATION_OF_TUMOR_NECROSIS_FACTOR_SUPERFAMILY_CYTOKINE_PRODUCTION | 91 | 2.169819 | 0 | 0.001127 |
| GO_POSITIVE_REGULATION_OF_RECEPTOR_MEDIATED_ENDOCYTOSIS | 44 | 2.150454 | 0 | 0.001404 |
| GO_NEGATIVE_REGULATION_OF_BMP_SIGNALING_PATHWAY | 40 | 2.139734 | 0 | 0.001745 |
| GO_POSITIVE_REGULATION_OF_CHEMOTAXIS | 117 | 2.131485 | 0 | 0.001796 |
| GO_PHAGOCYTOSIS_ENGULFMENT | 24 | 2.121666 | 0 | 0.002147 |
| GO_SULFUR_COMPOUND_CATABOLIC_PROCESS | 38 | 2.115804 | 0 | 0.002438 |
| GO_REGULATION_OF_CARDIAC_MUSCLE_CONTRACTION_BY_REGULATION_OF_THE_RELEASE_OF_SEQUESTERED_CALCIUM_ION | 17 | 2.113554 | 0 | 0.00242 |
| GO_POSITIVE_REGULATION_OF_LEUKOCYTE_CHEMOTAXIS | 78 | 2.109342 | 0 | 0.002567 |
| GO_POSITIVE_REGULATION_OF_LOCOMOTION | 397 | 2.103073 | 0 | 0.00277 |
| GO_REGULATION_OF_MONONUCLEAR_CELL_MIGRATION | 16 | 2.098743 | 0 | 0.002736 |
| GO_REGULATION_OF_GRANULOCYTE_CHEMOTAXIS | 37 | 2.096651 | 0 | 0.002708 |
| GO_POSITIVE_REGULATION_OF_ENDOCYTOSIS | 108 | 2.094912 | 0 | 0.002557 |
| GO_REGULATION_OF_CHEMOTAXIS | 172 | 2.093454 | 0 | 0.002715 |
| GO_CELL_CHEMOTAXIS | 150 | 2.091381 | 0 | 0.002749 |
| GO_NEGATIVE_REGULATION_OF_CALCIUM_ION_TRANSMEMBRANE_TRANSPORT | 26 | 2.087994 | 0 | 0.002885 |
| GO_LEUKOCYTE_MIGRATION | 248 | 2.077783 | 0 | 0.003514 |
| GO_TOLL_LIKE_RECEPTOR_SIGNALING_PATHWAY | 83 | 2.075369 | 0 | 0.003506 |
| GO_REGULATION_OF_RELEASE_OF_SEQUESTERED_CALCIUM_ION_INTO_CYTOSOL | 68 | 2.068778 | 0 | 0.004017 |
| GO_SUBSTRATE_DEPENDENT_CELL_MIGRATION | 27 | 2.062446 | 0 | 0.004171 |
| GO_PHAGOCYTOSIS | 156 | 2.062132 | 0 | 0.004011 |
| GO_REGULATION_OF_RELEASE_OF_SEQUESTERED_CALCIUM_ION_INTO_CYTOSOL_BY_SARCOPLASMIC_RETICULUM | 23 | 2.060525 | 0 | 0.003988 |
| GO_MUSCLE_SYSTEM_PROCESS | 275 | 2.056349 | 0 | 0.004441 |
| GO_CYTOSOLIC_CALCIUM_ION_TRANSPORT | 49 | 2.055278 | 0 | 0.004288 |
| GO_POSITIVE_REGULATION_OF_LEUKOCYTE_MIGRATION | 105 | 2.046839 | 0 | 0.004594 |
| GO_CALCIUM_ION_IMPORT_INTO_CYTOSOL | 39 | 2.044913 | 0 | 0.004518 |
| GO_REGULATION_OF_CALCIUM_MEDIATED_SIGNALING | 70 | 2.044367 | 0 | 0.004412 |
| GO_POSITIVE_REGULATION_OF_RESPONSE_TO_EXTERNAL_STIMULUS | 281 | 2.04045 | 0 | 0.004418 |
| GO_REGULATION_OF_SMOOTH_MUSCLE_CELL_MIGRATION | 48 | 2.03912 | 0 | 0.004387 |
| GO_AXON_EXTENSION | 36 | 2.038863 | 0 | 0.004261 |
| GO_NEGATIVE_REGULATION_OF_CELLULAR_RESPONSE_TO_GROWTH_FACTOR_STIMULUS | 117 | 2.036886 | 0 | 0.004268 |
| GO_REGULATION_OF_OSSIFICATION | 170 | 2.035204 | 0 | 0.004275 |
| GO_REGULATION_OF_MONOCYTE_CHEMOTAXIS | 20 | 2.029237 | 0 | 0.004604 |
| GO_REGULATION_OF_PHOSPHOLIPASE_ACTIVITY | 63 | 2.028334 | 0 | 0.004515 |
| GO_REGULATION_OF_ADHERENS_JUNCTION_ORGANIZATION | 49 | 2.019474 | 0 | 0.004827 |
| GO_NEGATIVE_REGULATION_OF_CARTILAGE_DEVELOPMENT | 24 | 2.019288 | 0 | 0.004709 |
| GO_COMPLEMENT_ACTIVATION | 48 | 2.016079 | 0 | 0.00484 |
| GO_LEUKOCYTE_CHEMOTAXIS | 107 | 2.015506 | 0 | 0.004754 |
| GO_PLATELET_DEGRANULATION | 99 | 2.013053 | 0 | 0.004851 |
| GO_CALCIUM_MEDIATED_SIGNALING | 80 | 2.011741 | 0 | 0.004845 |
| GO_AMINOGLYCAN_CATABOLIC_PROCESS | 65 | 2.011318 | 0 | 0.004764 |
| GO_OSSIFICATION | 235 | 2.003397 | 0 | 0.005285 |
| GO_ACTIVATION_OF_IMMUNE_RESPONSE | 380 | 2.000011 | 0 | 0.005479 |
| GO_REGULATION_OF_BIOMINERAL_TISSUE_DEVELOPMENT | 68 | 1.997939 | 0 | 0.005526 |
| GO_REGULATION_OF_LEUKOCYTE_MIGRATION | 140 | 1.997693 | 0 | 0.005438 |
| GO_CELLULAR_RESPONSE_TO_VITAMIN | 25 | 1.996018 | 0 | 0.005575 |
| GO_MYELOID_LEUKOCYTE_MIGRATION | 90 | 1.990343 | 0 | 0.006073 |
| GO_MEMBRANE_INVAGINATION | 33 | 1.985444 | 0 | 0.00672 |
| GO_REGULATION_OF_NEUTROPHIL_CHEMOTAXIS | 25 | 1.980418 | 0 | 0.007345 |
| GO_POSITIVE_REGULATION_OF_SMOOTH_MUSCLE_CELL_MIGRATION | 29 | 1.973813 | 0 | 0.008051 |
| GO_PATTERN_RECOGNITION_RECEPTOR_SIGNALING_PATHWAY | 105 | 1.971376 | 0 | 0.008048 |
| GO_PHAGOCYTOSIS_RECOGNITION | 20 | 1.968236 | 0 | 0.008423 |
| GO_MUSCLE_CONTRACTION | 226 | 1.966919 | 0 | 0.008392 |
| GO_LYMPHOCYTE_CHEMOTAXIS | 30 | 1.959006 | 0 | 0.009224 |
| GO_NEGATIVE_REGULATION_OF_TRANSMEMBRANE_RECEPTOR_PROTEIN_SERINE_THREONINE_KINASE_SIGNALING_PATHWAY | 97 | 1.953691 | 0 | 0.009838 |
| GO_REGULATION_OF_CELL_JUNCTION_ASSEMBLY | 67 | 1.946941 | 0 | 0.010804 |
| GO_REGULATION_OF_PHOSPHOLIPASE_C_ACTIVITY | 39 | 1.946769 | 0 | 0.010647 |
| GO_NEGATIVE_REGULATION_OF_ANOIKIS | 17 | 1.946518 | 0 | 0.010478 |
| GO_REGULATION_OF_NEUTROPHIL_MIGRATION | 30 | 1.940752 | 0 | 0.011403 |
| GO_REGULATION_OF_CARDIAC_MUSCLE_CONTRACTION_BY_CALCIUM_ION_SIGNALING | 21 | 1.939548 | 0 | 0.011399 |
| GO_POSITIVE_REGULATION_OF_PHOSPHOLIPASE_ACTIVITY | 53 | 1.939008 | 0 | 0.011312 |
| GO_RESPONSE_TO_HYDROPEROXIDE | 15 | 1.933553 | 0.001984 | 0.012183 |
| GO_REGULATION_OF_CALCIUM_ION_IMPORT | 92 | 1.93209 | 0 | 0.012234 |
| GO_REGULATION_OF_ION_HOMEOSTASIS | 189 | 1.931657 | 0 | 0.012106 |
| GO_BLOOD_VESSEL_MORPHOGENESIS | 354 | 1.928996 | 0 | 0.012223 |
| GO_HEART_PROCESS | 83 | 1.928609 | 0 | 0.012162 |
| GO_REGULATION_OF_CELL_SUBSTRATE_ADHESION | 165 | 1.92731 | 0 | 0.012242 |
| GO_POSITIVE_REGULATION_OF_RECEPTOR_INTERNALIZATION | 22 | 1.922867 | 0.001946 | 0.012645 |
| GO_DERMATAN_SULFATE_PROTEOGLYCAN_METABOLIC_PROCESS | 16 | 1.921513 | 0.00188 | 0.012733 |
| GO_REGULATION_OF_CALCIUM_ION_TRANSMEMBRANE_TRANSPORT | 109 | 1.91756 | 0 | 0.013222 |
| GO_REGULATION_OF_MACROPHAGE_CHEMOTAXIS | 16 | 1.917396 | 0.005515 | 0.013122 |
| GO_REGULATION_OF_CALCIUM_ION_TRANSPORT_INTO_CYTOSOL | 85 | 1.914501 | 0 | 0.013447 |
| GO_SMOOTH_MUSCLE_CONTRACTION | 45 | 1.914214 | 0 | 0.013318 |
| GO_CELLULAR_RESPONSE_TO_INTERFERON_GAMMA | 106 | 1.911994 | 0 | 0.013691 |
| GO_CELLULAR_EXTRAVASATION | 25 | 1.910663 | 0.005803 | 0.013786 |
| GO_MYELOID_LEUKOCYTE_ACTIVATION | 93 | 1.909154 | 0 | 0.013977 |
| GO_VASCULATURE_DEVELOPMENT | 456 | 1.908816 | 0 | 0.013847 |
| GO_RESPONSE_TO_PROSTAGLANDIN | 32 | 1.907228 | 0.00188 | 0.013952 |
| GO_LYMPHOCYTE_MIGRATION | 41 | 1.905072 | 0 | 0.014163 |
| GO_REGULATION_OF_CYTOSOLIC_CALCIUM_ION_CONCENTRATION | 191 | 1.902682 | 0 | 0.014366 |
| GO_EXTRACELLULAR_MATRIX_ASSEMBLY | 16 | 1.900208 | 0.001931 | 0.014642 |
| GO_POSITIVE_REGULATION_OF_OSTEOBLAST_DIFFERENTIATION | 58 | 1.900112 | 0.001706 | 0.014526 |
| GO_REGULATION_OF_CHONDROCYTE_DIFFERENTIATION | 43 | 1.89961 | 0 | 0.014438 |
| GO_REGULATION_OF_CELL_SHAPE | 131 | 1.897744 | 0 | 0.014743 |
| GO_DEFINITIVE_HEMOPOIESIS | 15 | 1.896283 | 0.001961 | 0.014828 |
| GO_NEGATIVE_REGULATION_OF_G_PROTEIN_COUPLED_RECEPTOR_PROTEIN_SIGNALING_PATHWAY | 38 | 1.896031 | 0 | 0.014714 |
| GO_SECOND_MESSENGER_MEDIATED_SIGNALING | 149 | 1.89201 | 0 | 0.015252 |
| GO_POSITIVE_REGULATION_OF_PHOSPHATIDYLINOSITOL_3_KINASE_SIGNALING | 61 | 1.891813 | 0 | 0.015124 |
| GO_REGULATION_OF_RECEPTOR_MEDIATED_ENDOCYTOSIS | 75 | 1.889997 | 0 | 0.015416 |
| GO_REGULATION_OF_RYANODINE_SENSITIVE_CALCIUM_RELEASE_CHANNEL_ACTIVITY | 23 | 1.888195 | 0.001901 | 0.015513 |
| GO_INTEGRIN_MEDIATED_SIGNALING_PATHWAY | 80 | 1.885258 | 0 | 0.015903 |
| GO_POSITIVE_REGULATION_OF_MONOCYTE_CHEMOTAXIS | 15 | 1.879191 | 0 | 0.0171 |
| GO_PROTEIN_ACTIVATION_CASCADE | 70 | 1.874268 | 0 | 0.018094 |
| GO_G_PROTEIN_COUPLED_PURINERGIC_RECEPTOR_SIGNALING_PATHWAY | 18 | 1.872161 | 0.003868 | 0.018331 |
| GO_MULTICELLULAR_ORGANISMAL_MACROMOLECULE_METABOLIC_PROCESS | 76 | 1.872092 | 0 | 0.01817 |
| GO_HEART_DEVELOPMENT | 445 | 1.871987 | 0 | 0.018012 |
| GO_PURINERGIC_NUCLEOTIDE_RECEPTOR_SIGNALING_PATHWAY | 21 | 1.871962 | 0.001965 | 0.017836 |
| GO_POSITIVE_REGULATION_OF_OSSIFICATION | 82 | 1.871647 | 0 | 0.017706 |
| GO_RELAXATION_OF_MUSCLE | 20 | 1.871085 | 0 | 0.017622 |
| GO_RESPONSE_TO_ZINC_ION | 51 | 1.869723 | 0 | 0.017776 |
| GO_POSITIVE_REGULATION_OF_LIPID_STORAGE | 16 | 1.868381 | 0.001969 | 0.017895 |
| GO_POSITIVE_REGULATION_OF_VASCULATURE_DEVELOPMENT | 126 | 1.867065 | 0 | 0.018085 |
| GO_LEUKOCYTE_PROLIFERATION | 85 | 1.866605 | 0 | 0.018 |
| GO_REGULATION_OF_CARTILAGE_DEVELOPMENT | 59 | 1.864696 | 0 | 0.018236 |
| GO_NEGATIVE_REGULATION_OF_CHONDROCYTE_DIFFERENTIATION | 18 | 1.864242 | 0 | 0.018193 |
| GO_NEGATIVE_REGULATION_OF_LOCOMOTION | 251 | 1.864228 | 0 | 0.018029 |
| GO_RECEPTOR_INTERNALIZATION | 47 | 1.861732 | 0 | 0.018551 |
| GO_REGULATION_OF_LIPASE_ACTIVITY | 82 | 1.860122 | 0 | 0.018686 |
| GO_MULTICELLULAR_ORGANISM_METABOLIC_PROCESS | 90 | 1.859085 | 0 | 0.01879 |
| GO_REGULATION_OF_PROTEIN_AUTOPHOSPHORYLATION | 33 | 1.858851 | 0.003738 | 0.018646 |
| GO_REGULATION_OF_BMP_SIGNALING_PATHWAY | 75 | 1.856312 | 0 | 0.019116 |
| GO_ANGIOGENESIS | 283 | 1.851534 | 0 | 0.020155 |
| GO_NEGATIVE_REGULATION_OF_ADHERENS_JUNCTION_ORGANIZATION | 15 | 1.851179 | 0.002041 | 0.020031 |
| GO_PURINERGIC_RECEPTOR_SIGNALING_PATHWAY | 26 | 1.85106 | 0 | 0.01991 |
| GO_POSITIVE_REGULATION_OF_PHOSPHOLIPID_METABOLIC_PROCESS | 41 | 1.847008 | 0.001832 | 0.020605 |
| GO_REGULATION_OF_CYTOKINE_SECRETION | 136 | 1.843718 | 0 | 0.021151 |
| GO_POSITIVE_REGULATION_OF_CELL_ACTIVATION | 284 | 1.842625 | 0 | 0.021253 |
| GO_CIRCULATORY_SYSTEM_PROCESS | 354 | 1.840863 | 0 | 0.021729 |
| GO_POSITIVE_REGULATION_OF_INFLAMMATORY_RESPONSE | 104 | 1.840644 | 0 | 0.021616 |
| GO_REGULATION_OF_NON_CANONICAL_WNT_SIGNALING_PATHWAY | 19 | 1.840254 | 0 | 0.021524 |
| GO_NEGATIVE_REGULATION_OF_INTERLEUKIN_12_PRODUCTION | 15 | 1.837904 | 0.001912 | 0.021931 |
| GO_REGULATION_OF_VASCULATURE_DEVELOPMENT | 224 | 1.837028 | 0 | 0.022008 |
| GO_NEGATIVE_REGULATION_OF_CHEMOTAXIS | 46 | 1.835801 | 0 | 0.022143 |
| GO_B_CELL_RECEPTOR_SIGNALING_PATHWAY | 40 | 1.835649 | 0.003503 | 0.021998 |
| GO_NEGATIVE_REGULATION_OF_RESPONSE_TO_CYTOKINE_STIMULUS | 41 | 1.835094 | 0.001825 | 0.021993 |
| GO_TAXIS | 426 | 1.831836 | 0 | 0.022771 |
| GO_POSITIVE_REGULATION_OF_NEUTROPHIL_MIGRATION | 26 | 1.831341 | 0.005758 | 0.022768 |
| GO_NEURON_PROJECTION_EXTENSION | 51 | 1.830145 | 0 | 0.022968 |
| GO_REGULATION_OF_CARDIAC_MUSCLE_CELL_CONTRACTION | 27 | 1.828241 | 0.001815 | 0.023292 |
| GO_REGULATION_OF_ACTIN_FILAMENT_BASED_MOVEMENT | 32 | 1.828009 | 0.001876 | 0.023178 |
| GO_CENTRAL_NERVOUS_SYSTEM_PROJECTION_NEURON_AXONOGENESIS | 22 | 1.825951 | 0 | 0.023538 |
| GO_CALCIUM_ION_IMPORT | 60 | 1.825369 | 0 | 0.023547 |
| GO_NEGATIVE_REGULATION_OF_REACTIVE_OXYGEN_SPECIES_BIOSYNTHETIC_PROCESS | 15 | 1.825011 | 0.002037 | 0.02345 |
| GO_REGULATION_OF_MUSCLE_CONTRACTION | 143 | 1.824077 | 0 | 0.023573 |
| GO_REGULATION_OF_CALCIUM_ION_TRANSPORT | 197 | 1.822989 | 0 | 0.023726 |
| GO_ENDODERMAL_CELL_DIFFERENTIATION | 38 | 1.822091 | 0.003515 | 0.023765 |
| GO_NEGATIVE_REGULATION_OF_TRANSPORTER_ACTIVITY | 60 | 1.819833 | 0 | 0.0242 |
| GO_NEGATIVE_REGULATION_OF_CALCIUM_MEDIATED_SIGNALING | 15 | 1.819424 | 0.002101 | 0.024172 |
| GO_REGULATION_OF_RECEPTOR_INTERNALIZATION | 35 | 1.819039 | 0.006932 | 0.024121 |
| GO_BONE_GROWTH | 19 | 1.818821 | 0.007874 | 0.024056 |
| GO_AMINOGLYCAN_BIOSYNTHETIC_PROCESS | 105 | 1.818228 | 0 | 0.024084 |
| GO_INFLAMMATORY_RESPONSE | 419 | 1.817715 | 0 | 0.024064 |
| GO_GRANULOCYTE_MIGRATION | 66 | 1.817354 | 0.001672 | 0.024 |
| GO_DENDRITIC_CELL_MIGRATION | 21 | 1.816699 | 0.003584 | 0.024065 |
| GO_REGULATION_OF_MITOCHONDRIAL_FISSION | 15 | 1.813732 | 0.004 | 0.024737 |
| GO_RESPONSE_TO_AMINO_ACID | 106 | 1.811903 | 0 | 0.025182 |
| GO_HUMORAL_IMMUNE_RESPONSE_MEDIATED_BY_CIRCULATING_IMMUNOGLOBULIN | 38 | 1.809699 | 0.005607 | 0.025658 |
| GO_POSITIVE_REGULATION_OF_ERK1_AND_ERK2_CASCADE | 155 | 1.808854 | 0 | 0.02569 |
| GO_REGULATION_OF_ENDOCYTOSIS | 190 | 1.807954 | 0 | 0.025763 |
| GO_REGULATION_OF_ANOIKIS | 24 | 1.807189 | 0.001949 | 0.0258 |
| GO_REGULATION_OF_ENDOTHELIAL_CELL_PROLIFERATION | 94 | 1.805879 | 0.001656 | 0.025981 |
| GO_CARDIAC_VENTRICLE_MORPHOGENESIS | 60 | 1.805117 | 0 | 0.026087 |
| GO_POSITIVE_REGULATION_OF_PROTEIN_AUTOPHOSPHORYLATION | 20 | 1.803733 | 0.00188 | 0.02637 |
| GO_REGULATION_OF_GASTRULATION | 31 | 1.803452 | 0 | 0.026317 |
| GO_CHONDROCYTE_DEVELOPMENT | 21 | 1.803375 | 0.007533 | 0.026159 |
| GO_REGULATION_OF_CELLULAR_RESPONSE_TO_GROWTH_FACTOR_STIMULUS | 220 | 1.802323 | 0 | 0.026331 |
| GO_REGULATION_OF_CALCIUM_ION_TRANSMEMBRANE_TRANSPORTER_ACTIVITY | 66 | 1.798675 | 0 | 0.027145 |
| GO_REGULATION_OF_OSTEOBLAST_DIFFERENTIATION | 109 | 1.79818 | 0 | 0.027122 |
| GO_NEGATIVE_REGULATION_OF_CANONICAL_WNT_SIGNALING_PATHWAY | 155 | 1.798069 | 0 | 0.026998 |
| GO_NEGATIVE_REGULATION_OF_ENDOTHELIAL_CELL_PROLIFERATION | 30 | 1.796341 | 0.001873 | 0.027333 |
| GO_DEVELOPMENTAL_GROWTH_INVOLVED_IN_MORPHOGENESIS | 101 | 1.796168 | 0 | 0.027209 |
| GO_RENAL_SYSTEM_VASCULATURE_DEVELOPMENT | 19 | 1.795513 | 0.001905 | 0.027295 |
| GO_CHEMOKINE_MEDIATED_SIGNALING_PATHWAY | 60 | 1.792204 | 0 | 0.028204 |
| GO_REGULATION_OF_PLASMA_LIPOPROTEIN_PARTICLE_LEVELS | 45 | 1.791411 | 0 | 0.028296 |
| GO_T_CELL_PROLIFERATION | 35 | 1.789926 | 0.001773 | 0.02854 |
| GO_NEGATIVE_REGULATION_OF_BIOMINERAL_TISSUE_DEVELOPMENT | 17 | 1.788877 | 0.003883 | 0.02874 |
| GO_MUSCLE_STRUCTURE_DEVELOPMENT | 410 | 1.787871 | 0 | 0.02888 |
| GO_RESPONSE_TO_INTERFERON_GAMMA | 127 | 1.786924 | 0 | 0.028967 |
| GO_REGULATION_OF_OSTEOBLAST_PROLIFERATION | 22 | 1.783903 | 0.005505 | 0.029827 |
| GO_RESPONSE_TO_ACIDIC_PH | 16 | 1.781246 | 0.001988 | 0.030544 |
| GO_FOREBRAIN_CELL_MIGRATION | 61 | 1.780904 | 0 | 0.030447 |
| GO_ACTIVATION_OF_PHOSPHOLIPASE_C_ACTIVITY | 27 | 1.780328 | 0.005629 | 0.030471 |
| GO_POSITIVE_REGULATION_OF_RESPONSE_TO_WOUNDING | 151 | 1.779743 | 0 | 0.030427 |
| GO_POSITIVE_REGULATION_OF_CALCIUM_ION_IMPORT | 47 | 1.779455 | 0 | 0.030338 |
| GO_POSITIVE_REGULATION_OF_LIPASE_ACTIVITY | 66 | 1.779415 | 0 | 0.030182 |
| GO_POSITIVE_REGULATION_OF_CALCIUM_ION_TRANSPORT | 99 | 1.777074 | 0 | 0.030675 |
| GO_RESPONSE_TO_MONOAMINE | 35 | 1.776161 | 0 | 0.03085 |
| GO_FIBRIL_ORGANIZATION | 18 | 1.775276 | 0.00381 | 0.030958 |
| GO_CENTRAL_NERVOUS_SYSTEM_NEURON_AXONOGENESIS | 27 | 1.773483 | 0.003968 | 0.031406 |
| GO_REGULATION_OF_LEUKOCYTE_APOPTOTIC_PROCESS | 74 | 1.77337 | 0 | 0.031273 |
| GO_NEGATIVE_REGULATION_OF_SMOOTH_MUSCLE_CELL_PROLIFERATION | 35 | 1.768346 | 0.001818 | 0.032864 |
| GO_CELLULAR_RESPONSE_TO_ACID_CHEMICAL | 160 | 1.765513 | 0 | 0.033664 |
| GO_REGULATION_OF_MACROPHAGE_DERIVED_FOAM_CELL_DIFFERENTIATION | 27 | 1.765347 | 0.007233 | 0.033539 |
| GO_CALCIUM_ION_TRANSPORT | 199 | 1.76275 | 0 | 0.03423 |
| GO_CELLULAR_RESPONSE_TO_AMINO_ACID_STIMULUS | 48 | 1.76261 | 0.005338 | 0.03411 |
| GO_RESPONSE_TO_PROSTAGLANDIN_E | 25 | 1.761134 | 0.003663 | 0.034472 |
| GO_ENDOCYTOSIS | 457 | 1.760865 | 0 | 0.034381 |
| GO_REGULATION_OF_VASCULAR_ENDOTHELIAL_GROWTH_FACTOR_PRODUCTION | 30 | 1.760294 | 0.003824 | 0.034418 |
| GO_RECEPTOR_MEDIATED_ENDOCYTOSIS | 201 | 1.75995 | 0 | 0.034363 |
| GO_IMMUNE_RESPONSE_REGULATING_CELL_SURFACE_RECEPTOR_SIGNALING_PATHWAY | 281 | 1.759528 | 0 | 0.03432 |
| GO_DEVELOPMENTAL_CELL_GROWTH | 74 | 1.75711 | 0.001802 | 0.035091 |
| GO_PLASMA_LIPOPROTEIN_PARTICLE_CLEARANCE | 21 | 1.756282 | 0.003984 | 0.035221 |
| GO_ADRENERGIC_RECEPTOR_SIGNALING_PATHWAY | 18 | 1.755834 | 0.005848 | 0.035219 |
| GO_NEGATIVE_REGULATION_OF_CELL_ADHESION | 212 | 1.754095 | 0 | 0.035697 |
| GO_REGULATION_OF_LYMPHOCYTE_MIGRATION | 33 | 1.753599 | 0.007394 | 0.035715 |
| GO_REGULATION_OF_CAMP_METABOLIC_PROCESS | 122 | 1.752901 | 0 | 0.035795 |
| GO_LIPID_TRANSLOCATION | 21 | 1.752751 | 0.003817 | 0.03569 |
| GO_KERATAN_SULFATE_METABOLIC_PROCESS | 32 | 1.752601 | 0.00354 | 0.035558 |
| GO_NEGATIVE_REGULATION_OF_SYNAPTIC_TRANSMISSION | 59 | 1.750289 | 0.001739 | 0.036316 |
| GO_REGULATION_OF_CYCLIC_NUCLEOTIDE_METABOLIC_PROCESS | 147 | 1.749498 | 0 | 0.036495 |
| GO_REGULATION_OF_PROTEIN_KINASE_A_SIGNALING | 17 | 1.746902 | 0.00998 | 0.037347 |
| GO_NEGATIVE_REGULATION_OF_CALCIUM_ION_IMPORT | 20 | 1.746829 | 0.011811 | 0.037188 |
| GO_REGULATION_OF_INFLAMMATORY_RESPONSE | 271 | 1.746687 | 0 | 0.037052 |
| GO_PROTEIN_TRIMERIZATION | 38 | 1.746465 | 0.010676 | 0.03694 |
| GO_CELLULAR_RESPONSE_TO_PROSTAGLANDIN_STIMULUS | 23 | 1.746387 | 0.007634 | 0.036769 |
| GO_DIVALENT_INORGANIC_CATION_HOMEOSTASIS | 322 | 1.745837 | 0 | 0.036814 |
| GO_CARDIAC_MUSCLE_TISSUE_DEVELOPMENT | 134 | 1.74573 | 0 | 0.036683 |
| GO_MACROPHAGE_ACTIVATION | 31 | 1.744363 | 0.003503 | 0.03705 |
| GO_POSITIVE_REGULATION_OF_DEFENSE_RESPONSE | 341 | 1.741587 | 0 | 0.03788 |
| GO_POSITIVE_REGULATION_OF_CELL_ADHESION | 359 | 1.740204 | 0 | 0.038274 |
| GO_NEGATIVE_REGULATION_OF_LEUKOCYTE_MIGRATION | 28 | 1.740124 | 0.005338 | 0.038127 |
| GO_CELL_AGGREGATION | 19 | 1.739674 | 0.008032 | 0.038117 |
| GO_POSITIVE_REGULATION_OF_CELL_ADHESION_MEDIATED_BY_INTEGRIN | 17 | 1.739637 | 0.006024 | 0.037958 |
| GO_POSITIVE_REGULATION_OF_FIBROBLAST_PROLIFERATION | 51 | 1.737623 | 0.006969 | 0.038509 |
| GO_GLOMERULUS_DEVELOPMENT | 49 | 1.737567 | 0.003636 | 0.038339 |
| GO_REGULATION_OF_EPITHELIAL_TO_MESENCHYMAL_TRANSITION | 65 | 1.736945 | 0 | 0.038389 |
| GO_NEGATIVE_REGULATION_OF_SMOOTH_MUSCLE_CELL_MIGRATION | 16 | 1.736574 | 0.008032 | 0.038378 |
| GO_STEM_CELL_DIFFERENTIATION | 186 | 1.735665 | 0 | 0.038554 |
| GO_NEGATIVE_REGULATION_OF_MUSCLE_CONTRACTION | 21 | 1.734687 | 0.007737 | 0.038782 |
| GO_NEGATIVE_REGULATION_OF_INTRACELLULAR_TRANSPORT | 130 | 1.734437 | 0 | 0.038685 |
| GO_REGULATION_OF_CHEMOKINE_PRODUCTION | 62 | 1.733729 | 0 | 0.038798 |
| GO_KERATAN_SULFATE_BIOSYNTHETIC_PROCESS | 28 | 1.731832 | 0.007634 | 0.039485 |
| GO_POSITIVE_CHEMOTAXIS | 30 | 1.730357 | 0.006873 | 0.039904 |
| GO_TISSUE_MIGRATION | 80 | 1.729797 | 0 | 0.039921 |
| GO_ENDOTHELIAL_CELL_MIGRATION | 54 | 1.729625 | 0.001704 | 0.039825 |
| GO_NEGATIVE_REGULATION_OF_EPITHELIAL_CELL_MIGRATION | 51 | 1.729594 | 0.003478 | 0.039657 |
| GO_DENDRITIC_CELL_CHEMOTAXIS | 16 | 1.729235 | 0.009653 | 0.039637 |
| GO_CELL_SUBSTRATE_ADHESION | 152 | 1.728198 | 0 | 0.039859 |
| GO_NEURON_PROJECTION_MORPHOGENESIS | 377 | 1.72705 | 0 | 0.040112 |
| GO_POSITIVE_REGULATION_OF_GLYCOPROTEIN_METABOLIC_PROCESS | 17 | 1.726347 | 0.003854 | 0.040209 |
| GO_REGULATION_OF_SMOOTH_MUSCLE_CONTRACTION | 59 | 1.726124 | 0.003478 | 0.040096 |
| GO_REGULATION_OF_CELL_MORPHOGENESIS_INVOLVED_IN_DIFFERENTIATION | 324 | 1.725814 | 0 | 0.040022 |
| GO_REGULATION_OF_ERK1_AND_ERK2_CASCADE | 219 | 1.725369 | 0 | 0.040061 |
| GO_MESENCHYME_DEVELOPMENT | 180 | 1.724603 | 0 | 0.040166 |
| GO_CELLULAR_RESPONSE_TO_CARBOHYDRATE_STIMULUS | 72 | 1.723631 | 0.00173 | 0.040435 |
| GO_REGULATION_OF_PROTEIN_IMPORT | 173 | 1.722757 | 0.001458 | 0.040631 |
| GO_RECEPTOR_METABOLIC_PROCESS | 77 | 1.721079 | 0.005059 | 0.041095 |
| GO_REGULATION_OF_ACTIN_FILAMENT_BASED_PROCESS | 300 | 1.72082 | 0 | 0.041037 |
| GO_REGULATION_OF_T_HELPER_1_TYPE_IMMUNE_RESPONSE | 22 | 1.720423 | 0.005747 | 0.041044 |
| GO_REGULATION_OF_MUSCLE_SYSTEM_PROCESS | 190 | 1.720375 | 0 | 0.040909 |
| GO_REGULATION_OF_RESPONSE_TO_WOUNDING | 388 | 1.720095 | 0 | 0.04083 |
| GO_POSITIVE_REGULATION_OF_CELL_SUBSTRATE_ADHESION | 96 | 1.719218 | 0.001626 | 0.040997 |
| GO_REGULATION_OF_CELL_ACTIVATION | 448 | 1.719157 | 0 | 0.04085 |
| GO_NEGATIVE_REGULATION_OF_TUMOR_NECROSIS_FACTOR_SUPERFAMILY_CYTOKINE_PRODUCTION | 37 | 1.718424 | 0.007505 | 0.041003 |
| GO_REGULATION_OF_INTERLEUKIN_12_PRODUCTION | 51 | 1.717339 | 0.001727 | 0.041266 |
| GO_REGULATION_OF_PEPTIDYL_TYROSINE_PHOSPHORYLATION | 202 | 1.717078 | 0 | 0.041232 |
| GO_NEGATIVE_REGULATION_OF_CELL_SUBSTRATE_ADHESION | 49 | 1.716511 | 0.001789 | 0.041305 |
| GO_RESPIRATORY_BURST | 15 | 1.715828 | 0.011673 | 0.041444 |
| GO_REGULATION_OF_FAT_CELL_DIFFERENTIATION | 96 | 1.71177 | 0 | 0.042875 |
| GO_REGULATION_OF_LEUKOCYTE_PROLIFERATION | 198 | 1.71111 | 0 | 0.043034 |
| GO_MAST_CELL_ACTIVATION | 21 | 1.71084 | 0.007874 | 0.042994 |
| GO_CELLULAR_RESPONSE_TO_CAMP | 48 | 1.708989 | 0.005217 | 0.043558 |
| GO_NEGATIVE_REGULATION_OF_ION_TRANSPORT | 119 | 1.708659 | 0.001647 | 0.043533 |
| GO_NEGATIVE_REGULATION_OF_ANION_TRANSPORT | 31 | 1.708364 | 0.001883 | 0.0435 |
| GO_CELL_MORPHOGENESIS_INVOLVED_IN_DIFFERENTIATION | 492 | 1.705237 | 0 | 0.044773 |
| GO_NEGATIVE_REGULATION_OF_IMMUNE_SYSTEM_PROCESS | 343 | 1.704024 | 0 | 0.04514 |
| GO_POSITIVE_REGULATION_OF_FILOPODIUM_ASSEMBLY | 24 | 1.703933 | 0.007797 | 0.044985 |
| GO_NEGATIVE_REGULATION_OF_CELL_JUNCTION_ASSEMBLY | 19 | 1.703718 | 0.014 | 0.044916 |
| GO_NEGATIVE_REGULATION_OF_LEUKOCYTE_APOPTOTIC_PROCESS | 42 | 1.702781 | 0.013384 | 0.045092 |
| GO_NEURON_RECOGNITION | 33 | 1.702637 | 0.003846 | 0.045007 |
| GO_POSITIVE_REGULATION_OF_PHAGOCYTOSIS | 44 | 1.70248 | 0.001842 | 0.044956 |
| GO_MUSCLE_CELL_MIGRATION | 17 | 1.700279 | 0.013011 | 0.045762 |
| GO_CARTILAGE_DEVELOPMENT | 140 | 1.700261 | 0 | 0.045599 |
| GO_APOPTOTIC_CELL_CLEARANCE | 26 | 1.699911 | 0.001938 | 0.045589 |
| GO_POSITIVE_REGULATION_OF_RELEASE_OF_SEQUESTERED_CALCIUM_ION_INTO_CYTOSOL | 34 | 1.699493 | 0.008865 | 0.045632 |
| GO_REGULATION_OF_SKELETAL_MUSCLE_CELL_DIFFERENTIATION | 15 | 1.698667 | 0.00789 | 0.045804 |
| GO_NEGATIVE_REGULATION_OF_CELL_PROJECTION_ORGANIZATION | 137 | 1.697802 | 0.001585 | 0.045954 |
| GO_CONNECTIVE_TISSUE_DEVELOPMENT | 186 | 1.697684 | 0 | 0.045835 |
| GO_B_CELL_MEDIATED_IMMUNITY | 68 | 1.696553 | 0.004992 | 0.046201 |
| GO_REGULATION_OF_CARDIAC_MUSCLE_CONTRACTION | 63 | 1.696333 | 0 | 0.046127 |
| GO_CELL_COMMUNICATION_INVOLVED_IN_CARDIAC_CONDUCTION | 36 | 1.695929 | 0.005671 | 0.046204 |
| GO_REGULATION_OF_GLYCOPROTEIN_METABOLIC_PROCESS | 39 | 1.694647 | 0.005736 | 0.04654 |
| GO_REGULATION_OF_CATION_CHANNEL_ACTIVITY | 84 | 1.692369 | 0.001618 | 0.047388 |
| GO_CARDIAC_MUSCLE_CELL_ACTION_POTENTIAL | 37 | 1.691792 | 0.003636 | 0.047513 |
| GO_REGULATION_OF_EPITHELIAL_CELL_PROLIFERATION | 274 | 1.691546 | 0 | 0.047439 |
| GO_POSITIVE_REGULATION_OF_FAT_CELL_DIFFERENTIATION | 47 | 1.690863 | 0.005208 | 0.047598 |
| GO_REGULATION_OF_G_PROTEIN_COUPLED_RECEPTOR_PROTEIN_SIGNALING_PATHWAY | 118 | 1.690063 | 0 | 0.047777 |
| GO_NEGATIVE_REGULATION_OF_CATION_TRANSMEMBRANE_TRANSPORT | 58 | 1.689661 | 0.008961 | 0.047796 |
| GO_MELANOCYTE_DIFFERENTIATION | 19 | 1.688022 | 0.013208 | 0.048307 |
| GO_NEGATIVE_REGULATION_OF_SMOOTH_MUSCLE_CONTRACTION | 15 | 1.688021 | 0.007813 | 0.048137 |
| GO_CELL_MATRIX_ADHESION | 107 | 1.687016 | 0 | 0.04839 |
| GO_MUSCLE_ORGAN_DEVELOPMENT | 259 | 1.686954 | 0 | 0.048257 |
| GO_CALCIUM_DEPENDENT_CELL_CELL_ADHESION_VIA_PLASMA_MEMBRANE_CELL_ADHESION_MOLECULES | 23 | 1.686503 | 0.014337 | 0.04828 |
| GO_POSITIVE_REGULATION_OF_ENDOTHELIAL_CELL_PROLIFERATION | 66 | 1.68612 | 0 | 0.048321 |
| GO_RESPONSE_TO_MOLECULE_OF_BACTERIAL_ORIGIN | 307 | 1.686029 | 0 | 0.048193 |
| GO_RESPONSE_TO_KETONE | 174 | 1.684632 | 0.001508 | 0.048642 |
| GO_CHONDROITIN_SULFATE_PROTEOGLYCAN_BIOSYNTHETIC_PROCESS | 29 | 1.681515 | 0.013084 | 0.049996 |

2. GSEA analysis for MEM-high risk cluster

| NAME | SIZE | NES | NOM p-val | FDR q-val |
| --- | --- | --- | --- | --- |
| GO_MITOTIC_SISTER_CHROMATID_SEGREGATION | 85 | -2.41429 | 0 | 0 |
| GO_SISTER_CHROMATID_SEGREGATION | 164 | -2.3981 | 0 | 0 |
| GO_CHROMOSOME_SEGREGATION | 255 | -2.31073 | 0 | 0 |
| GO_NUCLEAR_CHROMOSOME_SEGREGATION | 212 | -2.27547 | 0 | 0 |
| GO_ORGAN_OR_TISSUE_SPECIFIC_IMMUNE_RESPONSE | 24 | -2.27475 | 0 | 0 |
| GO_SISTER_CHROMATID_COHESION | 104 | -2.21663 | 0 | 1.33E-04 |
| GO_REGULATION_OF_TRANSCRIPTION_INVOLVED_IN_G1_S_TRANSITION_OF_MITOTIC_CELL_CYCLE | 25 | -2.20642 | 0 | 1.14E-04 |
| GO_GLUTAMINE_FAMILY_AMINO_ACID_CATABOLIC_PROCESS | 23 | -2.14478 | 0 | 9.32E-04 |
| GO_ANTIMICROBIAL_HUMORAL_RESPONSE | 41 | -2.11618 | 0 | 0.00241 |
| GO_SPERM_EGG_RECOGNITION | 38 | -2.10113 | 0 | 0.002422 |
| GO_MITOTIC_NUCLEAR_DIVISION | 336 | -2.09244 | 0 | 0.002422 |
| GO_CENTROMERE_COMPLEX_ASSEMBLY | 37 | -2.08281 | 0 | 0.002427 |
| GO_CHROMOSOME_CONDENSATION | 29 | -2.06858 | 0 | 0.003003 |
| GO_BINDING_OF_SPERM_TO_ZONA_PELLUCIDA | 30 | -2.06125 | 0 | 0.003026 |
| GO_NEGATIVE_REGULATION_OF_EPIDERMIS_DEVELOPMENT | 16 | -2.01402 | 0 | 0.006263 |
| GO_TELOMERE_MAINTENANCE_VIA_RECOMBINATION | 32 | -2.00722 | 0 | 0.00665 |
| GO_O_GLYCAN_PROCESSING | 54 | -2.00263 | 0 | 0.006553 |
| GO_HISTONE_EXCHANGE | 42 | -2.00089 | 0 | 0.006371 |
| GO_DNA_STRAND_ELONGATION | 30 | -1.99409 | 0 | 0.006782 |
| GO_DNA_DEPENDENT_DNA_REPLICATION | 90 | -1.99377 | 0 | 0.006569 |
| GO_REGULATION_OF_CYTOKINESIS | 56 | -1.98174 | 0 | 0.007158 |
| GO_NUCLEOSIDE_BISPHOSPHATE_METABOLIC_PROCESS | 35 | -1.97602 | 0.002237 | 0.007621 |
| GO_REGULATION_OF_DNA_DEPENDENT_DNA_REPLICATION | 40 | -1.9744 | 0 | 0.007508 |
| GO_STRAND_DISPLACEMENT | 26 | -1.9652 | 0 | 0.008269 |
| GO_ANAPHASE_PROMOTING_COMPLEX_DEPENDENT_CATABOLIC_PROCESS | 72 | -1.96452 | 0 | 0.00824 |
| GO_NEGATIVE_REGULATION_OF_GLIAL_CELL_DIFFERENTIATION | 26 | -1.96305 | 0 | 0.008081 |
| GO_INNATE_IMMUNE_RESPONSE_IN_MUCOSA | 17 | -1.95805 | 0 | 0.008548 |
| GO_ORGANELLE_FISSION | 459 | -1.95716 | 0 | 0.008273 |
| GO_REGULATION_OF_GLIAL_CELL_DIFFERENTIATION | 58 | -1.95267 | 0 | 0.008499 |
| GO_CELL_CYCLE_G1_S_PHASE_TRANSITION | 107 | -1.94854 | 0 | 0.008712 |
| GO_DNA_STRAND_ELONGATION_INVOLVED_IN_DNA_REPLICATION | 25 | -1.93407 | 0 | 0.011334 |
| GO_MAINTENANCE_OF_GASTROINTESTINAL_EPITHELIUM | 15 | -1.93085 | 0 | 0.011552 |
| GO_DNA_REPLICATION_INDEPENDENT_NUCLEOSOME_ORGANIZATION | 41 | -1.91176 | 0 | 0.015097 |
| GO_MEIOTIC_CELL_CYCLE | 173 | -1.88997 | 0 | 0.019665 |
| GO_MITOTIC_RECOMBINATION | 41 | -1.88954 | 0 | 0.019221 |
| GO_DNA_REPLICATION | 192 | -1.88852 | 0 | 0.018848 |
| GO_METAPHASE_PLATE_CONGRESSION | 39 | -1.88473 | 0 | 0.019373 |
| GO_SPINDLE_LOCALIZATION | 37 | -1.87853 | 0.002227 | 0.020331 |
| GO_REGULATION_OF_TRANSCRIPTION_INVOLVED_IN_CELL_FATE_COMMITMENT | 19 | -1.87469 | 0 | 0.020982 |
| GO_CELL_DIVISION | 426 | -1.86657 | 0 | 0.022909 |
| GO_REGULATION_OF_KERATINOCYTE_DIFFERENTIATION | 26 | -1.8641 | 0.00211 | 0.022875 |
| GO_REGULATION_OF_WATER_LOSS_VIA_SKIN | 16 | -1.85711 | 0 | 0.024248 |
| GO_DNA_CONFORMATION_CHANGE | 232 | -1.85675 | 0 | 0.023841 |
| GO_EPITHELIAL_STRUCTURE_MAINTENANCE | 21 | -1.85597 | 0.002119 | 0.023564 |
| GO_PURINE_RIBONUCLEOSIDE_BISPHOSPHATE_METABOLIC_PROCESS | 18 | -1.85561 | 0.001946 | 0.023095 |
| GO_DETECTION_OF_LIGHT_STIMULUS | 55 | -1.83332 | 0 | 0.029105 |
| GO_DNA_PACKAGING | 156 | -1.82848 | 0 | 0.030242 |
| GO_CHROMOSOME_LOCALIZATION | 58 | -1.82843 | 0 | 0.029612 |
| GO_ESTABLISHMENT_OF_MITOTIC_SPINDLE_LOCALIZATION | 23 | -1.82198 | 0 | 0.031112 |
| GO_POSITIVE_REGULATION_OF_CYTOKINESIS | 31 | -1.81528 | 0.002232 | 0.033118 |
| GO_ONE_CARBON_METABOLIC_PROCESS | 34 | -1.80849 | 0 | 0.035451 |
| GO_CALCIUM_ION_REGULATED_EXOCYTOSIS_OF_NEUROTRANSMITTER | 30 | -1.80715 | 0 | 0.035279 |
| GO_DNA_SYNTHESIS_INVOLVED_IN_DNA_REPAIR | 71 | -1.80339 | 0 | 0.03612 |
| GO_NEGATIVE_REGULATION_OF_CELL_DIVISION | 59 | -1.79412 | 0 | 0.039524 |
| GO_ATP_DEPENDENT_CHROMATIN_REMODELING | 63 | -1.79285 | 0.004988 | 0.039408 |
| GO_DIGESTION | 116 | -1.78869 | 0 | 0.040487 |
| GO_DNA_REPLICATION_INITIATION | 28 | -1.77526 | 0.002283 | 0.046773 |
| GO_DNA_BIOSYNTHETIC_PROCESS | 111 | -1.76927 | 0 | 0.049069 |
