# Supplementary material for: Development of a Metastasis-Related Immune Prognostic Model of Metastatic Colorectal Cancer and Its Usefulness to Immunotherapy
Source: Front Cell Dev Biol. 2021 Jan 28;8:577125. doi: 10.3389/fcell.2020.577125 (PMC7876250; doi:10.3389/fcell.2020.577125)
Supplement: Supplementary file 3 [file Table_3.docx]

**Supplementary Table S3**

1. GSEA for IPM-low recurrence group

| NAME | SIZE | NES | NOM p-val | FDR q-val |
| --- | --- | --- | --- | --- |
| GO_PROTEIN_ACTIVATION_CASCADE | 70 | 2.812507 | 0 | 0 |
| GO_CELL_CHEMOTAXIS | 150 | 2.775132 | 0 | 0 |
| GO_MYELOID_LEUKOCYTE_MIGRATION | 90 | 2.719622 | 0 | 0 |
| GO_COMPLEMENT_ACTIVATION | 48 | 2.701594 | 0 | 0 |
| GO_POSITIVE_REGULATION_OF_INFLAMMATORY_RESPONSE | 104 | 2.679423 | 0 | 0 |
| GO_REGULATION_OF_INFLAMMATORY_RESPONSE | 271 | 2.663235 | 0 | 0 |
| GO_LEUKOCYTE_CHEMOTAXIS | 107 | 2.659943 | 0 | 0 |
| GO_EXTRACELLULAR_STRUCTURE_ORGANIZATION | 289 | 2.649954 | 0 | 0 |
| GO_POSITIVE_REGULATION_OF_RESPONSE_TO_WOUNDING | 151 | 2.644625 | 0 | 0 |
| GO_REGULATION_OF_RESPONSE_TO_WOUNDING | 388 | 2.625597 | 0 | 0 |
| GO_LEUKOCYTE_MIGRATION | 248 | 2.623498 | 0 | 0 |
| GO_REGULATION_OF_ACUTE_INFLAMMATORY_RESPONSE | 67 | 2.592397 | 0 | 0 |
| GO_PLATELET_DEGRANULATION | 99 | 2.580753 | 0 | 0 |
| GO_POSITIVE_REGULATION_OF_RESPONSE_TO_EXTERNAL_STIMULUS | 281 | 2.574501 | 0 | 0 |
| GO_B_CELL_MEDIATED_IMMUNITY | 68 | 2.549686 | 0 | 0 |
| GO_REGULATION_OF_LEUKOCYTE_CHEMOTAXIS | 90 | 2.547807 | 0 | 0 |
| GO_HUMORAL_IMMUNE_RESPONSE | 144 | 2.53875 | 0 | 0 |
| GO_REGULATION_OF_CHEMOTAXIS | 172 | 2.535451 | 0 | 0 |
| GO_GRANULOCYTE_MIGRATION | 66 | 2.52845 | 0 | 0 |
| GO_POSITIVE_REGULATION_OF_ENDOCYTOSIS | 108 | 2.52816 | 0 | 0 |
| GO_POSITIVE_REGULATION_OF_CHEMOTAXIS | 117 | 2.527448 | 0 | 0 |
| GO_ADAPTIVE_IMMUNE_RESPONSE_BASED_ON_SOMATIC_RECOMBINATION_OF_IMMUNE_RECEPTORS_BUILT_FROM_IMMUNOGLOBULIN_SUPERFAMILY_DOMAINS | 123 | 2.508486 | 0 | 0 |
| GO_REGULATION_OF_VASCULATURE_DEVELOPMENT | 224 | 2.507812 | 0 | 0 |
| GO_POSITIVE_REGULATION_OF_VASCULATURE_DEVELOPMENT | 126 | 2.502101 | 0 | 0 |
| GO_INFLAMMATORY_RESPONSE | 419 | 2.501105 | 0 | 0 |
| GO_HUMORAL_IMMUNE_RESPONSE_MEDIATED_BY_CIRCULATING_IMMUNOGLOBULIN | 38 | 2.496486 | 0 | 0 |
| GO_ADAPTIVE_IMMUNE_RESPONSE | 246 | 2.482664 | 0 | 0 |
| GO_TAXIS | 426 | 2.4628 | 0 | 0 |
| GO_REGULATION_OF_LIPASE_ACTIVITY | 82 | 2.451387 | 0 | 0 |
| GO_POSITIVE_REGULATION_OF_LOCOMOTION | 397 | 2.450003 | 0 | 0 |
| GO_POSITIVE_REGULATION_OF_CELL_ACTIVATION | 284 | 2.447153 | 0 | 0 |
| GO_REGULATION_OF_NEUTROPHIL_CHEMOTAXIS | 25 | 2.435726 | 0 | 0 |
| GO_POSITIVE_REGULATION_OF_ERK1_AND_ERK2_CASCADE | 155 | 2.434969 | 0 | 0 |
| GO_REGULATION_OF_TUMOR_NECROSIS_FACTOR_SUPERFAMILY_CYTOKINE_PRODUCTION | 91 | 2.429317 | 0 | 0 |
| GO_REGULATION_OF_ENDOTHELIAL_CELL_PROLIFERATION | 94 | 2.428696 | 0 | 0 |
| GO_ACTIVATION_OF_IMMUNE_RESPONSE | 380 | 2.424125 | 0 | 0 |
| GO_POSITIVE_REGULATION_OF_LEUKOCYTE_CHEMOTAXIS | 78 | 2.411384 | 0 | 0 |
| GO_REGULATION_OF_PROTEIN_ACTIVATION_CASCADE | 31 | 2.399387 | 0 | 0 |
| GO_ANGIOGENESIS | 283 | 2.39825 | 0 | 0 |
| GO_LEUKOCYTE_MEDIATED_IMMUNITY | 150 | 2.379938 | 0 | 0 |
| GO_REGULATION_OF_PHOSPHOLIPASE_ACTIVITY | 63 | 2.378889 | 0 | 0 |
| GO_REGULATION_OF_PLASMA_LIPOPROTEIN_PARTICLE_LEVELS | 45 | 2.371396 | 0 | 0 |
| GO_REGULATION_OF_NEUTROPHIL_MIGRATION | 30 | 2.369279 | 0 | 0 |
| GO_CYTOKINE_PRODUCTION | 117 | 2.369025 | 0 | 0 |
| GO_BLOOD_VESSEL_MORPHOGENESIS | 354 | 2.364529 | 0 | 0 |
| GO_REGULATION_OF_LEUKOCYTE_MIGRATION | 140 | 2.362002 | 0 | 0 |
| GO_POSITIVE_REGULATION_OF_LEUKOCYTE_MIGRATION | 105 | 2.358323 | 0 | 0 |
| GO_POSITIVE_REGULATION_OF_PHOSPHATIDYLINOSITOL_3_KINASE_SIGNALING | 61 | 2.357454 | 0 | 0 |
| GO_REGULATION_OF_PHAGOCYTOSIS | 65 | 2.354246 | 0 | 0 |
| GO_CELLULAR_EXTRAVASATION | 25 | 2.344491 | 0 | 0 |
| GO_REGULATION_OF_GRANULOCYTE_CHEMOTAXIS | 37 | 2.339898 | 0 | 0 |
| GO_POSITIVE_REGULATION_OF_ENDOTHELIAL_CELL_PROLIFERATION | 66 | 2.338918 | 0 | 0 |
| GO_RESPONSE_TO_MOLECULE_OF_BACTERIAL_ORIGIN | 307 | 2.334208 | 0 | 0 |
| GO_POSITIVE_REGULATION_OF_PHAGOCYTOSIS | 44 | 2.330918 | 0 | 0 |
| GO_POSITIVE_REGULATION_OF_LIPASE_ACTIVITY | 66 | 2.330499 | 0 | 0 |
| GO_POSITIVE_REGULATION_OF_LIPID_METABOLIC_PROCESS | 123 | 2.325858 | 0 | 0 |
| GO_REGULATION_OF_REACTIVE_OXYGEN_SPECIES_BIOSYNTHETIC_PROCESS | 62 | 2.323613 | 0 | 0 |
| GO_REGULATION_OF_NITRIC_OXIDE_BIOSYNTHETIC_PROCESS | 50 | 2.322621 | 0 | 0 |
| GO_POSITIVE_REGULATION_OF_TUMOR_NECROSIS_FACTOR_SUPERFAMILY_CYTOKINE_PRODUCTION | 54 | 2.3168 | 0 | 0 |
| GO_POSITIVE_REGULATION_OF_CELL_ADHESION | 359 | 2.31627 | 0 | 0 |
| GO_REGULATION_OF_WOUND_HEALING | 123 | 2.310949 | 0 | 0 |
| GO_VASCULATURE_DEVELOPMENT | 456 | 2.308378 | 0 | 0 |
| GO_DENDRITIC_CELL_MIGRATION | 21 | 2.307743 | 0 | 0 |
| GO_REGULATION_OF_PHOSPHOLIPASE_C_ACTIVITY | 39 | 2.305282 | 0 | 0 |
| GO_POSITIVE_REGULATION_OF_ACUTE_INFLAMMATORY_RESPONSE | 26 | 2.303367 | 0 | 0 |
| GO_CELLULAR_RESPONSE_TO_BIOTIC_STIMULUS | 152 | 2.300929 | 0 | 0 |
| GO_NEGATIVE_REGULATION_OF_ENDOTHELIAL_CELL_MIGRATION | 37 | 2.299018 | 0 | 0 |
| GO_POSITIVE_REGULATION_OF_PHOSPHOLIPASE_ACTIVITY | 53 | 2.293571 | 0 | 0 |
| GO_POSITIVE_REGULATION_OF_CELL_SUBSTRATE_ADHESION | 96 | 2.292834 | 0 | 0 |
| GO_REGULATION_OF_CELL_ACTIVATION | 448 | 2.290749 | 0 | 0 |
| GO_REGULATION_OF_LIPID_STORAGE | 37 | 2.281253 | 0 | 1.77E-05 |
| GO_RESPONSE_TO_BACTERIUM | 462 | 2.276157 | 0 | 1.74E-05 |
| GO_POSITIVE_REGULATION_OF_EPITHELIAL_CELL_PROLIFERATION | 148 | 2.272403 | 0 | 1.72E-05 |
| GO_POSITIVE_REGULATION_OF_REACTIVE_OXYGEN_SPECIES_METABOLIC_PROCESS | 83 | 2.270735 | 0 | 1.70E-05 |
| GO_DENDRITIC_CELL_CHEMOTAXIS | 16 | 2.270706 | 0 | 1.67E-05 |
| GO_SECOND_MESSENGER_MEDIATED_SIGNALING | 149 | 2.269754 | 0 | 1.65E-05 |
| GO_LYMPHOCYTE_MEDIATED_IMMUNITY | 111 | 2.266484 | 0 | 1.63E-05 |
| GO_POSITIVE_REGULATION_OF_NEUTROPHIL_MIGRATION | 26 | 2.262686 | 0 | 1.61E-05 |
| GO_POSITIVE_REGULATION_OF_CELL_CELL_ADHESION | 229 | 2.26189 | 0 | 1.59E-05 |
| GO_CHEMOKINE_MEDIATED_SIGNALING_PATHWAY | 60 | 2.260871 | 0 | 1.57E-05 |
| GO_REGULATION_OF_COAGULATION | 85 | 2.258617 | 0 | 1.55E-05 |
| GO_POSITIVE_REGULATION_OF_B_CELL_ACTIVATION | 71 | 2.257283 | 0 | 1.53E-05 |
| GO_REGULATION_OF_ENDOCYTOSIS | 190 | 2.256386 | 0 | 1.51E-05 |
| GO_ACUTE_INFLAMMATORY_RESPONSE | 69 | 2.255072 | 0 | 1.49E-05 |
| GO_REGULATION_OF_MACROPHAGE_DERIVED_FOAM_CELL_DIFFERENTIATION | 27 | 2.253152 | 0 | 1.48E-05 |
| GO_REGULATION_OF_VASCULAR_ENDOTHELIAL_GROWTH_FACTOR_PRODUCTION | 30 | 2.24199 | 0 | 4.42E-05 |
| GO_B_CELL_RECEPTOR_SIGNALING_PATHWAY | 40 | 2.241342 | 0 | 4.37E-05 |
| GO_REGULATION_OF_ERK1_AND_ERK2_CASCADE | 219 | 2.240267 | 0 | 4.32E-05 |
| GO_NEGATIVE_REGULATION_OF_LOCOMOTION | 251 | 2.239241 | 0 | 4.27E-05 |
| GO_GLOMERULUS_DEVELOPMENT | 49 | 2.238214 | 0 | 4.22E-05 |
| GO_CELLULAR_RESPONSE_TO_INTERLEUKIN_6 | 21 | 2.229677 | 0 | 4.18E-05 |
| GO_MACROPHAGE_ACTIVATION | 31 | 2.228708 | 0 | 4.13E-05 |
| GO_INTEGRIN_MEDIATED_SIGNALING_PATHWAY | 80 | 2.223566 | 0 | 4.09E-05 |
| GO_NEGATIVE_REGULATION_OF_SMOOTH_MUSCLE_CELL_PROLIFERATION | 35 | 2.223208 | 0 | 4.04E-05 |
| GO_CIRCULATORY_SYSTEM_PROCESS | 354 | 2.222445 | 0 | 4.00E-05 |
| GO_RESPONSE_TO_INTERLEUKIN_6 | 25 | 2.220955 | 0 | 3.96E-05 |
| GO_REGULATION_OF_CALCIUM_MEDIATED_SIGNALING | 70 | 2.220627 | 0 | 3.92E-05 |
| GO_REGULATION_OF_CYTOKINE_SECRETION | 136 | 2.21893 | 0 | 5.18E-05 |
| GO_PROTEIN_LIPID_COMPLEX_SUBUNIT_ORGANIZATION | 36 | 2.211111 | 0 | 5.13E-05 |
| GO_MYELOID_LEUKOCYTE_ACTIVATION | 93 | 2.209239 | 0 | 5.07E-05 |
| GO_POSITIVE_REGULATION_OF_LEUKOCYTE_PROLIFERATION | 132 | 2.208419 | 0 | 5.02E-05 |
| GO_REGULATION_OF_HUMORAL_IMMUNE_RESPONSE | 45 | 2.206639 | 0 | 4.97E-05 |
| GO_PEPTIDE_HORMONE_PROCESSING | 30 | 2.2043 | 0 | 4.93E-05 |
| GO_REGULATION_OF_ENDOTHELIAL_CELL_MIGRATION | 110 | 2.202733 | 0 | 4.88E-05 |
| GO_NEGATIVE_REGULATION_OF_CELLULAR_RESPONSE_TO_GROWTH_FACTOR_STIMULUS | 117 | 2.197214 | 0 | 6.05E-05 |
| GO_CYTOKINE_SECRETION | 37 | 2.194382 | 0 | 6.00E-05 |
| GO_NEGATIVE_REGULATION_OF_ION_TRANSPORT | 119 | 2.192859 | 0 | 7.13E-05 |
| GO_NEGATIVE_REGULATION_OF_ENDOTHELIAL_CELL_APOPTOTIC_PROCESS | 27 | 2.187793 | 0 | 8.28E-05 |
| GO_REGULATION_OF_SMOOTH_MUSCLE_CELL_MIGRATION | 48 | 2.187401 | 0 | 8.20E-05 |
| GO_VASCULAR_PROCESS_IN_CIRCULATORY_SYSTEM | 160 | 2.186451 | 0 | 8.13E-05 |
| GO_RESPONSE_TO_PROSTAGLANDIN | 32 | 2.185453 | 0 | 8.05E-05 |
| GO_REGULATION_OF_ENDOTHELIAL_CELL_APOPTOTIC_PROCESS | 40 | 2.184681 | 0 | 7.98E-05 |
| GO_REGULATION_OF_B_CELL_ACTIVATION | 106 | 2.18423 | 0 | 7.91E-05 |
| GO_REGULATION_OF_EPITHELIAL_CELL_PROLIFERATION | 274 | 2.184041 | 0 | 7.84E-05 |
| GO_POSITIVE_REGULATION_OF_PHOSPHOLIPID_METABOLIC_PROCESS | 41 | 2.183129 | 0 | 7.77E-05 |
| GO_MONOCYTE_CHEMOTAXIS | 34 | 2.182476 | 0 | 7.71E-05 |
| GO_CHOLESTEROL_EFFLUX | 26 | 2.182422 | 0 | 7.64E-05 |
| GO_NEGATIVE_REGULATION_OF_WOUND_HEALING | 57 | 2.18171 | 0 | 7.58E-05 |
| GO_CELLULAR_DEFENSE_RESPONSE | 55 | 2.178001 | 0 | 7.51E-05 |
| GO_LYMPHOCYTE_MIGRATION | 41 | 2.175428 | 0 | 8.48E-05 |
| GO_PLASMA_LIPOPROTEIN_PARTICLE_CLEARANCE | 21 | 2.17267 | 0 | 8.41E-05 |
| GO_REGULATION_OF_CYTOKINE_PRODUCTION_INVOLVED_IN_IMMUNE_RESPONSE | 55 | 2.169641 | 0 | 8.34E-05 |
| GO_REGULATION_OF_PHOSPHOLIPID_METABOLIC_PROCESS | 60 | 2.169388 | 0 | 8.28E-05 |
| GO_LEUKOCYTE_PROLIFERATION | 85 | 2.168553 | 0 | 8.21E-05 |
| GO_REGULATION_OF_EPITHELIAL_CELL_APOPTOTIC_PROCESS | 57 | 2.164532 | 0 | 9.15E-05 |
| GO_POSITIVE_REGULATION_OF_B_CELL_PROLIFERATION | 36 | 2.161429 | 0 | 9.08E-05 |
| GO_REGULATION_OF_SMOOTH_MUSCLE_CELL_PROLIFERATION | 98 | 2.159786 | 0 | 9.01E-05 |
| GO_POSITIVE_REGULATION_OF_LYMPHOCYTE_DIFFERENTIATION | 79 | 2.157944 | 0 | 8.94E-05 |
| GO_REGULATED_EXOCYTOSIS | 205 | 2.157032 | 0 | 8.87E-05 |
| GO_POSITIVE_REGULATION_OF_DEFENSE_RESPONSE | 341 | 2.15659 | 0 | 8.80E-05 |
| GO_CELLULAR_RESPONSE_TO_INTERFERON_GAMMA | 106 | 2.156056 | 0 | 8.73E-05 |
| GO_DEFENSE_RESPONSE_TO_BACTERIUM | 184 | 2.153356 | 0 | 9.63E-05 |
| GO_POSITIVE_REGULATION_OF_MAPK_CASCADE | 438 | 2.153254 | 0 | 9.56E-05 |
| GO_IMMUNE_EFFECTOR_PROCESS | 428 | 2.152692 | 0 | 9.49E-05 |
| GO_RESPONSE_TO_OXYGEN_LEVELS | 303 | 2.152268 | 0 | 9.42E-05 |
| GO_POSITIVE_REGULATION_OF_ALPHA_BETA_T_CELL_ACTIVATION | 51 | 2.150722 | 0 | 9.35E-05 |
| GO_ACUTE_PHASE_RESPONSE | 39 | 2.149625 | 0 | 9.28E-05 |
| GO_POSITIVE_REGULATION_OF_REACTIVE_OXYGEN_SPECIES_BIOSYNTHETIC_PROCESS | 46 | 2.149564 | 0 | 9.22E-05 |
| GO_REGULATION_OF_HEAT_GENERATION | 15 | 2.149446 | 0 | 9.15E-05 |
| GO_REGULATION_OF_CYTOSOLIC_CALCIUM_ION_CONCENTRATION | 191 | 2.147809 | 0 | 1.09E-04 |
| GO_SINGLE_ORGANISM_CELL_ADHESION | 444 | 2.147492 | 0 | 1.08E-04 |
| GO_PHAGOCYTOSIS_RECOGNITION | 20 | 2.146131 | 0 | 1.08E-04 |
| GO_PROTEIN_LIPID_COMPLEX_ASSEMBLY | 21 | 2.137753 | 0 | 1.07E-04 |
| GO_REGULATION_OF_PROTEIN_MATURATION | 72 | 2.137061 | 0 | 1.06E-04 |
| GO_REGULATION_OF_OSSIFICATION | 170 | 2.135539 | 0 | 1.14E-04 |
| GO_POSITIVE_REGULATION_OF_CYTOKINE_PRODUCTION | 348 | 2.133012 | 0 | 1.31E-04 |
| GO_NEGATIVE_REGULATION_OF_LEUKOCYTE_APOPTOTIC_PROCESS | 42 | 2.130113 | 0 | 1.38E-04 |
| GO_LEUKOCYTE_CELL_CELL_ADHESION | 248 | 2.128785 | 0 | 1.46E-04 |
| GO_MACROMOLECULAR_COMPLEX_REMODELING | 24 | 2.126363 | 0 | 1.53E-04 |
| GO_REGULATION_OF_ALPHA_BETA_T_CELL_ACTIVATION | 68 | 2.124926 | 0 | 1.61E-04 |
| GO_POSITIVE_REGULATION_OF_LIPID_KINASE_ACTIVITY | 32 | 2.124474 | 0 | 1.60E-04 |
| GO_REGULATION_OF_REACTIVE_OXYGEN_SPECIES_METABOLIC_PROCESS | 147 | 2.12198 | 0 | 1.67E-04 |
| GO_REGULATION_OF_MONOCYTE_CHEMOTAXIS | 20 | 2.121728 | 0 | 1.66E-04 |
| GO_RESPONSE_TO_FATTY_ACID | 79 | 2.119831 | 0 | 1.65E-04 |
| GO_LEUKOCYTE_ACTIVATION | 399 | 2.116517 | 0 | 1.64E-04 |
| GO_POSITIVE_REGULATION_OF_IMMUNE_EFFECTOR_PROCESS | 148 | 2.114125 | 0 | 1.71E-04 |
| GO_REGULATION_OF_CELL_SUBSTRATE_ADHESION | 165 | 2.112674 | 0 | 1.70E-04 |
| GO_NEGATIVE_REGULATION_OF_LEUKOCYTE_MIGRATION | 28 | 2.112345 | 0 | 1.69E-04 |
| GO_NEGATIVE_REGULATION_OF_TUMOR_NECROSIS_FACTOR_SUPERFAMILY_CYTOKINE_PRODUCTION | 37 | 2.110976 | 0 | 1.76E-04 |
| GO_COLLAGEN_FIBRIL_ORGANIZATION | 36 | 2.10879 | 0 | 1.98E-04 |
| GO_REGULATION_OF_BIOMINERAL_TISSUE_DEVELOPMENT | 68 | 2.105966 | 0 | 2.28E-04 |
| GO_REGULATION_OF_VASOCONSTRICTION | 65 | 2.105012 | 0 | 2.35E-04 |
| GO_NEGATIVE_REGULATION_OF_TRANSPORT | 429 | 2.101672 | 0 | 2.34E-04 |
| GO_CHRONIC_INFLAMMATORY_RESPONSE | 15 | 2.100497 | 0 | 2.56E-04 |
| GO_T_CELL_SELECTION | 36 | 2.100107 | 0 | 2.62E-04 |
| GO_POSITIVE_REGULATION_OF_ALPHA_BETA_T_CELL_DIFFERENTIATION | 37 | 2.099244 | 0 | 2.60E-04 |
| GO_IMMUNE_RESPONSE_REGULATING_CELL_SURFACE_RECEPTOR_SIGNALING_PATHWAY | 281 | 2.096313 | 0 | 2.89E-04 |
| GO_STEROL_TRANSPORT | 49 | 2.095287 | 0 | 2.87E-04 |
| GO_REGULATION_OF_LEUKOCYTE_APOPTOTIC_PROCESS | 74 | 2.094322 | 0 | 2.93E-04 |
| GO_NEGATIVE_REGULATION_OF_CHEMOTAXIS | 46 | 2.093903 | 0 | 2.92E-04 |
| GO_NEGATIVE_REGULATION_OF_COAGULATION | 47 | 2.093869 | 0 | 2.97E-04 |
| GO_DIVALENT_INORGANIC_CATION_HOMEOSTASIS | 322 | 2.093587 | 0 | 3.03E-04 |
| GO_NEGATIVE_REGULATION_OF_EPITHELIAL_CELL_APOPTOTIC_PROCESS | 35 | 2.093476 | 0 | 3.09E-04 |
| GO_REGULATION_OF_EPITHELIAL_CELL_MIGRATION | 162 | 2.092412 | 0 | 3.07E-04 |
| GO_POSITIVE_REGULATION_OF_VASCULAR_ENDOTHELIAL_GROWTH_FACTOR_PRODUCTION | 26 | 2.088059 | 0 | 3.20E-04 |
| GO_REGULATION_OF_PEPTIDYL_TYROSINE_PHOSPHORYLATION | 202 | 2.087361 | 0 | 3.25E-04 |
| GO_NEGATIVE_REGULATION_OF_ENDOTHELIAL_CELL_PROLIFERATION | 30 | 2.087247 | 0.002096 | 3.24E-04 |
| GO_MUSCLE_SYSTEM_PROCESS | 275 | 2.087179 | 0 | 3.22E-04 |
| GO_REGULATION_OF_NON_CANONICAL_WNT_SIGNALING_PATHWAY | 19 | 2.086556 | 0 | 3.20E-04 |
| GO_REGULATION_OF_CELL_CELL_ADHESION | 358 | 2.085546 | 0 | 3.25E-04 |
| GO_REGULATION_OF_LEUKOCYTE_PROLIFERATION | 198 | 2.083712 | 0 | 3.23E-04 |
| GO_NEGATIVE_REGULATION_OF_TRANSPORTER_ACTIVITY | 60 | 2.080666 | 0 | 3.22E-04 |
| GO_REGULATION_OF_CALCIUM_ION_TRANSPORT | 197 | 2.080021 | 0 | 3.34E-04 |
| GO_REGULATION_OF_CD4_POSITIVE_ALPHA_BETA_T_CELL_ACTIVATION | 38 | 2.078805 | 0 | 3.39E-04 |
| GO_RESPONSE_TO_CARBOHYDRATE | 162 | 2.07816 | 0 | 3.51E-04 |
| GO_NEGATIVE_REGULATION_OF_SMOOTH_MUSCLE_CELL_MIGRATION | 16 | 2.077978 | 0 | 3.56E-04 |
| GO_BLOOD_COAGULATION_FIBRIN_CLOT_FORMATION | 24 | 2.077436 | 0 | 3.61E-04 |
| GO_NITRIC_OXIDE_MEDIATED_SIGNAL_TRANSDUCTION | 18 | 2.077151 | 0 | 3.59E-04 |
| GO_NEGATIVE_REGULATION_OF_RESPONSE_TO_WOUNDING | 147 | 2.076794 | 0 | 3.64E-04 |
| GO_RESPIRATORY_BURST | 15 | 2.076668 | 0 | 3.75E-04 |
| GO_POSITIVE_REGULATION_OF_FAT_CELL_DIFFERENTIATION | 47 | 2.073854 | 0 | 4.07E-04 |
| GO_PHAGOCYTOSIS | 156 | 2.072262 | 0 | 4.04E-04 |
| GO_POSITIVE_REGULATION_OF_PROTEIN_KINASE_B_SIGNALING | 77 | 2.070788 | 0 | 4.22E-04 |
| GO_POSITIVE_REGULATION_OF_LEUKOCYTE_DIFFERENTIATION | 126 | 2.070472 | 0 | 4.26E-04 |
| GO_REGULATION_OF_LIPID_KINASE_ACTIVITY | 48 | 2.07021 | 0 | 4.31E-04 |
| GO_REGULATION_OF_PHOSPHATIDYLINOSITOL_3_KINASE_ACTIVITY | 40 | 2.067943 | 0 | 4.35E-04 |
| GO_HEMOSTASIS | 291 | 2.067087 | 0 | 4.39E-04 |
| GO_REGULATION_OF_ALPHA_BETA_T_CELL_DIFFERENTIATION | 46 | 2.066041 | 0 | 4.37E-04 |
| GO_POSITIVE_REGULATION_OF_T_CELL_PROLIFERATION | 93 | 2.063201 | 0 | 4.48E-04 |
| GO_REGULATION_OF_BLOOD_PRESSURE | 163 | 2.060859 | 0 | 4.58E-04 |
| GO_CELLULAR_RESPONSE_TO_INTERLEUKIN_1 | 75 | 2.060406 | 0 | 4.62E-04 |
| GO_CALCIUM_MEDIATED_SIGNALING | 80 | 2.059605 | 0 | 4.85E-04 |
| GO_RESPONSE_TO_INTERFERON_GAMMA | 127 | 2.059509 | 0 | 4.83E-04 |
| GO_POSITIVE_REGULATION_OF_CYTOKINE_BIOSYNTHETIC_PROCESS | 57 | 2.05623 | 0 | 5.49E-04 |
| GO_LYMPHOCYTE_CHEMOTAXIS | 30 | 2.055521 | 0 | 5.59E-04 |
| GO_REGULATION_OF_HOMOTYPIC_CELL_CELL_ADHESION | 290 | 2.055417 | 0 | 5.57E-04 |
| GO_RESPONSE_TO_FUNGUS | 46 | 2.054048 | 0 | 5.73E-04 |
| GO_PHAGOCYTOSIS_ENGULFMENT | 24 | 2.05387 | 0 | 5.70E-04 |
| GO_POSITIVE_REGULATION_OF_RECEPTOR_MEDIATED_ENDOCYTOSIS | 44 | 2.05333 | 0 | 5.79E-04 |
| GO_NEGATIVE_REGULATION_OF_VASCULATURE_DEVELOPMENT | 78 | 2.05158 | 0 | 6.00E-04 |
| GO_REGULATION_OF_CELL_SHAPE | 131 | 2.049163 | 0 | 6.22E-04 |
| GO_NEGATIVE_REGULATION_OF_RESPONSE_TO_EXTERNAL_STIMULUS | 256 | 2.048655 | 0 | 6.25E-04 |
| GO_SMOOTH_MUSCLE_CONTRACTION | 45 | 2.047006 | 0 | 6.34E-04 |
| GO_POSITIVE_REGULATION_OF_COAGULATION | 24 | 2.046126 | 0 | 6.42E-04 |
| GO_RESPONSE_TO_INORGANIC_SUBSTANCE | 457 | 2.045301 | 0 | 6.39E-04 |
| GO_RESPONSE_TO_AMINO_ACID | 106 | 2.044206 | 0 | 6.60E-04 |
| GO_WOUND_HEALING | 441 | 2.041538 | 0 | 6.98E-04 |
| GO_NEGATIVE_REGULATION_OF_EPITHELIAL_CELL_MIGRATION | 51 | 2.040187 | 0 | 7.07E-04 |
| GO_RESPONSE_TO_ACID_CHEMICAL | 297 | 2.039705 | 0 | 7.09E-04 |
| GO_RESPONSE_TO_TRANSFORMING_GROWTH_FACTOR_BETA | 139 | 2.03736 | 0 | 7.24E-04 |
| GO_POSITIVE_REGULATION_OF_STEROID_METABOLIC_PROCESS | 22 | 2.036389 | 0 | 7.32E-04 |
| GO_ACTIVATION_OF_PHOSPHOLIPASE_C_ACTIVITY | 27 | 2.035761 | 0 | 7.40E-04 |
| GO_POSITIVE_REGULATION_OF_PEPTIDYL_TYROSINE_PHOSPHORYLATION | 154 | 2.034707 | 0 | 7.59E-04 |
| GO_OVULATION | 17 | 2.034658 | 0 | 7.56E-04 |
| GO_MULTICELLULAR_ORGANISM_METABOLIC_PROCESS | 90 | 2.032576 | 0 | 7.87E-04 |
| GO_REGULATION_OF_MONONUCLEAR_CELL_MIGRATION | 16 | 2.032333 | 0 | 7.94E-04 |
| GO_REGULATION_OF_PROTEIN_IMPORT | 173 | 2.029482 | 0 | 8.36E-04 |
| GO_POSITIVE_REGULATION_OF_WOUND_HEALING | 46 | 2.028809 | 0 | 8.43E-04 |
| GO_POSITIVE_REGULATION_OF_BLOOD_CIRCULATION | 92 | 2.028767 | 0 | 8.40E-04 |
| GO_POSITIVE_REGULATION_OF_CYTOKINE_SECRETION | 91 | 2.028657 | 0 | 8.41E-04 |
| GO_REGULATION_OF_SEQUESTERING_OF_CALCIUM_ION | 97 | 2.028032 | 0 | 8.43E-04 |
| GO_DISRUPTION_OF_CELLS_OF_OTHER_ORGANISM | 23 | 2.026792 | 0 | 8.56E-04 |
| GO_POSITIVE_REGULATION_OF_CD4_POSITIVE_ALPHA_BETA_T_CELL_ACTIVATION | 27 | 2.026733 | 0 | 8.58E-04 |
| GO_REGULATION_OF_ION_HOMEOSTASIS | 189 | 2.026589 | 0 | 8.54E-04 |
| GO_REGULATION_OF_CELLULAR_RESPONSE_TO_GROWTH_FACTOR_STIMULUS | 220 | 2.024386 | 0 | 9.00E-04 |
| GO_NEGATIVE_REGULATION_OF_IMMUNE_SYSTEM_PROCESS | 343 | 2.024333 | 0 | 8.96E-04 |
| GO_POSITIVE_REGULATION_OF_STAT_CASCADE | 72 | 2.02385 | 0 | 8.92E-04 |
| GO_MUSCLE_HYPERTROPHY | 28 | 2.022047 | 0.00188 | 9.20E-04 |
| GO_B_CELL_PROLIFERATION | 35 | 2.020002 | 0 | 9.49E-04 |
| GO_POSITIVE_REGULATION_OF_TRANSCRIPTION_FACTOR_IMPORT_INTO_NUCLEUS | 51 | 2.01738 | 0 | 9.82E-04 |
| GO_MYD88_DEPENDENT_TOLL_LIKE_RECEPTOR_SIGNALING_PATHWAY | 32 | 2.017341 | 0 | 9.78E-04 |
| GO_RESPONSE_TO_CORTICOSTEROID | 170 | 2.012392 | 0 | 0.001074 |
| GO_NEGATIVE_REGULATION_OF_LIPID_STORAGE | 17 | 2.011245 | 0 | 0.001096 |
| GO_POSITIVE_REGULATION_OF_ION_TRANSPORT | 225 | 2.008111 | 0 | 0.001149 |
| GO_DRUG_METABOLIC_PROCESS | 32 | 2.007606 | 0 | 0.001154 |
| GO_REGULATION_OF_CELL_JUNCTION_ASSEMBLY | 67 | 2.007433 | 0 | 0.00115 |
| GO_LYMPHOCYTE_ACTIVATION | 332 | 2.003717 | 0 | 0.001233 |
| GO_POSITIVE_REGULATION_OF_PHOSPHATASE_ACTIVITY | 26 | 2.003584 | 0.001894 | 0.001228 |
| GO_FIBRINOLYSIS | 21 | 2.00344 | 0 | 0.001228 |
| GO_THYMIC_T_CELL_SELECTION | 19 | 2.002725 | 0 | 0.001233 |
| GO_DETOXIFICATION | 69 | 2.002412 | 0 | 0.001233 |
| GO_ENDOTHELIUM_DEVELOPMENT | 89 | 2.002011 | 0 | 0.001229 |
| GO_POSITIVE_REGULATION_OF_MONOCYTE_CHEMOTAXIS | 15 | 2.00144 | 0.002146 | 0.001244 |
| GO_RESPONSE_TO_HEAT | 85 | 1.999236 | 0 | 0.001249 |
| GO_MUCOPOLYSACCHARIDE_METABOLIC_PROCESS | 105 | 1.999081 | 0 | 0.001244 |
| GO_MUSCLE_STRUCTURE_DEVELOPMENT | 410 | 1.999048 | 0 | 0.001239 |
| GO_AXON_EXTENSION | 36 | 1.998875 | 0 | 0.001239 |
| GO_TOLL_LIKE_RECEPTOR_SIGNALING_PATHWAY | 83 | 1.997995 | 0 | 0.001259 |
| GO_POSITIVE_REGULATION_OF_PROTEIN_SECRETION | 199 | 1.997594 | 0 | 0.001279 |
| GO_CYCLIC_NUCLEOTIDE_BIOSYNTHETIC_PROCESS | 33 | 1.997525 | 0 | 0.001274 |
| GO_RESPONSE_TO_ISOQUINOLINE_ALKALOID | 30 | 1.996911 | 0 | 0.001269 |
| GO_REGULATION_OF_LYMPHOCYTE_MIGRATION | 33 | 1.991805 | 0 | 0.001371 |
| GO_OSSIFICATION | 235 | 1.991745 | 0 | 0.001365 |
| GO_REGULATION_OF_CARDIAC_MUSCLE_CONTRACTION_BY_REGULATION_OF_THE_RELEASE_OF_SEQUESTERED_CALCIUM_ION | 17 | 1.991488 | 0.002045 | 0.00137 |
| GO_POSITIVE_REGULATION_OF_OSSIFICATION | 82 | 1.98973 | 0 | 0.001403 |
| GO_NEGATIVE_REGULATION_OF_MUSCLE_CONTRACTION | 21 | 1.987773 | 0 | 0.00146 |
| GO_REGULATION_OF_TYPE_2_IMMUNE_RESPONSE | 25 | 1.987444 | 0 | 0.001473 |
| GO_REGULATION_OF_SYSTEM_PROCESS | 490 | 1.986452 | 0 | 0.001487 |
| GO_CALCIUM_ION_IMPORT_INTO_CYTOSOL | 39 | 1.984393 | 0 | 0.001538 |
| GO_VASODILATION | 26 | 1.98372 | 0 | 0.001547 |
| GO_REGULATION_OF_METAL_ION_TRANSPORT | 309 | 1.983115 | 0 | 0.001555 |
| GO_POSITIVE_REGULATION_OF_LIPID_STORAGE | 16 | 1.981714 | 0 | 0.001582 |
| GO_POSITIVE_REGULATION_OF_OSTEOBLAST_DIFFERENTIATION | 58 | 1.98095 | 0 | 0.001595 |
| GO_CYTOSOLIC_CALCIUM_ION_TRANSPORT | 49 | 1.980861 | 0 | 0.00159 |
| GO_REGULATION_OF_ANATOMICAL_STRUCTURE_SIZE | 450 | 1.97989 | 0 | 0.001598 |
| GO_REGULATION_OF_MITOCHONDRIAL_FISSION | 15 | 1.977382 | 0 | 0.001661 |
| GO_REGULATION_OF_GLYCOPROTEIN_METABOLIC_PROCESS | 39 | 1.976966 | 0 | 0.001655 |
| GO_PATTERN_RECOGNITION_RECEPTOR_SIGNALING_PATHWAY | 105 | 1.973839 | 0 | 0.001741 |
| GO_ENDOCYTOSIS | 457 | 1.973763 | 0 | 0.001739 |
| GO_CELL_ACTIVATION_INVOLVED_IN_IMMUNE_RESPONSE | 134 | 1.973238 | 0 | 0.001747 |
| GO_CELL_SUBSTRATE_ADHESION | 152 | 1.973068 | 0 | 0.001741 |
| GO_POSITIVE_REGULATION_OF_NF_KAPPAB_IMPORT_INTO_NUCLEUS | 27 | 1.972754 | 0 | 0.001753 |
| GO_POSITIVE_REGULATION_OF_MAP_KINASE_ACTIVITY | 198 | 1.972218 | 0 | 0.00176 |
| GO_NEGATIVE_REGULATION_OF_SYNAPTIC_TRANSMISSION | 59 | 1.970574 | 0 | 0.001808 |
| GO_REGULATION_OF_TUMOR_NECROSIS_FACTOR_BIOSYNTHETIC_PROCESS | 16 | 1.969565 | 0 | 0.001823 |
| GO_RELAXATION_OF_MUSCLE | 20 | 1.968507 | 0.002037 | 0.001862 |
| GO_SPROUTING_ANGIOGENESIS | 43 | 1.968045 | 0 | 0.001868 |
| GO_CALCIUM_ION_IMPORT | 60 | 1.968034 | 0 | 0.001862 |
| GO_MUSCLE_ORGAN_DEVELOPMENT | 259 | 1.967523 | 0 | 0.001864 |
| GO_GLYCEROLIPID_CATABOLIC_PROCESS | 33 | 1.967416 | 0.001754 | 0.001858 |
| GO_CELLULAR_RESPONSE_TO_ACID_CHEMICAL | 160 | 1.967003 | 0 | 0.001869 |
| GO_NEGATIVE_REGULATION_OF_SMOOTH_MUSCLE_CONTRACTION | 15 | 1.965264 | 0.002024 | 0.001898 |
| GO_LIPOPOLYSACCHARIDE_MEDIATED_SIGNALING_PATHWAY | 30 | 1.964873 | 0 | 0.001895 |
| GO_RESPONSE_TO_METAL_ION | 318 | 1.964068 | 0 | 0.00192 |
| GO_RESPONSE_TO_FLUID_SHEAR_STRESS | 33 | 1.962915 | 0 | 0.001952 |
| GO_AMINOGLYCAN_CATABOLIC_PROCESS | 65 | 1.962902 | 0 | 0.001946 |
| GO_RESPONSE_TO_PURINE_CONTAINING_COMPOUND | 152 | 1.96137 | 0 | 0.001986 |
| GO_POSITIVE_REGULATION_OF_MYELOID_LEUKOCYTE_MEDIATED_IMMUNITY | 18 | 1.961025 | 0.002123 | 0.001984 |
| GO_PROTEIN_SECRETION | 109 | 1.9606 | 0 | 0.002003 |
| GO_REGULATION_OF_LEUKOCYTE_MEDIATED_IMMUNITY | 152 | 1.960299 | 0 | 0.002004 |
| GO_REGULATION_OF_LIPID_METABOLIC_PROCESS | 266 | 1.959947 | 0 | 0.00201 |
| GO_SECRETION_BY_CELL | 459 | 1.959427 | 0 | 0.002016 |
| GO_RESPONSE_TO_MECHANICAL_STIMULUS | 201 | 1.959251 | 0 | 0.00201 |
| GO_REGULATION_OF_FAT_CELL_DIFFERENTIATION | 96 | 1.957161 | 0 | 0.002058 |
| GO_EXTRACELLULAR_MATRIX_DISASSEMBLY | 71 | 1.957145 | 0 | 0.002051 |
| GO_POSITIVE_REGULATION_OF_CELL_JUNCTION_ASSEMBLY | 24 | 1.956158 | 0 | 0.002086 |
| GO_HEART_DEVELOPMENT | 445 | 1.95589 | 0 | 0.002091 |
| GO_PLATELET_AGGREGATION | 36 | 1.951716 | 0 | 0.002246 |
| GO_POSITIVE_REGULATION_OF_VASOCONSTRICTION | 35 | 1.950341 | 0 | 0.0023 |
| GO_REGULATION_OF_INTERLEUKIN_8_PRODUCTION | 55 | 1.948092 | 0 | 0.002383 |
| GO_RUFFLE_ORGANIZATION | 20 | 1.948071 | 0 | 0.002376 |
| GO_RESPONSE_TO_BMP | 88 | 1.947772 | 0 | 0.002384 |
| GO_REGULATION_OF_OSTEOBLAST_DIFFERENTIATION | 109 | 1.947011 | 0 | 0.002389 |
| GO_REGULATION_OF_CELLULAR_EXTRAVASATION | 22 | 1.943758 | 0 | 0.002495 |
| GO_POSITIVE_REGULATION_OF_CALCIUM_ION_TRANSPORT | 99 | 1.942593 | 0 | 0.002532 |
| GO_POSITIVE_REGULATION_OF_HEMOPOIESIS | 158 | 1.941766 | 0 | 0.002556 |
| GO_CELL_MATRIX_ADHESION | 107 | 1.94144 | 0 | 0.00256 |
| GO_CYCLIC_NUCLEOTIDE_METABOLIC_PROCESS | 55 | 1.939512 | 0 | 0.002652 |
| GO_CARTILAGE_DEVELOPMENT | 140 | 1.939457 | 0 | 0.002648 |
| GO_ANTIGEN_RECEPTOR_MEDIATED_SIGNALING_PATHWAY | 171 | 1.93849 | 0 | 0.002676 |
| GO_POSITIVE_REGULATION_OF_PROTEIN_IMPORT | 99 | 1.93821 | 0 | 0.002675 |
| GO_RESPONSE_TO_TEMPERATURE_STIMULUS | 142 | 1.935253 | 0 | 0.002762 |
| GO_POSITIVE_REGULATION_OF_EPITHELIAL_CELL_MIGRATION | 101 | 1.934254 | 0 | 0.0028 |
| GO_REGULATION_OF_CELL_KILLING | 60 | 1.933785 | 0 | 0.002803 |
| GO_NEGATIVE_REGULATION_OF_LIPID_CATABOLIC_PROCESS | 19 | 1.933722 | 0 | 0.002799 |
| GO_CONNECTIVE_TISSUE_DEVELOPMENT | 186 | 1.932168 | 0 | 0.002826 |
| GO_NEGATIVE_REGULATION_OF_ANION_TRANSPORT | 31 | 1.931669 | 0 | 0.002836 |
| GO_REGULATION_OF_LEUKOCYTE_MEDIATED_CYTOTOXICITY | 50 | 1.931498 | 0 | 0.002832 |
| GO_ORGANIC_HYDROXY_COMPOUND_TRANSPORT | 143 | 1.930606 | 0 | 0.00285 |
| GO_REGULATION_OF_PROTEIN_SECRETION | 367 | 1.930062 | 0 | 0.002864 |
| GO_SULFUR_COMPOUND_CATABOLIC_PROCESS | 38 | 1.929454 | 0 | 0.002875 |
| GO_REGULATION_OF_PHOSPHATIDYLINOSITOL_3_KINASE_SIGNALING | 137 | 1.92923 | 0 | 0.00287 |
| GO_REGULATION_OF_B_CELL_PROLIFERATION | 54 | 1.928119 | 0 | 0.002888 |
| GO_RENAL_SYSTEM_VASCULATURE_DEVELOPMENT | 19 | 1.926452 | 0 | 0.002967 |
| GO_REGULATION_OF_MUSCLE_CONTRACTION | 143 | 1.923328 | 0 | 0.003068 |
| GO_T_CELL_DIFFERENTIATION | 120 | 1.921991 | 0 | 0.003116 |
| GO_REGULATION_OF_ADAPTIVE_IMMUNE_RESPONSE | 122 | 1.921986 | 0 | 0.003111 |
| GO_RESPONSE_TO_TRANSITION_METAL_NANOPARTICLE | 141 | 1.921678 | 0.001669 | 0.003109 |
| GO_RESPONSE_TO_INTERLEUKIN_1 | 100 | 1.921588 | 0 | 0.0031 |
| GO_CGMP_METABOLIC_PROCESS | 23 | 1.921511 | 0.001992 | 0.003091 |
| GO_POSITIVE_REGULATION_OF_PROTEIN_DEPOLYMERIZATION | 17 | 1.920896 | 0.004065 | 0.003108 |
| GO_REGULATION_OF_SYSTEMIC_ARTERIAL_BLOOD_PRESSURE_BY_RENIN_ANGIOTENSIN | 22 | 1.920082 | 0.001931 | 0.003144 |
| GO_MESENCHYME_DEVELOPMENT | 180 | 1.919635 | 0 | 0.003164 |
| GO_RESPONSE_TO_KETONE | 174 | 1.918721 | 0 | 0.003199 |
| GO_FATTY_ACID_DERIVATIVE_BIOSYNTHETIC_PROCESS | 40 | 1.917576 | 0 | 0.003231 |
| GO_POSITIVE_REGULATION_OF_T_HELPER_CELL_DIFFERENTIATION | 18 | 1.91717 | 0.003781 | 0.003257 |
| GO_LEUKOCYTE_DIFFERENTIATION | 284 | 1.917019 | 0 | 0.003252 |
| GO_REGULATION_OF_PROTEIN_KINASE_B_SIGNALING | 115 | 1.915973 | 0 | 0.003293 |
| GO_NEUTRAL_LIPID_METABOLIC_PROCESS | 80 | 1.915366 | 0 | 0.00331 |
| GO_TISSUE_REMODELING | 86 | 1.914995 | 0 | 0.003311 |
| GO_POSITIVE_REGULATION_OF_ENDOTHELIAL_CELL_MIGRATION | 65 | 1.914953 | 0.001631 | 0.003302 |
| GO_POSITIVE_REGULATION_OF_TRANSMEMBRANE_TRANSPORT | 122 | 1.914946 | 0.001684 | 0.003292 |
| GO_POSITIVE_REGULATION_OF_SYNAPTIC_TRANSMISSION_GLUTAMATERGIC | 17 | 1.914892 | 0 | 0.003287 |
| GO_EXOCYTOSIS | 289 | 1.914473 | 0 | 0.003284 |
| GO_REACTIVE_OXYGEN_SPECIES_METABOLIC_PROCESS | 93 | 1.914463 | 0 | 0.003275 |
| GO_POSITIVE_REGULATION_OF_SMOOTH_MUSCLE_CELL_PROLIFERATION | 59 | 1.912304 | 0 | 0.003341 |
| GO_POSITIVE_REGULATION_OF_CHEMOKINE_PRODUCTION | 47 | 1.91176 | 0 | 0.003352 |
| GO_CYTOKINE_MEDIATED_SIGNALING_PATHWAY | 421 | 1.911414 | 0 | 0.003353 |
| GO_REGULATION_OF_AXON_GUIDANCE | 37 | 1.91044 | 0 | 0.003405 |
| GO_NEGATIVE_REGULATION_OF_INTRACELLULAR_TRANSPORT | 130 | 1.910249 | 0 | 0.003406 |
| GO_PROTEIN_KINASE_B_SIGNALING | 33 | 1.909752 | 0 | 0.003414 |
| GO_NEGATIVE_REGULATION_OF_TRANSMEMBRANE_TRANSPORT | 80 | 1.909167 | 0 | 0.003422 |
| GO_MUSCLE_TISSUE_DEVELOPMENT | 261 | 1.9078 | 0 | 0.003472 |
| GO_REGULATION_OF_LIPID_CATABOLIC_PROCESS | 49 | 1.907025 | 0 | 0.003512 |
| GO_REGULATION_OF_VASCULAR_ENDOTHELIAL_GROWTH_FACTOR_RECEPTOR_SIGNALING_PATHWAY | 25 | 1.905675 | 0 | 0.003569 |
| GO_REVERSE_CHOLESTEROL_TRANSPORT | 17 | 1.905577 | 0.002028 | 0.003569 |
| GO_REGULATION_OF_SMOOTH_MUSCLE_CONTRACTION | 59 | 1.90536 | 0 | 0.003563 |
| GO_CYTOLYSIS | 23 | 1.904952 | 0 | 0.003578 |
| GO_POSITIVE_REGULATION_OF_SMOOTH_MUSCLE_CELL_MIGRATION | 29 | 1.904852 | 0 | 0.003571 |
| GO_MULTICELLULAR_ORGANISMAL_MACROMOLECULE_METABOLIC_PROCESS | 76 | 1.904795 | 0 | 0.003562 |
| GO_REGULATION_OF_BLOOD_VESSEL_ENDOTHELIAL_CELL_MIGRATION | 50 | 1.904586 | 0 | 0.003555 |
| GO_MEMBRANE_INVAGINATION | 33 | 1.903891 | 0 | 0.003577 |
| GO_CELLULAR_RESPONSE_TO_VASCULAR_ENDOTHELIAL_GROWTH_FACTOR_STIMULUS | 30 | 1.901061 | 0.001908 | 0.003727 |
| GO_REGULATION_OF_TYROSINE_PHOSPHORYLATION_OF_STAT_PROTEIN | 67 | 1.901001 | 0.001739 | 0.003717 |
| GO_RESPONSE_TO_MONOAMINE | 35 | 1.899783 | 0 | 0.003771 |
| GO_REGULATION_OF_LEUKOCYTE_DIFFERENTIATION | 221 | 1.898374 | 0 | 0.003833 |
| GO_CALCIUM_ION_TRANSPORT | 199 | 1.895386 | 0 | 0.003961 |
| GO_LYMPHOCYTE_DIFFERENTIATION | 204 | 1.895172 | 0 | 0.003964 |
| GO_REGULATION_OF_MACROPHAGE_CHEMOTAXIS | 16 | 1.893491 | 0.002123 | 0.004044 |
| GO_RETINA_VASCULATURE_DEVELOPMENT_IN_CAMERA_TYPE_EYE | 16 | 1.892599 | 0 | 0.004073 |
| GO_REGULATION_OF_TRANSPORTER_ACTIVITY | 189 | 1.89256 | 0 | 0.004063 |
| GO_RECEPTOR_MEDIATED_ENDOCYTOSIS | 201 | 1.891985 | 0 | 0.004102 |
| GO_FIBRIL_ORGANIZATION | 18 | 1.891852 | 0.003817 | 0.004102 |
| GO_MAST_CELL_ACTIVATION | 21 | 1.891781 | 0 | 0.004091 |
| GO_T_CELL_PROLIFERATION | 35 | 1.891408 | 0 | 0.00409 |
| GO_RESPONSE_TO_PROSTAGLANDIN_E | 25 | 1.889152 | 0 | 0.004195 |
| GO_CGMP_BIOSYNTHETIC_PROCESS | 15 | 1.888696 | 0.002033 | 0.004207 |
| GO_NEGATIVE_REGULATION_OF_G_PROTEIN_COUPLED_RECEPTOR_PROTEIN_SIGNALING_PATHWAY | 38 | 1.888645 | 0 | 0.004203 |
| GO_POSITIVE_REGULATION_OF_GLYCOPROTEIN_METABOLIC_PROCESS | 17 | 1.888645 | 0.002049 | 0.004192 |
| GO_POSITIVE_REGULATION_OF_DEPHOSPHORYLATION | 42 | 1.887672 | 0 | 0.004224 |
| GO_POSITIVE_REGULATION_OF_TYROSINE_PHOSPHORYLATION_OF_STAT3_PROTEIN | 37 | 1.887097 | 0 | 0.004246 |
| GO_REGULATION_OF_POSITIVE_CHEMOTAXIS | 22 | 1.885984 | 0.003906 | 0.004287 |
| GO_REGULATION_OF_TRANSCRIPTION_FACTOR_IMPORT_INTO_NUCLEUS | 91 | 1.885726 | 0 | 0.004302 |
| GO_POSITIVE_REGULATION_OF_LYMPHOCYTE_MIGRATION | 25 | 1.885355 | 0.001912 | 0.004304 |
| GO_REGULATION_OF_CHEMOKINE_PRODUCTION | 62 | 1.885233 | 0 | 0.004302 |
| GO_NEGATIVE_REGULATION_OF_IMMUNE_RESPONSE | 117 | 1.884625 | 0.001661 | 0.004314 |
| GO_REGULATION_OF_IMMUNE_EFFECTOR_PROCESS | 400 | 1.884062 | 0 | 0.004319 |
| GO_NEGATIVE_REGULATION_OF_REGULATED_SECRETORY_PATHWAY | 21 | 1.883873 | 0.004024 | 0.004318 |
| GO_POSITIVE_REGULATION_OF_CELL_ADHESION_MEDIATED_BY_INTEGRIN | 17 | 1.883757 | 0.002024 | 0.004307 |
| GO_REGULATION_OF_CELL_ADHESION_MEDIATED_BY_INTEGRIN | 38 | 1.883218 | 0 | 0.004309 |
| GO_POSITIVE_REGULATION_OF_SECRETION | 353 | 1.882966 | 0 | 0.004308 |
| GO_CYTOKINE_PRODUCTION_INVOLVED_IN_IMMUNE_RESPONSE | 17 | 1.88242 | 0 | 0.004329 |
| GO_REGULATION_OF_SPROUTING_ANGIOGENESIS | 27 | 1.88239 | 0 | 0.004321 |
| GO_REGULATION_OF_OSTEOCLAST_DIFFERENTIATION | 58 | 1.881607 | 0 | 0.004358 |
| GO_REGULATION_OF_EXTRINSIC_APOPTOTIC_SIGNALING_PATHWAY_VIA_DEATH_DOMAIN_RECEPTORS | 52 | 1.88155 | 0 | 0.004347 |
| GO_RESPONSE_TO_LIPOPROTEIN_PARTICLE | 20 | 1.879442 | 0 | 0.00444 |
| GO_PHOSPHATIDYLINOSITOL_3_KINASE_SIGNALING | 23 | 1.878859 | 0 | 0.004457 |
| GO_NEGATIVE_REGULATION_OF_CYTOKINE_PRODUCTION_INVOLVED_IN_IMMUNE_RESPONSE | 22 | 1.878124 | 0.001916 | 0.004477 |
| GO_REGULATION_OF_CELL_MORPHOGENESIS_INVOLVED_IN_DIFFERENTIATION | 324 | 1.87673 | 0 | 0.004544 |
| GO_PLATELET_ACTIVATION | 137 | 1.876538 | 0 | 0.004545 |
| GO_NEGATIVE_REGULATION_OF_CELL_ACTIVATION | 150 | 1.876239 | 0 | 0.004547 |
| GO_NEGATIVE_CHEMOTAXIS | 38 | 1.87418 | 0 | 0.004656 |
| GO_RESPONSE_TO_REACTIVE_OXYGEN_SPECIES | 187 | 1.8741 | 0 | 0.004648 |
| GO_NEGATIVE_REGULATION_OF_CELL_ADHESION | 212 | 1.874047 | 0 | 0.004637 |
| GO_PURINERGIC_RECEPTOR_SIGNALING_PATHWAY | 26 | 1.873905 | 0 | 0.004632 |
| GO_REGULATION_OF_RELEASE_OF_SEQUESTERED_CALCIUM_ION_INTO_CYTOSOL_BY_SARCOPLASMIC_RETICULUM | 23 | 1.871592 | 0.001938 | 0.004764 |
| GO_REGULATION_OF_PRODUCTION_OF_MOLECULAR_MEDIATOR_OF_IMMUNE_RESPONSE | 97 | 1.87148 | 0 | 0.004753 |
| GO_CELLULAR_RESPONSE_TO_LIPID | 426 | 1.867824 | 0 | 0.004975 |
| GO_RESPONSE_TO_GROWTH_FACTOR | 456 | 1.866496 | 0 | 0.005051 |
| GO_REGULATION_OF_MAP_KINASE_ACTIVITY | 307 | 1.864768 | 0 | 0.005144 |
| GO_REGULATION_OF_MUSCLE_SYSTEM_PROCESS | 190 | 1.864687 | 0 | 0.005141 |
| GO_REGULATION_OF_B_CELL_DIFFERENTIATION | 22 | 1.863763 | 0.00381 | 0.005207 |
| GO_RECEPTOR_INTERNALIZATION | 47 | 1.863079 | 0.001852 | 0.005222 |
| GO_PURINERGIC_NUCLEOTIDE_RECEPTOR_SIGNALING_PATHWAY | 21 | 1.863012 | 0 | 0.005216 |
| GO_REGULATION_OF_STAT_CASCADE | 139 | 1.862917 | 0 | 0.005203 |
| GO_RESPONSE_TO_ZINC_ION | 51 | 1.861905 | 0.001786 | 0.005245 |
| GO_NEGATIVE_REGULATION_OF_TRANSMEMBRANE_RECEPTOR_PROTEIN_SERINE_THREONINE_KINASE_SIGNALING_PATHWAY | 97 | 1.861383 | 0 | 0.005268 |
| GO_MUSCLE_CONTRACTION | 226 | 1.860694 | 0 | 0.005321 |
| GO_REGULATION_OF_CARTILAGE_DEVELOPMENT | 59 | 1.859901 | 0 | 0.005359 |
| GO_POSITIVE_REGULATION_OF_INTERLEUKIN_6_PRODUCTION | 64 | 1.859641 | 0 | 0.005371 |
| GO_ENDOTHELIAL_CELL_DIFFERENTIATION | 71 | 1.859439 | 0 | 0.005385 |
| GO_REGULATION_OF_HETEROTYPIC_CELL_CELL_ADHESION | 18 | 1.859157 | 0.003976 | 0.005375 |
| GO_POSITIVE_REGULATION_OF_INTERFERON_GAMMA_PRODUCTION | 59 | 1.859155 | 0 | 0.005363 |
| GO_HEART_MORPHOGENESIS | 205 | 1.85817 | 0 | 0.005403 |
| GO_DETECTION_OF_BIOTIC_STIMULUS | 23 | 1.857899 | 0 | 0.005405 |
| GO_APOPTOTIC_CELL_CLEARANCE | 26 | 1.856985 | 0.001898 | 0.005439 |
| GO_LYMPHOCYTE_COSTIMULATION | 71 | 1.856085 | 0 | 0.0055 |
| GO_POSITIVE_REGULATION_OF_FATTY_ACID_METABOLIC_PROCESS | 33 | 1.855618 | 0 | 0.005511 |
| GO_POSITIVE_REGULATION_OF_KINASE_ACTIVITY | 456 | 1.855334 | 0 | 0.00551 |
| GO_REGULATION_OF_MEMBRANE_PROTEIN_ECTODOMAIN_PROTEOLYSIS | 21 | 1.85467 | 0.001984 | 0.005535 |
| GO_POSITIVE_T_CELL_SELECTION | 21 | 1.854466 | 0.002045 | 0.005545 |
| GO_NEGATIVE_REGULATION_OF_BIOMINERAL_TISSUE_DEVELOPMENT | 17 | 1.854346 | 0.011278 | 0.005541 |
| GO_RENAL_SYSTEM_PROCESS | 98 | 1.853856 | 0 | 0.005558 |
| GO_BROWN_FAT_CELL_DIFFERENTIATION | 30 | 1.853827 | 0 | 0.005545 |
| GO_POSITIVE_REGULATION_OF_INTERLEUKIN_8_PRODUCTION | 43 | 1.85349 | 0.00381 | 0.005558 |
| GO_REGULATION_OF_T_HELPER_CELL_DIFFERENTIATION | 26 | 1.8533 | 0 | 0.005563 |
| GO_CYCLIC_NUCLEOTIDE_MEDIATED_SIGNALING | 46 | 1.850337 | 0 | 0.00573 |
| GO_TRIGLYCERIDE_CATABOLIC_PROCESS | 19 | 1.849317 | 0.002041 | 0.00578 |
| GO_NEGATIVE_REGULATION_OF_CATION_CHANNEL_ACTIVITY | 31 | 1.849051 | 0 | 0.005796 |
| GO_POSITIVE_REGULATION_OF_PROTEIN_SERINE_THREONINE_KINASE_ACTIVITY | 272 | 1.848982 | 0 | 0.005785 |
| GO_SUBSTRATE_ADHESION_DEPENDENT_CELL_SPREADING | 38 | 1.848904 | 0.001779 | 0.005773 |
| GO_REGULATION_OF_CALCIUM_ION_IMPORT | 92 | 1.848562 | 0 | 0.005777 |
| GO_POSITIVE_REGULATION_OF_I_KAPPAB_KINASE_NF_KAPPAB_SIGNALING | 168 | 1.847867 | 0 | 0.005798 |
| GO_REGULATION_OF_INTERLEUKIN_6_PRODUCTION | 95 | 1.847662 | 0 | 0.005802 |
| GO_CELLULAR_RESPONSE_TO_CARBOHYDRATE_STIMULUS | 72 | 1.847372 | 0 | 0.005809 |
| GO_FOREBRAIN_CELL_MIGRATION | 61 | 1.847229 | 0 | 0.005818 |
| GO_NEGATIVE_REGULATION_OF_ALPHA_BETA_T_CELL_ACTIVATION | 23 | 1.844288 | 0 | 0.006009 |
| GO_RESPONSE_TO_ALKALOID | 133 | 1.841781 | 0 | 0.006199 |
| GO_REGULATION_OF_CARDIAC_MUSCLE_CONTRACTION_BY_CALCIUM_ION_SIGNALING | 21 | 1.841418 | 0.001845 | 0.006213 |
| GO_REGULATION_OF_ACTIN_FILAMENT_BASED_PROCESS | 300 | 1.84114 | 0 | 0.006224 |
| GO_NEGATIVE_REGULATION_OF_CALCIUM_ION_TRANSMEMBRANE_TRANSPORT | 26 | 1.84095 | 0.003831 | 0.006225 |
| GO_REGULATION_OF_LIPOPROTEIN_LIPASE_ACTIVITY | 15 | 1.840331 | 0 | 0.006266 |
| GO_ATRIOVENTRICULAR_VALVE_MORPHOGENESIS | 16 | 1.839423 | 0.003976 | 0.006308 |
| GO_MONOAMINE_TRANSPORT | 22 | 1.839109 | 0 | 0.006313 |
| GO_REGULATION_OF_SYSTEMIC_ARTERIAL_BLOOD_PRESSURE_BY_HORMONE | 35 | 1.838672 | 0.003752 | 0.006319 |
| GO_B_CELL_ACTIVATION | 127 | 1.838562 | 0 | 0.006322 |
| GO_HIGH_DENSITY_LIPOPROTEIN_PARTICLE_REMODELING | 15 | 1.836943 | 0 | 0.006406 |
| GO_REGULATION_OF_BLOOD_CIRCULATION | 286 | 1.83594 | 0 | 0.006464 |
| GO_REGULATION_OF_INTERLEUKIN_8_SECRETION | 18 | 1.834784 | 0.002075 | 0.00654 |
| GO_POSITIVE_REGULATION_OF_CALCIUM_MEDIATED_SIGNALING | 35 | 1.834621 | 0 | 0.006545 |
| GO_REGULATION_OF_RENAL_SYSTEM_PROCESS | 37 | 1.834225 | 0.00189 | 0.006555 |
| GO_REGULATION_OF_HEMOPOIESIS | 295 | 1.83313 | 0 | 0.006603 |
| GO_G_PROTEIN_COUPLED_PURINERGIC_RECEPTOR_SIGNALING_PATHWAY | 18 | 1.830779 | 0.00207 | 0.00678 |
| GO_REGULATION_OF_CATION_TRANSMEMBRANE_TRANSPORT | 200 | 1.829883 | 0 | 0.006833 |
| GO_DEVELOPMENTAL_CELL_GROWTH | 74 | 1.826933 | 0.001727 | 0.007081 |
| GO_CELL_RECOGNITION | 127 | 1.826665 | 0 | 0.007082 |
| GO_POSITIVE_REGULATION_OF_CELL_DEVELOPMENT | 450 | 1.824078 | 0 | 0.007281 |
| GO_POSITIVE_REGULATION_OF_NEURON_DIFFERENTIATION | 291 | 1.823919 | 0 | 0.007276 |
| GO_NEGATIVE_REGULATION_OF_CALCIUM_ION_TRANSPORT | 46 | 1.823639 | 0 | 0.007296 |
| GO_RESPONSE_TO_ATP | 27 | 1.822318 | 0.003922 | 0.007392 |
| GO_REGULATION_OF_AMINE_TRANSPORT | 68 | 1.820674 | 0.003497 | 0.007503 |
| GO_REGULATION_OF_CATECHOLAMINE_SECRETION | 42 | 1.819809 | 0 | 0.007541 |
| GO_MYELOID_LEUKOCYTE_MEDIATED_IMMUNITY | 40 | 1.81844 | 0.001825 | 0.007667 |
| GO_AMINOGLYCAN_METABOLIC_PROCESS | 162 | 1.817854 | 0 | 0.007696 |
| GO_RECEPTOR_METABOLIC_PROCESS | 77 | 1.817357 | 0.001815 | 0.007717 |
| GO_NEGATIVE_REGULATION_OF_BMP_SIGNALING_PATHWAY | 40 | 1.816153 | 0.001919 | 0.007813 |
| GO_FAT_CELL_DIFFERENTIATION | 102 | 1.815805 | 0 | 0.007831 |
| GO_NEGATIVE_REGULATION_OF_SECRETION | 189 | 1.815617 | 0 | 0.007833 |
| GO_REGULATION_OF_ENDOTHELIAL_CELL_DIFFERENTIATION | 27 | 1.815433 | 0.001908 | 0.007836 |
| GO_POSITIVE_REGULATION_OF_PROTEIN_COMPLEX_DISASSEMBLY | 22 | 1.814243 | 0 | 0.007913 |
| GO_REGULATION_OF_LYMPHOCYTE_DIFFERENTIATION | 129 | 1.814179 | 0 | 0.007899 |
| GO_REGULATION_OF_ENDOTHELIAL_CELL_CHEMOTAXIS | 17 | 1.813685 | 0.005769 | 0.00795 |
| GO_SECONDARY_METABOLIC_PROCESS | 44 | 1.813304 | 0 | 0.007968 |
| GO_REGULATION_OF_T_CELL_MIGRATION | 24 | 1.812953 | 0.003752 | 0.007972 |
| GO_POSITIVE_REGULATION_OF_CALCIUM_ION_IMPORT | 47 | 1.812925 | 0.001946 | 0.007961 |
| GO_FATTY_ACID_DERIVATIVE_METABOLIC_PROCESS | 87 | 1.812857 | 0 | 0.00795 |
| GO_SYNAPSE_ORGANIZATION | 141 | 1.811225 | 0 | 0.008075 |
| GO_REGULATION_OF_TRANSMEMBRANE_TRANSPORT | 402 | 1.810559 | 0 | 0.008141 |
| GO_DEFENSE_RESPONSE_TO_FUNGUS | 34 | 1.810024 | 0.001953 | 0.00817 |
| GO_REGULATION_OF_NITRIC_OXIDE_SYNTHASE_BIOSYNTHETIC_PROCESS | 17 | 1.809524 | 0.003891 | 0.008169 |
| GO_NEGATIVE_REGULATION_OF_BLOOD_VESSEL_ENDOTHELIAL_CELL_MIGRATION | 23 | 1.807429 | 0.001953 | 0.008366 |
| GO_BLOOD_COAGULATION_INTRINSIC_PATHWAY | 17 | 1.805331 | 0 | 0.008521 |
| GO_RESPONSE_TO_ACIDIC_PH | 16 | 1.804937 | 0.001898 | 0.00857 |
| GO_RESPONSE_TO_PAIN | 28 | 1.803978 | 0 | 0.00864 |
| GO_POSITIVE_REGULATION_OF_BLOOD_VESSEL_ENDOTHELIAL_CELL_MIGRATION | 25 | 1.803476 | 0.00369 | 0.008678 |
| GO_POSITIVE_REGULATION_OF_ANTIGEN_PROCESSING_AND_PRESENTATION | 16 | 1.803443 | 0 | 0.008663 |
| GO_AMINOGLYCAN_BIOSYNTHETIC_PROCESS | 105 | 1.803309 | 0 | 0.008656 |
| GO_NEGATIVE_REGULATION_OF_CATION_TRANSMEMBRANE_TRANSPORT | 58 | 1.802348 | 0.003571 | 0.008717 |
| GO_REGULATION_OF_ACTIN_FILAMENT_LENGTH | 146 | 1.80004 | 0 | 0.008957 |
| GO_RESPONSE_TO_ORGANOPHOSPHORUS | 133 | 1.800019 | 0 | 0.008942 |
| GO_NEUTROPHIL_MEDIATED_IMMUNITY | 21 | 1.79985 | 0.005952 | 0.008939 |
| GO_POSITIVE_REGULATION_OF_MUSCLE_CONTRACTION | 43 | 1.799608 | 0 | 0.008956 |
| GO_NEGATIVE_REGULATION_OF_CYCLIC_NUCLEOTIDE_METABOLIC_PROCESS | 41 | 1.79938 | 0.005556 | 0.008963 |
| GO_NEGATIVE_REGULATION_OF_MUSCLE_CELL_APOPTOTIC_PROCESS | 29 | 1.799251 | 0.005445 | 0.008956 |
| GO_POSITIVE_REGULATION_OF_FILOPODIUM_ASSEMBLY | 24 | 1.799046 | 0 | 0.008965 |
| GO_RESPIRATORY_SYSTEM_DEVELOPMENT | 193 | 1.798399 | 0 | 0.009015 |
| GO_AORTA_DEVELOPMENT | 41 | 1.798084 | 0.003617 | 0.009022 |
| GO_NEGATIVE_REGULATION_OF_CELLULAR_PROTEIN_LOCALIZATION | 127 | 1.796129 | 0 | 0.009218 |
| GO_REGULATION_OF_CARDIAC_MUSCLE_CONTRACTION | 63 | 1.796066 | 0.001802 | 0.009205 |
| GO_REGULATION_OF_ADHERENS_JUNCTION_ORGANIZATION | 49 | 1.79572 | 0.003636 | 0.009214 |
| GO_REGULATION_OF_BODY_FLUID_LEVELS | 477 | 1.794776 | 0 | 0.00927 |
| GO_POSITIVE_REGULATION_OF_CELL_MORPHOGENESIS_INVOLVED_IN_DIFFERENTIATION | 157 | 1.792943 | 0 | 0.009408 |
| GO_CELLULAR_RESPONSE_TO_CAMP | 48 | 1.792905 | 0.003617 | 0.009397 |
| GO_ENDODERMAL_CELL_DIFFERENTIATION | 38 | 1.792631 | 0.003795 | 0.009404 |
| GO_NEGATIVE_REGULATION_OF_PROTEIN_LOCALIZATION_TO_CELL_PERIPHERY | 19 | 1.792469 | 0 | 0.009398 |
| GO_HETEROPHILIC_CELL_CELL_ADHESION_VIA_PLASMA_MEMBRANE_CELL_ADHESION_MOLECULES | 42 | 1.791419 | 0.001842 | 0.00952 |
| GO_SUBSTRATE_DEPENDENT_CELL_MIGRATION | 27 | 1.791232 | 0.001905 | 0.009529 |
| GO_DIGESTIVE_SYSTEM_DEVELOPMENT | 142 | 1.790799 | 0 | 0.009569 |
| GO_ENDOTHELIAL_CELL_MIGRATION | 54 | 1.790771 | 0 | 0.009551 |
| GO_POSITIVE_REGULATION_OF_CELL_PROJECTION_ORGANIZATION | 292 | 1.790698 | 0 | 0.009549 |
| GO_REGULATION_OF_INTERLEUKIN_12_PRODUCTION | 51 | 1.790524 | 0.00365 | 0.009548 |
| GO_NEGATIVE_REGULATION_OF_CYTOKINE_SECRETION | 40 | 1.790027 | 0.005329 | 0.009592 |
| GO_RESPONSE_TO_OXIDATIVE_STRESS | 337 | 1.789256 | 0 | 0.009655 |
| GO_STEM_CELL_DIFFERENTIATION | 186 | 1.788313 | 0 | 0.009764 |
| GO_REGULATION_OF_INTERFERON_GAMMA_PRODUCTION | 87 | 1.78823 | 0 | 0.00975 |
| GO_POSITIVE_REGULATION_OF_NF_KAPPAB_TRANSCRIPTION_FACTOR_ACTIVITY | 126 | 1.787588 | 0 | 0.009803 |
| GO_AGING | 257 | 1.787255 | 0 | 0.009816 |
| GO_CELL_MORPHOGENESIS_INVOLVED_IN_DIFFERENTIATION | 492 | 1.78704 | 0 | 0.009816 |
| GO_CELLULAR_RESPONSE_TO_ORGANIC_CYCLIC_COMPOUND | 438 | 1.786695 | 0 | 0.009836 |
| GO_REGULATION_OF_HYDROGEN_PEROXIDE_METABOLIC_PROCESS | 15 | 1.786351 | 0.01217 | 0.00986 |
| GO_REGULATION_OF_LYMPHOCYTE_MEDIATED_IMMUNITY | 110 | 1.785888 | 0 | 0.009898 |
| GO_REGULATION_OF_T_CELL_PROLIFERATION | 141 | 1.785235 | 0 | 0.009951 |
| GO_POSITIVE_REGULATION_OF_SUBSTRATE_ADHESION_DEPENDENT_CELL_SPREADING | 27 | 1.785192 | 0.004065 | 0.009935 |
| GO_EPOXYGENASE_P450_PATHWAY | 18 | 1.784676 | 0.001969 | 0.009975 |
| GO_SKELETAL_MUSCLE_ORGAN_DEVELOPMENT | 128 | 1.784631 | 0 | 0.009968 |
| GO_REGULATION_OF_AXONOGENESIS | 161 | 1.783426 | 0 | 0.010074 |
| GO_NEGATIVE_REGULATION_OF_CYTOKINE_PRODUCTION | 192 | 1.782532 | 0 | 0.010167 |
| GO_REGULATION_OF_CALCIUM_ION_TRANSMEMBRANE_TRANSPORTER_ACTIVITY | 66 | 1.78251 | 0 | 0.010153 |
| GO_REGULATION_OF_TOLERANCE_INDUCTION | 17 | 1.781631 | 0.004016 | 0.010232 |
| GO_MUSCLE_CELL_DIFFERENTIATION | 226 | 1.781221 | 0 | 0.010255 |
| GO_NEGATIVE_REGULATION_OF_GLUCOSE_TRANSPORT | 15 | 1.781073 | 0.00396 | 0.010246 |
| GO_NEGATIVE_REGULATION_OF_BLOOD_CIRCULATION | 34 | 1.78098 | 0.001923 | 0.010236 |
| GO_REGULATION_OF_PLATELET_ACTIVATION | 30 | 1.779935 | 0 | 0.010307 |
| GO_RESPONSE_TO_XENOBIOTIC_STIMULUS | 89 | 1.779917 | 0 | 0.01029 |
| GO_CALCIUM_ION_TRANSMEMBRANE_TRANSPORT | 143 | 1.779655 | 0 | 0.010304 |
| GO_PARTURITION | 19 | 1.778994 | 0.005882 | 0.010379 |
| GO_PHASIC_SMOOTH_MUSCLE_CONTRACTION | 16 | 1.778542 | 0.006024 | 0.010406 |
| GO_REGULATION_OF_PROTEIN_TARGETING | 282 | 1.777406 | 0 | 0.010545 |
| GO_REGULATION_OF_NF_KAPPAB_IMPORT_INTO_NUCLEUS | 45 | 1.777166 | 0.003697 | 0.01056 |
| GO_NEGATIVE_REGULATION_OF_CALCIUM_MEDIATED_SIGNALING | 15 | 1.776627 | 0.004016 | 0.010609 |
| GO_REGULATION_OF_G_PROTEIN_COUPLED_RECEPTOR_PROTEIN_SIGNALING_PATHWAY | 118 | 1.775591 | 0 | 0.010719 |
| GO_REGULATION_OF_I_KAPPAB_KINASE_NF_KAPPAB_SIGNALING | 218 | 1.775378 | 0 | 0.010716 |
| GO_RESPIRATORY_GASEOUS_EXCHANGE | 46 | 1.774773 | 0 | 0.010778 |
| GO_NEGATIVE_REGULATION_OF_CYTOPLASMIC_TRANSPORT | 108 | 1.774106 | 0 | 0.010836 |
| GO_RESPONSE_TO_HYDROPEROXIDE | 15 | 1.773365 | 0 | 0.010924 |
| GO_MATURE_B_CELL_DIFFERENTIATION | 16 | 1.773202 | 0.010331 | 0.010916 |
| GO_REGULATION_OF_DIGESTIVE_SYSTEM_PROCESS | 34 | 1.772863 | 0.001862 | 0.010933 |
| GO_ERK1_AND_ERK2_CASCADE | 22 | 1.772305 | 0 | 0.01099 |
| GO_REGULATION_OF_INTERLEUKIN_1_BETA_PRODUCTION | 44 | 1.772076 | 0.001761 | 0.010989 |
| GO_CELLULAR_RESPONSE_TO_FLUID_SHEAR_STRESS | 19 | 1.771605 | 0.003868 | 0.011016 |
| GO_NEUTRAL_LIPID_CATABOLIC_PROCESS | 24 | 1.771014 | 0.003759 | 0.011077 |
| GO_POSITIVE_REGULATION_OF_STRIATED_MUSCLE_CONTRACTION | 15 | 1.769926 | 0.006 | 0.011158 |
| GO_ASTROCYTE_DEVELOPMENT | 19 | 1.769167 | 0 | 0.011226 |
| GO_PLATELET_DERIVED_GROWTH_FACTOR_RECEPTOR_SIGNALING_PATHWAY | 34 | 1.767663 | 0.003891 | 0.011384 |
| GO_POSITIVE_REGULATION_OF_LIPID_BIOSYNTHETIC_PROCESS | 61 | 1.76762 | 0.001805 | 0.011366 |
| GO_NEGATIVE_REGULATION_OF_REACTIVE_OXYGEN_SPECIES_BIOSYNTHETIC_PROCESS | 15 | 1.767551 | 0.00404 | 0.01136 |
| GO_RESPONSE_TO_ALCOHOL | 353 | 1.766946 | 0 | 0.011437 |
| GO_ESTROUS_CYCLE | 19 | 1.766826 | 0.003922 | 0.011443 |
| GO_UNSATURATED_FATTY_ACID_BIOSYNTHETIC_PROCESS | 51 | 1.766332 | 0.005445 | 0.011481 |
| GO_POSITIVE_REGULATION_OF_NEURON_PROJECTION_DEVELOPMENT | 226 | 1.766165 | 0 | 0.011485 |
| GO_NEGATIVE_REGULATION_OF_INFLAMMATORY_RESPONSE | 92 | 1.765786 | 0 | 0.011518 |
| GO_CELL_GROWTH | 130 | 1.76574 | 0 | 0.011507 |
| GO_REGULATION_OF_PROTEIN_KINASE_A_SIGNALING | 17 | 1.764696 | 0.004073 | 0.011612 |
| GO_REGULATION_OF_RYANODINE_SENSITIVE_CALCIUM_RELEASE_CHANNEL_ACTIVITY | 23 | 1.764567 | 0.003968 | 0.011607 |
| GO_POSITIVE_REGULATION_OF_MAST_CELL_ACTIVATION | 16 | 1.762623 | 0.00409 | 0.011817 |
| GO_REGULATION_OF_CHONDROCYTE_DIFFERENTIATION | 43 | 1.761975 | 0.001859 | 0.011862 |
| GO_POSITIVE_REGULATION_OF_RECEPTOR_INTERNALIZATION | 22 | 1.76178 | 0 | 0.011868 |
| GO_CELLULAR_RESPONSE_TO_NITROGEN_COMPOUND | 478 | 1.761619 | 0 | 0.011878 |
| GO_POSITIVE_REGULATION_OF_CYTOKINE_PRODUCTION_INVOLVED_IN_IMMUNE_RESPONSE | 28 | 1.761102 | 0.007477 | 0.011921 |
| GO_CELLULAR_RESPONSE_TO_FATTY_ACID | 48 | 1.760363 | 0.003745 | 0.012002 |
| GO_NEGATIVE_REGULATION_OF_NUCLEOTIDE_METABOLIC_PROCESS | 62 | 1.759977 | 0 | 0.012041 |
| GO_REGULATION_OF_CARDIAC_CONDUCTION | 63 | 1.759958 | 0 | 0.012021 |
| GO_REGULATION_OF_CELL_MIGRATION_INVOLVED_IN_SPROUTING_ANGIOGENESIS | 19 | 1.759514 | 0.006073 | 0.012061 |
| GO_REGULATION_OF_MAST_CELL_ACTIVATION | 39 | 1.75912 | 0 | 0.012098 |
| GO_DEVELOPMENTAL_GROWTH_INVOLVED_IN_MORPHOGENESIS | 101 | 1.758948 | 0 | 0.012098 |
| GO_POSITIVE_REGULATION_OF_AXONOGENESIS | 67 | 1.757937 | 0.003454 | 0.012262 |
| GO_MEMORY | 91 | 1.757904 | 0.001678 | 0.012244 |
| GO_CELLULAR_RESPONSE_TO_PROSTAGLANDIN_STIMULUS | 23 | 1.757665 | 0.007505 | 0.012253 |
| GO_VASCULAR_ENDOTHELIAL_GROWTH_FACTOR_RECEPTOR_SIGNALING_PATHWAY | 72 | 1.757376 | 0 | 0.012279 |
| GO_RESPONSE_TO_ACTIVITY | 67 | 1.756784 | 0 | 0.012344 |
| GO_REGULATION_OF_CYTOKINE_BIOSYNTHETIC_PROCESS | 91 | 1.756668 | 0 | 0.012346 |
| GO_POSITIVE_REGULATION_OF_LIPID_CATABOLIC_PROCESS | 25 | 1.756641 | 0 | 0.01233 |
| GO_VASCULOGENESIS | 58 | 1.755851 | 0.001761 | 0.012401 |
| GO_REGULATION_OF_HOMEOSTATIC_PROCESS | 420 | 1.754745 | 0 | 0.01253 |
| GO_DEFENSE_RESPONSE_TO_GRAM_POSITIVE_BACTERIUM | 63 | 1.754692 | 0.001855 | 0.012509 |
| GO_REGULATION_OF_NEURON_PROJECTION_DEVELOPMENT | 394 | 1.754443 | 0 | 0.012516 |
| GO_VASOCONSTRICTION | 28 | 1.754051 | 0.011788 | 0.012544 |
| GO_REGULATION_OF_PEPTIDYL_SERINE_PHOSPHORYLATION | 111 | 1.753944 | 0 | 0.012535 |
| GO_REGULATION_OF_RECEPTOR_MEDIATED_ENDOCYTOSIS | 75 | 1.753697 | 0 | 0.012548 |
| GO_REGULATION_OF_RENAL_SODIUM_EXCRETION | 23 | 1.753235 | 0.007576 | 0.012578 |
| GO_NEGATIVE_REGULATION_OF_RESPONSE_TO_CYTOKINE_STIMULUS | 41 | 1.751337 | 0.001901 | 0.012826 |
| GO_REGULATION_OF_OXIDOREDUCTASE_ACTIVITY | 86 | 1.750601 | 0.001805 | 0.012894 |
| GO_ACTIVATION_OF_MAPK_ACTIVITY | 131 | 1.750234 | 0 | 0.012939 |
| GO_PROSTANOID_METABOLIC_PROCESS | 25 | 1.750168 | 0.005556 | 0.012927 |
| GO_REGULATION_OF_T_HELPER_1_TYPE_IMMUNE_RESPONSE | 22 | 1.748516 | 0.00998 | 0.013131 |
| GO_LUNG_ALVEOLUS_DEVELOPMENT | 41 | 1.748249 | 0.003817 | 0.013141 |
| GO_POSITIVE_REGULATION_OF_LEUKOCYTE_MEDIATED_IMMUNITY | 82 | 1.747842 | 0.001757 | 0.013164 |
| GO_RESPONSE_TO_STEROID_HORMONE | 477 | 1.747236 | 0 | 0.013225 |
| GO_NEGATIVE_REGULATION_OF_EXOCYTOSIS | 27 | 1.746744 | 0.009671 | 0.013263 |
| GO_REGULATION_OF_ANTIGEN_RECEPTOR_MEDIATED_SIGNALING_PATHWAY | 38 | 1.745926 | 0.005535 | 0.013349 |
| GO_POSITIVE_REGULATION_OF_INTERLEUKIN_2_PRODUCTION | 30 | 1.745463 | 0.001923 | 0.013381 |
| GO_NEGATIVE_REGULATION_OF_EPITHELIAL_CELL_PROLIFERATION | 111 | 1.744834 | 0 | 0.013429 |
| GO_MULTI_MULTICELLULAR_ORGANISM_PROCESS | 207 | 1.743812 | 0 | 0.013565 |
| GO_REGULATION_OF_LIPID_TRANSPORT | 93 | 1.742578 | 0.001704 | 0.013747 |
| GO_CENTRAL_NERVOUS_SYSTEM_PROJECTION_NEURON_AXONOGENESIS | 22 | 1.742477 | 0.005952 | 0.013735 |
| GO_POSITIVE_REGULATION_OF_NUCLEOCYTOPLASMIC_TRANSPORT | 116 | 1.742108 | 0 | 0.013778 |
| GO_ORGAN_REGENERATION | 80 | 1.740925 | 0.001764 | 0.013918 |
| GO_MYELOID_CELL_ACTIVATION_INVOLVED_IN_IMMUNE_RESPONSE | 40 | 1.740813 | 0.003759 | 0.013914 |
| GO_REGULATION_OF_DOPAMINE_SECRETION | 22 | 1.74075 | 0.004 | 0.013894 |
| GO_ACTIN_POLYMERIZATION_OR_DEPOLYMERIZATION | 35 | 1.740438 | 0.001873 | 0.013904 |
| GO_POSITIVE_REGULATION_OF_AXON_EXTENSION | 36 | 1.740349 | 0.003824 | 0.013894 |
| GO_HOMOTYPIC_CELL_CELL_ADHESION | 48 | 1.740269 | 0 | 0.013886 |
| GO_POSITIVE_REGULATION_OF_ANION_TRANSPORT | 57 | 1.739758 | 0.001767 | 0.013935 |
| GO_CELL_AGGREGATION | 19 | 1.739148 | 0.011696 | 0.013973 |
| GO_REGULATION_OF_MEMBRANE_LIPID_DISTRIBUTION | 35 | 1.73906 | 0 | 0.013972 |
| GO_HETEROTYPIC_CELL_CELL_ADHESION | 26 | 1.73648 | 0.011628 | 0.014288 |
| GO_REGULATION_OF_ANTIGEN_PROCESSING_AND_PRESENTATION | 23 | 1.735788 | 0.001901 | 0.014381 |
| GO_NEURON_PROJECTION_MORPHOGENESIS | 377 | 1.735356 | 0 | 0.014407 |
| GO_NEGATIVE_REGULATION_OF_VIRAL_ENTRY_INTO_HOST_CELL | 19 | 1.735131 | 0.01217 | 0.014398 |
| GO_REGULATION_OF_CYCLIC_NUCLEOTIDE_METABOLIC_PROCESS | 147 | 1.732254 | 0 | 0.014793 |
| GO_DIVALENT_INORGANIC_CATION_TRANSPORT | 243 | 1.730467 | 0 | 0.015005 |
| GO_REGULATION_OF_RECEPTOR_BINDING | 17 | 1.730052 | 0.01417 | 0.015052 |
| GO_MYELOID_LEUKOCYTE_DIFFERENTIATION | 93 | 1.729924 | 0 | 0.01505 |
| GO_EXTRACELLULAR_MATRIX_ASSEMBLY | 16 | 1.729613 | 0.008333 | 0.015056 |
| GO_REGULATION_OF_OSTEOBLAST_PROLIFERATION | 22 | 1.729607 | 0.011928 | 0.015033 |
| GO_REGULATION_OF_ALPHA_BETA_T_CELL_PROLIFERATION | 23 | 1.7288 | 0.007797 | 0.01512 |
| GO_NEGATIVE_REGULATION_OF_INTRACELLULAR_PROTEIN_TRANSPORT | 89 | 1.728196 | 0.001776 | 0.015201 |
| GO_TOLL_LIKE_RECEPTOR_4_SIGNALING_PATHWAY | 17 | 1.727946 | 0.003788 | 0.015224 |
| GO_REGULATION_OF_EXTRINSIC_APOPTOTIC_SIGNALING_PATHWAY | 145 | 1.726167 | 0 | 0.015493 |
| GO_REFLEX | 19 | 1.725332 | 0.008316 | 0.015598 |
| GO_CHONDROITIN_SULFATE_BIOSYNTHETIC_PROCESS | 25 | 1.725234 | 0.012915 | 0.015582 |
| GO_REGULATION_OF_BONE_DEVELOPMENT | 17 | 1.723725 | 0.016293 | 0.015808 |
| GO_REGULATION_OF_TOLL_LIKE_RECEPTOR_SIGNALING_PATHWAY | 45 | 1.723243 | 0.005376 | 0.015857 |
| GO_REGULATION_OF_NUCLEOCYTOPLASMIC_TRANSPORT | 211 | 1.722455 | 0 | 0.015932 |
| GO_LYMPHOCYTE_ACTIVATION_INVOLVED_IN_IMMUNE_RESPONSE | 94 | 1.721662 | 0.001698 | 0.016024 |
| GO_PALATE_DEVELOPMENT | 83 | 1.721267 | 0 | 0.016082 |
| GO_RESPONSE_TO_HYDROGEN_PEROXIDE | 106 | 1.720433 | 0 | 0.016192 |
| GO_GLIAL_CELL_MIGRATION | 34 | 1.720432 | 0.003781 | 0.016168 |
| GO_RESPONSE_TO_PEPTIDE | 389 | 1.720427 | 0 | 0.016143 |
| GO_LEUKOTRIENE_BIOSYNTHETIC_PROCESS | 19 | 1.720355 | 0.004098 | 0.016128 |
| GO_REGULATION_OF_CALCIUM_ION_TRANSMEMBRANE_TRANSPORT | 109 | 1.719397 | 0 | 0.016233 |
| GO_GAMMA_AMINOBUTYRIC_ACID_SIGNALING_PATHWAY | 21 | 1.717785 | 0.00616 | 0.016481 |
| GO_ARTERY_DEVELOPMENT | 75 | 1.717072 | 0.001667 | 0.016575 |
| GO_REGULATION_OF_EPITHELIAL_TO_MESENCHYMAL_TRANSITION | 65 | 1.716833 | 0.00188 | 0.016573 |
| GO_REGULATION_OF_VESICLE_MEDIATED_TRANSPORT | 436 | 1.715438 | 0 | 0.016808 |
| GO_NEURON_PROJECTION_EXTENSION | 51 | 1.71543 | 0.003571 | 0.016787 |
| GO_BLOOD_VESSEL_ENDOTHELIAL_CELL_MIGRATION | 23 | 1.715359 | 0.001808 | 0.016766 |
| GO_REGULATION_OF_MYELOID_CELL_DIFFERENTIATION | 167 | 1.713649 | 0.001597 | 0.017053 |
| GO_POSITIVE_REGULATION_OF_CYTOSKELETON_ORGANIZATION | 167 | 1.713599 | 0 | 0.017043 |
| GO_PROSTANOID_BIOSYNTHETIC_PROCESS | 18 | 1.713349 | 0.016293 | 0.017061 |
| GO_REGULATION_OF_SYSTEMIC_ARTERIAL_BLOOD_PRESSURE_MEDIATED_BY_A_CHEMICAL_SIGNAL | 45 | 1.712062 | 0.005693 | 0.017265 |
| GO_CELLULAR_RESPONSE_TO_MECHANICAL_STIMULUS | 78 | 1.711348 | 0.001715 | 0.017342 |
| GO_PHOSPHOLIPID_TRANSPORT | 55 | 1.711011 | 0.005474 | 0.017358 |
| GO_REGULATION_OF_CALCIUM_ION_TRANSPORT_INTO_CYTOSOL | 85 | 1.710839 | 0 | 0.017351 |
| GO_RESPONSE_TO_DRUG | 412 | 1.710538 | 0 | 0.017393 |
| GO_REGULATION_OF_T_CELL_DIFFERENTIATION | 104 | 1.710416 | 0.001647 | 0.017376 |
| GO_REGULATION_OF_MAST_CELL_ACTIVATION_INVOLVED_IN_IMMUNE_RESPONSE | 31 | 1.710257 | 0.00722 | 0.017369 |
| GO_REGULATION_OF_TYROSINE_PHOSPHORYLATION_OF_STAT3_PROTEIN | 44 | 1.708672 | 0.00369 | 0.017616 |
| GO_NEGATIVE_REGULATION_OF_HOMEOSTATIC_PROCESS | 117 | 1.707163 | 0 | 0.017848 |
| GO_REGULATION_OF_RELEASE_OF_SEQUESTERED_CALCIUM_ION_INTO_CYTOSOL | 68 | 1.706765 | 0.003534 | 0.017892 |
| GO_NEGATIVE_REGULATION_OF_NUCLEOCYTOPLASMIC_TRANSPORT | 67 | 1.706721 | 0 | 0.017876 |
| GO_REGULATION_OF_MACROPHAGE_ACTIVATION | 23 | 1.706548 | 0.013592 | 0.017893 |
| GO_NEGATIVE_REGULATION_OF_PHOSPHORYLATION | 399 | 1.706446 | 0 | 0.017889 |
| GO_INTERFERON_GAMMA_MEDIATED_SIGNALING_PATHWAY | 63 | 1.705854 | 0.001815 | 0.017956 |
| GO_ACYLGLYCEROL_HOMEOSTASIS | 29 | 1.704757 | 0.005871 | 0.018121 |
| GO_NEGATIVE_REGULATION_OF_INTRINSIC_APOPTOTIC_SIGNALING_PATHWAY_IN_RESPONSE_TO_DNA_DAMAGE | 23 | 1.70417 | 0.006073 | 0.018224 |
| GO_NEGATIVE_REGULATION_OF_CELL_PROJECTION_ORGANIZATION | 137 | 1.703296 | 0 | 0.018311 |
| GO_NEGATIVE_REGULATION_OF_EXTRINSIC_APOPTOTIC_SIGNALING_PATHWAY_VIA_DEATH_DOMAIN_RECEPTORS | 32 | 1.703128 | 0.005682 | 0.018316 |
| GO_CELLULAR_RESPONSE_TO_OXYGEN_LEVELS | 139 | 1.702873 | 0 | 0.018332 |
| GO_POSITIVE_REGULATION_OF_ADAPTIVE_IMMUNE_RESPONSE | 72 | 1.701586 | 0.003472 | 0.018522 |
| GO_POSITIVE_REGULATION_OF_CELL_MATRIX_ADHESION | 40 | 1.701389 | 0.00759 | 0.018533 |
| GO_UNSATURATED_FATTY_ACID_METABOLIC_PROCESS | 98 | 1.701248 | 0.001792 | 0.01853 |
| GO_NEGATIVE_REGULATION_OF_INTERLEUKIN_12_PRODUCTION | 15 | 1.700868 | 0.008 | 0.018589 |
| GO_REGULATION_OF_MYELOID_LEUKOCYTE_DIFFERENTIATION | 100 | 1.699275 | 0.003472 | 0.018871 |
| GO_NEGATIVE_REGULATION_OF_ANOIKIS | 17 | 1.699066 | 0.008457 | 0.018877 |
| GO_BILE_ACID_METABOLIC_PROCESS | 35 | 1.699002 | 0.007605 | 0.018866 |
| GO_POSITIVE_REGULATION_OF_NERVOUS_SYSTEM_DEVELOPMENT | 411 | 1.698693 | 0 | 0.018892 |
| GO_REGULATION_OF_STRIATED_MUSCLE_CONTRACTION | 76 | 1.697993 | 0.001761 | 0.018972 |
| GO_REGULATION_OF_LEUKOCYTE_DEGRANULATION | 41 | 1.697714 | 0.003584 | 0.019005 |
| GO_MYELOID_CELL_DIFFERENTIATION | 181 | 1.696203 | 0 | 0.019229 |
| GO_NEGATIVE_REGULATION_OF_TRANSFORMING_GROWTH_FACTOR_BETA_RECEPTOR_SIGNALING_PATHWAY | 63 | 1.695366 | 0.005172 | 0.019343 |
| GO_POSITIVE_REGULATION_OF_TYROSINE_PHOSPHORYLATION_OF_STAT5_PROTEIN | 16 | 1.694191 | 0.013889 | 0.019521 |
| GO_NEGATIVE_REGULATION_OF_AMINE_TRANSPORT | 24 | 1.693463 | 0.005618 | 0.019639 |
| GO_RESPONSE_TO_TOXIC_SUBSTANCE | 228 | 1.693328 | 0 | 0.019632 |
| GO_ARACHIDONIC_ACID_METABOLIC_PROCESS | 48 | 1.692873 | 0.001883 | 0.019688 |
| GO_STRIATED_MUSCLE_CELL_DIFFERENTIATION | 163 | 1.692635 | 0 | 0.019687 |
| GO_REGULATION_OF_CELL_MATRIX_ADHESION | 86 | 1.692136 | 0.005464 | 0.019746 |
| GO_MODIFICATION_OF_MORPHOLOGY_OR_PHYSIOLOGY_OF_OTHER_ORGANISM | 94 | 1.691907 | 0.001706 | 0.01977 |
| GO_PROTEOGLYCAN_BIOSYNTHETIC_PROCESS | 57 | 1.69187 | 0.005576 | 0.019747 |
| GO_GRANULOCYTE_ACTIVATION | 18 | 1.69081 | 0.007707 | 0.019888 |
| GO_NEGATIVE_REGULATION_OF_INTERLEUKIN_6_PRODUCTION | 30 | 1.690242 | 0.007692 | 0.019948 |
| GO_REGULATION_OF_CATION_CHANNEL_ACTIVITY | 84 | 1.688953 | 0.001721 | 0.02014 |
| GO_REGULATION_OF_PROTEIN_LOCALIZATION_TO_NUCLEUS | 207 | 1.688888 | 0 | 0.020122 |
| GO_CARDIAC_CHAMBER_DEVELOPMENT | 141 | 1.68887 | 0 | 0.020101 |
| GO_TRANSMEMBRANE_RECEPTOR_PROTEIN_SERINE_THREONINE_KINASE_SIGNALING_PATHWAY | 182 | 1.68886 | 0 | 0.020078 |
| GO_REGULATION_OF_TRANSMEMBRANE_RECEPTOR_PROTEIN_SERINE_THREONINE_KINASE_SIGNALING_PATHWAY | 194 | 1.688398 | 0.001548 | 0.020129 |
| GO_CHONDROITIN_SULFATE_PROTEOGLYCAN_BIOSYNTHETIC_PROCESS | 29 | 1.688144 | 0.010101 | 0.020147 |
| GO_KERATAN_SULFATE_METABOLIC_PROCESS | 32 | 1.687611 | 0.007634 | 0.020202 |
| GO_NEGATIVE_REGULATION_OF_EPITHELIAL_TO_MESENCHYMAL_TRANSITION | 22 | 1.687394 | 0.007619 | 0.020223 |
| GO_NEGATIVE_REGULATION_OF_ALPHA_BETA_T_CELL_DIFFERENTIATION | 15 | 1.687263 | 0.015686 | 0.020216 |
| GO_NEGATIVE_REGULATION_OF_ESTABLISHMENT_OF_PROTEIN_LOCALIZATION | 197 | 1.684612 | 0 | 0.020734 |
| GO_POSITIVE_REGULATION_OF_T_HELPER_1_TYPE_IMMUNE_RESPONSE | 15 | 1.684384 | 0.014315 | 0.020755 |
| GO_POSITIVE_REGULATION_OF_CELL_KILLING | 37 | 1.684291 | 0.00759 | 0.020742 |
| GO_REGULATION_OF_INTERLEUKIN_1_PRODUCTION | 53 | 1.683375 | 0.00177 | 0.020887 |
| GO_REGULATION_OF_SKELETAL_MUSCLE_CELL_DIFFERENTIATION | 15 | 1.683087 | 0.003854 | 0.020917 |
| GO_POSITIVE_REGULATION_OF_MEMBRANE_PROTEIN_ECTODOMAIN_PROTEOLYSIS | 15 | 1.68081 | 0.016495 | 0.021325 |
| GO_NEGATIVE_REGULATION_OF_IMMUNE_EFFECTOR_PROCESS | 100 | 1.680104 | 0.00177 | 0.021441 |
| GO_UROGENITAL_SYSTEM_DEVELOPMENT | 295 | 1.680005 | 0 | 0.021443 |
| GO_REGULATION_OF_EXCRETION | 28 | 1.679003 | 0.01145 | 0.021633 |
| GO_PEPTIDYL_TYROSINE_MODIFICATION | 183 | 1.678763 | 0.001548 | 0.021661 |
| GO_POSITIVE_REGULATION_OF_ALPHA_BETA_T_CELL_PROLIFERATION | 19 | 1.678225 | 0.013917 | 0.021717 |
| GO_ALPHA_BETA_T_CELL_DIFFERENTIATION | 44 | 1.677756 | 0.005515 | 0.021785 |
| GO_LIPID_TRANSLOCATION | 21 | 1.674762 | 0.011719 | 0.022333 |
| GO_CENTRAL_NERVOUS_SYSTEM_NEURON_AXONOGENESIS | 27 | 1.67474 | 0.009434 | 0.022309 |
| GO_RESPONSE_TO_MUSCLE_STRETCH | 19 | 1.673452 | 0.011788 | 0.022549 |
| GO_POSITIVE_CHEMOTAXIS | 30 | 1.671542 | 0.009901 | 0.022931 |
| GO_REGULATION_OF_FILOPODIUM_ASSEMBLY | 35 | 1.669591 | 0 | 0.02336 |
| GO_CELLULAR_RESPONSE_TO_DRUG | 64 | 1.669459 | 0.001742 | 0.023365 |
| GO_CELLULAR_RESPONSE_TO_EXTERNAL_STIMULUS | 251 | 1.669156 | 0 | 0.023393 |
| GO_REGULATION_OF_STEROL_TRANSPORT | 37 | 1.669085 | 0.007194 | 0.023386 |
| GO_REGULATION_OF_MUSCLE_CELL_APOPTOTIC_PROCESS | 42 | 1.668958 | 0.011111 | 0.023378 |
| GO_CARDIAC_MUSCLE_TISSUE_DEVELOPMENT | 134 | 1.667604 | 0.001704 | 0.023635 |
| GO_POSITIVE_REGULATION_OF_HOMEOSTATIC_PROCESS | 197 | 1.667277 | 0 | 0.02368 |
| GO_REGULATION_OF_PROTEIN_SERINE_THREONINE_KINASE_ACTIVITY | 448 | 1.66723 | 0 | 0.023655 |
| GO_POSITIVE_REGULATION_OF_INNATE_IMMUNE_RESPONSE | 235 | 1.666997 | 0 | 0.023687 |
| GO_NEGATIVE_REGULATION_OF_CELL_DEVELOPMENT | 290 | 1.666632 | 0 | 0.023739 |
| GO_REGULATION_OF_CAMP_METABOLIC_PROCESS | 122 | 1.666629 | 0.004983 | 0.023709 |
| GO_KERATAN_SULFATE_BIOSYNTHETIC_PROCESS | 28 | 1.66613 | 0.016293 | 0.023775 |
| GO_SENSORY_PERCEPTION_OF_PAIN | 73 | 1.665318 | 0 | 0.023929 |
| GO_CELLULAR_RESPONSE_TO_NUTRIENT | 38 | 1.665178 | 0.009671 | 0.023925 |
| GO_POSITIVE_REGULATION_OF_OSTEOCLAST_DIFFERENTIATION | 23 | 1.665031 | 0.015038 | 0.023924 |
| GO_NEGATIVE_REGULATION_OF_LYASE_ACTIVITY | 26 | 1.664829 | 0.016129 | 0.023933 |
| GO_MESENCHYMAL_CELL_DIFFERENTIATION | 132 | 1.664082 | 0 | 0.024083 |
| GO_CELLULAR_RESPONSE_TO_INORGANIC_SUBSTANCE | 146 | 1.662688 | 0 | 0.024388 |
| GO_SKELETAL_SYSTEM_DEVELOPMENT | 440 | 1.662347 | 0 | 0.02444 |
| GO_CARDIAC_MUSCLE_CELL_DIFFERENTIATION | 71 | 1.661804 | 0.003704 | 0.024524 |
| GO_REGULATION_OF_TRIGLYCERIDE_METABOLIC_PROCESS | 33 | 1.661504 | 0.009542 | 0.024561 |
| GO_REGULATION_OF_NUCLEOTIDE_METABOLIC_PROCESS | 201 | 1.661111 | 0 | 0.024626 |
| GO_REGULATION_OF_INTERLEUKIN_2_BIOSYNTHETIC_PROCESS | 17 | 1.660194 | 0.020964 | 0.02481 |
| GO_POSITIVE_REGULATION_OF_SMALL_GTPASE_MEDIATED_SIGNAL_TRANSDUCTION | 39 | 1.659148 | 0.007937 | 0.024954 |
| GO_POSITIVE_REGULATION_OF_ACTIN_FILAMENT_BUNDLE_ASSEMBLY | 46 | 1.658012 | 0.00565 | 0.025179 |
| GO_REGULATION_OF_RESPONSE_TO_REACTIVE_OXYGEN_SPECIES | 33 | 1.657572 | 0.011811 | 0.025246 |
| GO_RESPONSE_TO_CALCIUM_ION | 108 | 1.657398 | 0.001678 | 0.025263 |
| GO_POSITIVE_REGULATION_OF_PEPTIDYL_SERINE_PHOSPHORYLATION | 84 | 1.657361 | 0.003546 | 0.025243 |
| GO_NEPHRON_DEVELOPMENT | 114 | 1.656281 | 0.001742 | 0.02546 |
| GO_NEGATIVE_REGULATION_OF_DEFENSE_RESPONSE | 134 | 1.655951 | 0.001669 | 0.025496 |
| GO_CARDIOCYTE_DIFFERENTIATION | 91 | 1.655656 | 0.003273 | 0.025537 |
| GO_REGULATION_OF_INSULIN_LIKE_GROWTH_FACTOR_RECEPTOR_SIGNALING_PATHWAY | 22 | 1.655438 | 0.009634 | 0.025547 |
| GO_POSITIVE_REGULATION_OF_ESTABLISHMENT_OF_PROTEIN_LOCALIZATION | 473 | 1.655145 | 0 | 0.025578 |
| GO_REGULATION_OF_LYMPHOCYTE_CHEMOTAXIS | 17 | 1.654769 | 0.024952 | 0.025626 |
| GO_CARBOHYDRATE_DERIVATIVE_CATABOLIC_PROCESS | 164 | 1.654507 | 0 | 0.025641 |
| GO_CHONDROITIN_SULFATE_PROTEOGLYCAN_METABOLIC_PROCESS | 42 | 1.650829 | 0.009191 | 0.026414 |
| GO_CARDIAC_CHAMBER_MORPHOGENESIS | 102 | 1.649622 | 0.001626 | 0.026701 |
| GO_EMBRYONIC_DIGESTIVE_TRACT_DEVELOPMENT | 32 | 1.649363 | 0.011538 | 0.026734 |
| GO_REGULATION_OF_JUN_KINASE_ACTIVITY | 79 | 1.648043 | 0 | 0.027021 |
| GO_REGULATION_OF_MULTICELLULAR_ORGANISMAL_METABOLIC_PROCESS | 35 | 1.647694 | 0.014363 | 0.027057 |
| GO_NEGATIVE_REGULATION_OF_AXONOGENESIS | 61 | 1.647317 | 0.003643 | 0.027124 |
| GO_ALPHA_BETA_T_CELL_ACTIVATION | 53 | 1.647056 | 0.003584 | 0.027151 |
| GO_RESPONSE_TO_PH | 35 | 1.646725 | 0.013944 | 0.027186 |
| GO_REGULATION_OF_SEQUENCE_SPECIFIC_DNA_BINDING_TRANSCRIPTION_FACTOR_ACTIVITY | 346 | 1.645034 | 0 | 0.027552 |
| GO_POSITIVE_REGULATION_OF_TOLL_LIKE_RECEPTOR_SIGNALING_PATHWAY | 18 | 1.644996 | 0.021318 | 0.02753 |
| GO_CELLULAR_RESPONSE_TO_AMINO_ACID_STIMULUS | 48 | 1.644353 | 0.005576 | 0.027634 |
| GO_ACTIVATION_OF_INNATE_IMMUNE_RESPONSE | 193 | 1.644105 | 0 | 0.027667 |
| GO_REGULATION_OF_PEPTIDE_TRANSPORT | 242 | 1.642817 | 0 | 0.027949 |
| GO_BONE_MINERALIZATION | 34 | 1.64244 | 0.013109 | 0.02801 |
| GO_NEGATIVE_REGULATION_OF_PRODUCTION_OF_MOLECULAR_MEDIATOR_OF_IMMUNE_RESPONSE | 29 | 1.642059 | 0.016423 | 0.028076 |
| GO_NEGATIVE_REGULATION_OF_CD4_POSITIVE_ALPHA_BETA_T_CELL_ACTIVATION | 15 | 1.641764 | 0.020202 | 0.028106 |
| GO_CAMP_BIOSYNTHETIC_PROCESS | 16 | 1.641659 | 0.015968 | 0.028101 |
| GO_POSITIVE_REGULATION_OF_GLUCOSE_TRANSPORT | 40 | 1.641561 | 0.008818 | 0.028095 |
| GO_REGULATION_OF_LIPOPOLYSACCHARIDE_MEDIATED_SIGNALING_PATHWAY | 18 | 1.641412 | 0.019763 | 0.028108 |
| GO_NEGATIVE_REGULATION_OF_ORGANIC_ACID_TRANSPORT | 17 | 1.641244 | 0.017893 | 0.028115 |
| GO_REGULATION_OF_ACTIN_FILAMENT_BUNDLE_ASSEMBLY | 75 | 1.64114 | 0.001795 | 0.028106 |
| GO_DEFENSE_RESPONSE_TO_GRAM_NEGATIVE_BACTERIUM | 42 | 1.641129 | 0.009208 | 0.028072 |
| GO_REGULATION_OF_BEHAVIOR | 62 | 1.638124 | 0.003604 | 0.028779 |
| GO_NEGATIVE_REGULATION_OF_CARTILAGE_DEVELOPMENT | 24 | 1.637323 | 0.018036 | 0.028966 |
| GO_NEGATIVE_REGULATION_OF_CELL_MORPHOGENESIS_INVOLVED_IN_DIFFERENTIATION | 111 | 1.637026 | 0.005008 | 0.029 |
| GO_REGULATION_OF_CELLULAR_RESPONSE_TO_TRANSFORMING_GROWTH_FACTOR_BETA_STIMULUS | 94 | 1.636635 | 0.003317 | 0.029069 |
| GO_POSITIVE_REGULATION_OF_SEQUENCE_SPECIFIC_DNA_BINDING_TRANSCRIPTION_FACTOR_ACTIVITY | 218 | 1.63594 | 0 | 0.029199 |
| GO_BONE_REMODELING | 35 | 1.635549 | 0.009653 | 0.029256 |
| GO_HEART_PROCESS | 83 | 1.634086 | 0.006723 | 0.02963 |
| GO_REGULATION_OF_VASODILATION | 46 | 1.633964 | 0.009579 | 0.029631 |
| GO_OVULATION_CYCLE | 109 | 1.63377 | 0 | 0.029649 |
| GO_DEVELOPMENTAL_PIGMENTATION | 38 | 1.632573 | 0.007533 | 0.029922 |
| GO_REGULATION_OF_TYROSINE_PHOSPHORYLATION_OF_STAT5_PROTEIN | 20 | 1.632105 | 0.023622 | 0.029996 |
| GO_CELL_MORPHOGENESIS_INVOLVED_IN_NEURON_DIFFERENTIATION | 347 | 1.632009 | 0.001431 | 0.029978 |
| GO_ORGAN_GROWTH | 65 | 1.630829 | 0.008818 | 0.030242 |
| GO_POSITIVE_REGULATION_OF_CARTILAGE_DEVELOPMENT | 27 | 1.630707 | 0.01912 | 0.030232 |
| GO_REGULATION_OF_SMAD_PROTEIN_IMPORT_INTO_NUCLEUS | 15 | 1.63069 | 0.031558 | 0.0302 |
| GO_REGULATION_OF_HORMONE_SECRETION | 250 | 1.62985 | 0 | 0.030362 |
| GO_FATTY_ACID_METABOLIC_PROCESS | 272 | 1.62955 | 0.001497 | 0.030398 |
| GO_REGENERATION | 153 | 1.629043 | 0.001669 | 0.030492 |
| GO_CORONARY_VASCULATURE_DEVELOPMENT | 36 | 1.628578 | 0.016667 | 0.030554 |
| GO_RESPONSE_TO_CAFFEINE | 18 | 1.62831 | 0.01378 | 0.030575 |
| GO_NEGATIVE_REGULATION_OF_AXON_GUIDANCE | 25 | 1.628275 | 0.005792 | 0.030548 |
| GO_REGULATION_OF_NEUROTRANSMITTER_UPTAKE | 15 | 1.627157 | 0.036585 | 0.030772 |
| GO_POSITIVE_REGULATION_OF_LYMPHOCYTE_MEDIATED_IMMUNITY | 66 | 1.626271 | 0.001678 | 0.030926 |
| GO_LIPID_LOCALIZATION | 250 | 1.625499 | 0 | 0.031098 |
| GO_RESPONSE_TO_MANGANESE_ION | 16 | 1.625183 | 0.025896 | 0.031147 |
| GO_CELLULAR_LIPID_CATABOLIC_PROCESS | 142 | 1.623351 | 0 | 0.031633 |
| GO_REGULATION_OF_MYOBLAST_DIFFERENTIATION | 46 | 1.622459 | 0.003724 | 0.031857 |
| GO_REGULATION_OF_T_CELL_MEDIATED_CYTOTOXICITY | 23 | 1.622317 | 0.017274 | 0.031858 |
| GO_REGULATION_OF_RESPIRATORY_SYSTEM_PROCESS | 15 | 1.62191 | 0.02444 | 0.031926 |
| GO_MONOCARBOXYLIC_ACID_BIOSYNTHETIC_PROCESS | 158 | 1.621673 | 0.00318 | 0.031948 |
| GO_ENDOCRINE_PROCESS | 44 | 1.62104 | 0.016453 | 0.032103 |
| GO_REGULATION_OF_INTRACELLULAR_PROTEIN_TRANSPORT | 351 | 1.620343 | 0 | 0.032287 |
| GO_RESPONSE_TO_TUMOR_NECROSIS_FACTOR | 209 | 1.619027 | 0 | 0.032615 |
| GO_STAT_CASCADE | 46 | 1.61899 | 0.001802 | 0.032586 |
| GO_GLIAL_CELL_DEVELOPMENT | 73 | 1.61843 | 0.007105 | 0.032723 |
| GO_DEFENSE_RESPONSE_TO_OTHER_ORGANISM | 431 | 1.617794 | 0 | 0.032868 |
| GO_OLFACTORY_LOBE_DEVELOPMENT | 34 | 1.617656 | 0.013436 | 0.032867 |
| GO_REGULATION_OF_CELLULAR_COMPONENT_SIZE | 318 | 1.617583 | 0 | 0.032855 |
| GO_NEGATIVE_REGULATION_OF_NEURON_DEATH | 162 | 1.617459 | 0.001618 | 0.032853 |
| GO_REGULATION_OF_HEART_CONTRACTION | 215 | 1.617043 | 0 | 0.03293 |
| GO_CELL_CELL_ADHESION_VIA_PLASMA_MEMBRANE_ADHESION_MOLECULES | 172 | 1.616903 | 0 | 0.032932 |
| GO_POSITIVE_REGULATION_OF_PEPTIDE_SECRETION | 88 | 1.61617 | 0.006873 | 0.0331 |
| GO_REGULATION_OF_INTERLEUKIN_6_BIOSYNTHETIC_PROCESS | 15 | 1.615726 | 0.024845 | 0.033183 |
| GO_NEGATIVE_REGULATION_OF_REACTIVE_OXYGEN_SPECIES_METABOLIC_PROCESS | 41 | 1.615587 | 0.007605 | 0.033181 |
| GO_PROTEOGLYCAN_METABOLIC_PROCESS | 82 | 1.613563 | 0.001686 | 0.03371 |
| GO_MEMBRANE_LIPID_CATABOLIC_PROCESS | 22 | 1.613268 | 0.034026 | 0.033747 |
| GO_TRANSMEMBRANE_RECEPTOR_PROTEIN_TYROSINE_KINASE_SIGNALING_PATHWAY | 483 | 1.612189 | 0 | 0.034025 |
| GO_ACTIN_FILAMENT_BASED_PROCESS | 431 | 1.611703 | 0 | 0.03411 |
| GO_NEGATIVE_REGULATION_OF_HYDROLASE_ACTIVITY | 367 | 1.610158 | 0 | 0.034471 |
| GO_RESPONSE_TO_CAMP | 101 | 1.609411 | 0.003361 | 0.034658 |
| GO_OUTFLOW_TRACT_MORPHOGENESIS | 56 | 1.609326 | 0.007105 | 0.034642 |
| GO_STEROL_HOMEOSTASIS | 56 | 1.609247 | 0.010714 | 0.034612 |
| GO_NEGATIVE_REGULATION_OF_PEPTIDYL_TYROSINE_PHOSPHORYLATION | 37 | 1.608899 | 0.015296 | 0.034674 |
| GO_INTERLEUKIN_1_PRODUCTION | 15 | 1.608617 | 0.031447 | 0.034709 |
| GO_NEGATIVE_REGULATION_OF_EXTRINSIC_APOPTOTIC_SIGNALING_PATHWAY | 93 | 1.60805 | 0.003472 | 0.034857 |
| GO_NEGATIVE_REGULATION_OF_CALCIUM_ION_IMPORT | 20 | 1.606856 | 0.017647 | 0.035189 |
| GO_REGULATION_OF_INTRINSIC_APOPTOTIC_SIGNALING_PATHWAY_IN_RESPONSE_TO_DNA_DAMAGE | 30 | 1.606577 | 0.021782 | 0.035224 |
| GO_NEGATIVE_REGULATION_OF_CELL_CELL_ADHESION | 130 | 1.605637 | 0.001656 | 0.035458 |
| GO_NEGATIVE_REGULATION_OF_PROTEIN_MATURATION | 32 | 1.604575 | 0.013084 | 0.035723 |
| GO_POST_EMBRYONIC_DEVELOPMENT | 86 | 1.604529 | 0.015437 | 0.035692 |
| GO_DERMATAN_SULFATE_PROTEOGLYCAN_METABOLIC_PROCESS | 16 | 1.604283 | 0.025424 | 0.03571 |
| GO_TERPENOID_METABOLIC_PROCESS | 94 | 1.602401 | 0.001767 | 0.036218 |
| GO_MORPHOGENESIS_OF_A_BRANCHING_STRUCTURE | 163 | 1.60143 | 0 | 0.036452 |
| GO_DETECTION_OF_OTHER_ORGANISM | 16 | 1.600693 | 0.030426 | 0.036611 |
| GO_RESPONSE_TO_ESTROGEN | 211 | 1.600445 | 0 | 0.036638 |
| GO_POSITIVE_REGULATION_OF_CELLULAR_RESPONSE_TO_TRANSFORMING_GROWTH_FACTOR_BETA_STIMULUS | 24 | 1.600263 | 0.027132 | 0.036647 |
| GO_REGULATION_OF_JNK_CASCADE | 155 | 1.60022 | 0 | 0.036613 |
| GO_MONOCARBOXYLIC_ACID_METABOLIC_PROCESS | 457 | 1.600027 | 0 | 0.036625 |
| GO_LEUKOCYTE_HOMEOSTASIS | 59 | 1.599865 | 0.008961 | 0.036626 |
| GO_POSITIVE_REGULATION_OF_CELLULAR_COMPONENT_BIOGENESIS | 380 | 1.599241 | 0 | 0.036778 |
| GO_NEGATIVE_REGULATION_OF_OSTEOCLAST_DIFFERENTIATION | 23 | 1.599028 | 0.023952 | 0.036799 |
| GO_HYALURONAN_METABOLIC_PROCESS | 29 | 1.598244 | 0.014981 | 0.036979 |
| GO_FC_GAMMA_RECEPTOR_SIGNALING_PATHWAY | 75 | 1.597676 | 0.005102 | 0.037096 |
| GO_RESPONSE_TO_ESTRADIOL | 144 | 1.597561 | 0.001661 | 0.037094 |
| GO_NEGATIVE_REGULATION_OF_INTRACELLULAR_SIGNAL_TRANSDUCTION | 408 | 1.597397 | 0 | 0.037102 |
| GO_ORGAN_MATURATION | 18 | 1.597292 | 0.031621 | 0.037098 |
| GO_REGULATION_OF_EXTENT_OF_CELL_GROWTH | 97 | 1.597097 | 0.001672 | 0.037108 |
| GO_SMOOTH_MUSCLE_CELL_DIFFERENTIATION | 30 | 1.596747 | 0.032319 | 0.037188 |
| GO_MYOFIBRIL_ASSEMBLY | 45 | 1.59673 | 0.019342 | 0.037151 |
| GO_INOSITOL_LIPID_MEDIATED_SIGNALING | 115 | 1.595608 | 0.003115 | 0.037423 |
| GO_PRODUCTION_OF_MOLECULAR_MEDIATOR_OF_IMMUNE_RESPONSE | 58 | 1.594783 | 0.010508 | 0.037657 |
| GO_POSITIVE_REGULATION_OF_AMINE_TRANSPORT | 32 | 1.594558 | 0.02439 | 0.037688 |
| GO_POSITIVE_REGULATION_OF_EXTRINSIC_APOPTOTIC_SIGNALING_PATHWAY | 50 | 1.593618 | 0.009259 | 0.037947 |
| GO_RESPONSE_TO_INCREASED_OXYGEN_LEVELS | 23 | 1.593144 | 0.011905 | 0.038057 |
| GO_REGULATION_OF_TISSUE_REMODELING | 58 | 1.592952 | 0.013035 | 0.038081 |
| GO_GLUCOCORTICOID_METABOLIC_PROCESS | 15 | 1.592781 | 0.02729 | 0.038081 |
| GO_MELANOCYTE_DIFFERENTIATION | 19 | 1.592362 | 0.024482 | 0.038176 |
| GO_CALCIUM_DEPENDENT_CELL_CELL_ADHESION_VIA_PLASMA_MEMBRANE_CELL_ADHESION_MOLECULES | 23 | 1.592234 | 0.029183 | 0.038166 |
| GO_POSITIVE_REGULATION_OF_MUSCLE_TISSUE_DEVELOPMENT | 54 | 1.592004 | 0.014981 | 0.038195 |
| GO_REGULATION_OF_PROTEIN_POLYMERIZATION | 164 | 1.591588 | 0 | 0.038278 |
| GO_POSITIVE_REGULATION_OF_PROTEIN_LOCALIZATION_TO_NUCLEUS | 122 | 1.591587 | 0.004823 | 0.038235 |
| GO_CAMP_METABOLIC_PROCESS | 32 | 1.591097 | 0.02008 | 0.038345 |
| GO_NEGATIVE_REGULATION_OF_LEUKOCYTE_MEDIATED_IMMUNITY | 46 | 1.59096 | 0.009381 | 0.038358 |
| GO_REGULATION_OF_INFLAMMATORY_RESPONSE_TO_ANTIGENIC_STIMULUS | 17 | 1.590716 | 0.024742 | 0.038379 |
| GO_MUSCLE_CELL_DEVELOPMENT | 120 | 1.590195 | 0.004926 | 0.038487 |
| GO_POSITIVE_REGULATION_OF_HEART_CONTRACTION | 35 | 1.589395 | 0.016423 | 0.03867 |
| GO_ENSHEATHMENT_OF_NEURONS | 87 | 1.58895 | 0 | 0.038757 |
| GO_POSITIVE_REGULATION_OF_BIOMINERAL_TISSUE_DEVELOPMENT | 36 | 1.588622 | 0.015534 | 0.038809 |
| GO_RESPONSE_TO_DEXAMETHASONE | 33 | 1.587846 | 0.022642 | 0.039016 |
| GO_ENDOCARDIAL_CUSHION_MORPHOGENESIS | 22 | 1.584907 | 0.028736 | 0.039879 |
| GO_CARDIAC_SEPTUM_DEVELOPMENT | 84 | 1.58482 | 0.005236 | 0.039869 |
| GO_POSITIVE_REGULATION_OF_NUCLEOTIDE_METABOLIC_PROCESS | 126 | 1.584397 | 0.003295 | 0.039968 |
| GO_CELL_CELL_SIGNALING_INVOLVED_IN_CARDIAC_CONDUCTION | 22 | 1.584054 | 0.018145 | 0.040056 |
| GO_INTERACTION_WITH_SYMBIONT | 50 | 1.583543 | 0.010733 | 0.04019 |
| GO_NEGATIVE_REGULATION_OF_NEURON_DIFFERENTIATION | 184 | 1.58354 | 0 | 0.040147 |
| GO_POSITIVE_REGULATION_OF_STRESS_ACTIVATED_PROTEIN_KINASE_SIGNALING_CASCADE | 130 | 1.583277 | 0.001698 | 0.040188 |
| GO_NEGATIVE_REGULATION_OF_GROWTH | 226 | 1.58314 | 0 | 0.040202 |
| GO_RESPONSE_TO_ANTIBIOTIC | 45 | 1.582658 | 0.012891 | 0.040307 |
| GO_SEGMENT_SPECIFICATION | 15 | 1.582184 | 0.024948 | 0.040429 |
| GO_NEGATIVE_REGULATION_OF_RESPONSE_TO_REACTIVE_OXYGEN_SPECIES | 17 | 1.581837 | 0.040426 | 0.040498 |
| GO_RHO_PROTEIN_SIGNAL_TRANSDUCTION | 48 | 1.581703 | 0.010695 | 0.040491 |
| GO_REGULATION_OF_MEMBRANE_REPOLARIZATION | 30 | 1.581555 | 0.022 | 0.040491 |
| GO_SYNAPSE_ASSEMBLY | 65 | 1.581534 | 0.010969 | 0.040449 |
| GO_REGULATION_OF_SMOOTHENED_SIGNALING_PATHWAY | 61 | 1.581391 | 0.017606 | 0.040454 |
| GO_REGULATION_OF_CYTOPLASMIC_TRANSPORT | 453 | 1.580653 | 0 | 0.040645 |
| GO_POSITIVE_REGULATION_OF_OXIDOREDUCTASE_ACTIVITY | 43 | 1.580646 | 0.013333 | 0.0406 |
| GO_REGULATION_OF_ADENYLATE_CYCLASE_ACTIVITY | 64 | 1.580011 | 0.010204 | 0.040768 |
| GO_NEURAL_CREST_CELL_DIFFERENTIATION | 74 | 1.579598 | 0.012367 | 0.040854 |
| GO_MESENCHYME_MORPHOGENESIS | 37 | 1.579186 | 0.019298 | 0.040964 |
| GO_NEGATIVE_REGULATION_OF_MITOCHONDRION_ORGANIZATION | 36 | 1.578898 | 0.021484 | 0.041017 |
| GO_RESPONSE_TO_NUTRIENT | 189 | 1.577957 | 0.001623 | 0.041315 |
| GO_T_CELL_ACTIVATION_INVOLVED_IN_IMMUNE_RESPONSE | 57 | 1.57739 | 0.005474 | 0.041468 |
| GO_POSITIVE_REGULATION_OF_CHONDROCYTE_DIFFERENTIATION | 18 | 1.577137 | 0.028926 | 0.041509 |
| GO_MODULATION_BY_HOST_OF_VIRAL_PROCESS | 17 | 1.576835 | 0.042918 | 0.041553 |
| GO_CELLULAR_GLUCOSE_HOMEOSTASIS | 73 | 1.576391 | 0.00885 | 0.04167 |
| GO_POSITIVE_REGULATION_OF_VASCULAR_ENDOTHELIAL_GROWTH_FACTOR_RECEPTOR_SIGNALING_PATHWAY | 15 | 1.575377 | 0.041502 | 0.04199 |
| GO_ENDOCARDIAL_CUSHION_DEVELOPMENT | 32 | 1.575134 | 0.015717 | 0.042031 |
| GO_NEGATIVE_REGULATION_OF_ALCOHOL_BIOSYNTHETIC_PROCESS | 17 | 1.574958 | 0.0369 | 0.042034 |
| GO_CARDIAC_EPITHELIAL_TO_MESENCHYMAL_TRANSITION | 24 | 1.573958 | 0.043307 | 0.042346 |
| GO_I_KAPPAB_KINASE_NF_KAPPAB_SIGNALING | 67 | 1.573894 | 0.010363 | 0.042326 |
| GO_REGULATION_OF_B_CELL_MEDIATED_IMMUNITY | 41 | 1.573225 | 0.016886 | 0.042474 |
| GO_POSITIVE_REGULATION_OF_JUN_KINASE_ACTIVITY | 61 | 1.572722 | 0.009042 | 0.042589 |
| GO_ADRENERGIC_RECEPTOR_SIGNALING_PATHWAY | 18 | 1.571684 | 0.021442 | 0.042921 |
| GO_ORGANOPHOSPHATE_ESTER_TRANSPORT | 85 | 1.571499 | 0.010435 | 0.042963 |
| GO_ASTROCYTE_DIFFERENTIATION | 39 | 1.571197 | 0.020446 | 0.043045 |
| GO_CARDIAC_VENTRICLE_DEVELOPMENT | 104 | 1.571099 | 0.008475 | 0.04303 |
| GO_HOMEOSTASIS_OF_NUMBER_OF_CELLS | 169 | 1.570887 | 0 | 0.043047 |
| GO_REGULATION_OF_CELL_SIZE | 163 | 1.570553 | 0.003215 | 0.043133 |
| GO_POSITIVE_REGULATION_OF_CYTOPLASMIC_TRANSPORT | 263 | 1.570174 | 0 | 0.043244 |
| GO_POSITIVE_REGULATION_OF_PRODUCTION_OF_MOLECULAR_MEDIATOR_OF_IMMUNE_RESPONSE | 60 | 1.570114 | 0.017762 | 0.043213 |
| GO_T_CELL_DIFFERENTIATION_INVOLVED_IN_IMMUNE_RESPONSE | 28 | 1.56996 | 0.026923 | 0.043231 |
| GO_ARTERY_MORPHOGENESIS | 51 | 1.569044 | 0.013133 | 0.043459 |
| GO_REGULATION_OF_INNATE_IMMUNE_RESPONSE | 337 | 1.569009 | 0 | 0.043428 |
| GO_ENDOTHELIAL_CELL_DEVELOPMENT | 45 | 1.568682 | 0.023297 | 0.043494 |
| GO_EMBRYO_IMPLANTATION | 36 | 1.567843 | 0.019305 | 0.043751 |
| GO_REGULATION_OF_RESPONSE_TO_OXIDATIVE_STRESS | 62 | 1.567408 | 0.015929 | 0.043859 |
| GO_VENTRICULAR_SEPTUM_DEVELOPMENT | 54 | 1.566947 | 0.01354 | 0.043971 |
| GO_REGULATION_OF_INTERFERON_GAMMA_BIOSYNTHETIC_PROCESS | 16 | 1.566792 | 0.025586 | 0.043974 |
| GO_MODULATION_OF_GROWTH_OF_SYMBIONT_INVOLVED_IN_INTERACTION_WITH_HOST | 16 | 1.566733 | 0.045098 | 0.043945 |
| GO_POSITIVE_REGULATION_OF_EPITHELIAL_CELL_APOPTOTIC_PROCESS | 22 | 1.565461 | 0.035714 | 0.0443 |
| GO_CYCLIC_NUCLEOTIDE_CATABOLIC_PROCESS | 16 | 1.565415 | 0.041916 | 0.044266 |
| GO_CELLULAR_RESPONSE_TO_OSMOTIC_STRESS | 20 | 1.565391 | 0.045455 | 0.044232 |
| GO_NEGATIVE_REGULATION_OF_REPRODUCTIVE_PROCESS | 47 | 1.562963 | 0.014981 | 0.045052 |
| GO_GLIOGENESIS | 168 | 1.562848 | 0.003466 | 0.045056 |
| GO_CELLULAR_RESPONSE_TO_HYDROGEN_PEROXIDE | 60 | 1.562795 | 0.01406 | 0.045018 |
| GO_APOPTOTIC_PROCESS_INVOLVED_IN_DEVELOPMENT | 20 | 1.561847 | 0.04175 | 0.045276 |
| GO_REGULATION_OF_CELL_GROWTH | 370 | 1.561578 | 0 | 0.045338 |
| GO_POSITIVE_REGULATION_OF_SODIUM_ION_TRANSPORT | 31 | 1.560428 | 0.026465 | 0.045753 |
| GO_RESPONSE_TO_MUSCLE_ACTIVITY | 19 | 1.560379 | 0.031683 | 0.045706 |
| GO_TRANSFORMING_GROWTH_FACTOR_BETA_RECEPTOR_SIGNALING_PATHWAY | 92 | 1.560248 | 0.005025 | 0.045714 |
| GO_REGULATION_OF_REPRODUCTIVE_PROCESS | 119 | 1.560122 | 0.001664 | 0.045712 |
| GO_REGULATION_OF_CYTOKINE_PRODUCTION_INVOLVED_IN_INFLAMMATORY_RESPONSE | 16 | 1.5601 | 0.033473 | 0.045673 |
| GO_LYMPH_NODE_DEVELOPMENT | 17 | 1.55929 | 0.03012 | 0.045936 |
| GO_REGULATION_OF_EXTRINSIC_APOPTOTIC_SIGNALING_PATHWAY_IN_ABSENCE_OF_LIGAND | 43 | 1.557414 | 0.018762 | 0.04663 |
| GO_REGULATION_OF_INTERLEUKIN_1_SECRETION | 28 | 1.555021 | 0.022857 | 0.047435 |
| GO_NEGATIVE_REGULATION_OF_MYELOID_LEUKOCYTE_DIFFERENTIATION | 41 | 1.554298 | 0.016605 | 0.047656 |
| GO_CELLULAR_RESPONSE_TO_VITAMIN | 25 | 1.55411 | 0.032319 | 0.047675 |
| GO_REGULATION_OF_VIRAL_ENTRY_INTO_HOST_CELL | 28 | 1.553831 | 0.031193 | 0.047753 |
| GO_SKELETAL_MUSCLE_CELL_DIFFERENTIATION | 49 | 1.553271 | 0.01275 | 0.047897 |
| GO_REGULATION_OF_LIPID_BIOSYNTHETIC_PROCESS | 121 | 1.551758 | 0.005093 | 0.048421 |
| GO_REGULATION_OF_POTASSIUM_ION_TRANSPORT | 83 | 1.551377 | 0.001727 | 0.04854 |
| GO_SPECIFICATION_OF_SYMMETRY | 110 | 1.551214 | 0.00692 | 0.04856 |
| GO_NEGATIVE_REGULATION_OF_APOPTOTIC_SIGNALING_PATHWAY | 184 | 1.550953 | 0.004902 | 0.048623 |
| GO_POSITIVE_REGULATION_OF_DEVELOPMENTAL_GROWTH | 150 | 1.550834 | 0.006711 | 0.048616 |
| GO_MULTICELLULAR_ORGANISMAL_RESPONSE_TO_STRESS | 66 | 1.550791 | 0.015437 | 0.048587 |
| GO_ENDODERM_FORMATION | 48 | 1.550442 | 0.014599 | 0.048684 |
| GO_POSITIVE_REGULATION_OF_ADHERENS_JUNCTION_ORGANIZATION | 21 | 1.549527 | 0.038776 | 0.049031 |
| GO_NEGATIVE_REGULATION_OF_FAT_CELL_DIFFERENTIATION | 37 | 1.549452 | 0.020183 | 0.049008 |
| GO_POSITIVE_REGULATION_OF_KIDNEY_DEVELOPMENT | 41 | 1.549285 | 0.021661 | 0.049018 |
| GO_POSITIVE_REGULATION_OF_FATTY_ACID_BIOSYNTHETIC_PROCESS | 17 | 1.549239 | 0.044444 | 0.048988 |
| GO_RESPONSE_TO_SALT_STRESS | 19 | 1.54785 | 0.038776 | 0.049502 |
| GO_NEGATIVE_REGULATION_OF_CATECHOLAMINE_SECRETION | 16 | 1.547351 | 0.048 | 0.049641 |

2. GSEA for IPM-high recurrence group

| NAME | SIZE | NES | NOM p-val | FDR q-val |
| --- | --- | --- | --- | --- |
| GO_SISTER_CHROMATID_SEGREGATION | 164 | -3.00095 | 0 | 0 |
| GO_NUCLEAR_CHROMOSOME_SEGREGATION | 212 | -2.83463 | 0 | 0 |
| GO_CHROMOSOME_SEGREGATION | 255 | -2.78091 | 0 | 0 |
| GO_SISTER_CHROMATID_COHESION | 104 | -2.77957 | 0 | 0 |
| GO_MITOTIC_SISTER_CHROMATID_SEGREGATION | 85 | -2.73692 | 0 | 0 |
| GO_DNA_DEPENDENT_DNA_REPLICATION | 90 | -2.70637 | 0 | 0 |
| GO_MITOTIC_RECOMBINATION | 41 | -2.62324 | 0 | 0 |
| GO_MITOTIC_NUCLEAR_DIVISION | 336 | -2.60322 | 0 | 0 |
| GO_DNA_REPLICATION | 192 | -2.59881 | 0 | 0 |
| GO_TELOMERE_MAINTENANCE_VIA_RECOMBINATION | 32 | -2.54563 | 0 | 0 |
| GO_ORGANELLE_FISSION | 459 | -2.53697 | 0 | 0 |
| GO_CELL_CYCLE_G1_S_PHASE_TRANSITION | 107 | -2.50983 | 0 | 0 |
| GO_DNA_REPLICATION_INDEPENDENT_NUCLEOSOME_ORGANIZATION | 41 | -2.48238 | 0 | 0 |
| GO_DNA_CONFORMATION_CHANGE | 232 | -2.47698 | 0 | 0 |
| GO_CELL_CYCLE_CHECKPOINT | 183 | -2.46216 | 0 | 0 |
| GO_DNA_RECOMBINATION | 191 | -2.4546 | 0 | 0 |
| GO_REGULATION_OF_TRANSCRIPTION_INVOLVED_IN_G1_S_TRANSITION_OF_MITOTIC_CELL_CYCLE | 25 | -2.44076 | 0 | 0 |
| GO_MEIOTIC_CELL_CYCLE | 173 | -2.43808 | 0 | 0 |
| GO_DNA_REPLICATION_INITIATION | 28 | -2.43184 | 0 | 0 |
| GO_HISTONE_EXCHANGE | 42 | -2.41802 | 0 | 0 |
| GO_TELOMERE_ORGANIZATION | 88 | -2.40905 | 0 | 0 |
| GO_KERATINIZATION | 30 | -2.40763 | 0 | 0 |
| GO_CELL_CYCLE_PHASE_TRANSITION | 244 | -2.39591 | 0 | 0 |
| GO_REGULATION_OF_CHROMOSOME_SEGREGATION | 83 | -2.39459 | 0 | 0 |
| GO_CENTROMERE_COMPLEX_ASSEMBLY | 37 | -2.39452 | 0 | 0 |
| GO_CELL_DIVISION | 426 | -2.38131 | 0 | 0 |
| GO_DNA_STRAND_ELONGATION_INVOLVED_IN_DNA_REPLICATION | 25 | -2.37016 | 0 | 0 |
| GO_MEIOTIC_CELL_CYCLE_PROCESS | 142 | -2.36097 | 0 | 0 |
| GO_NEGATIVE_REGULATION_OF_CELLULAR_PROTEIN_CATABOLIC_PROCESS | 58 | -2.35169 | 0 | 0 |
| GO_STRAND_DISPLACEMENT | 26 | -2.34909 | 0 | 0 |
| GO_DOUBLE_STRAND_BREAK_REPAIR | 146 | -2.33849 | 0 | 0 |
| GO_DNA_STRAND_ELONGATION | 30 | -2.32759 | 0 | 0 |
| GO_DNA_PACKAGING | 156 | -2.31871 | 0 | 0 |
| GO_DNA_SYNTHESIS_INVOLVED_IN_DNA_REPAIR | 71 | -2.31492 | 0 | 0 |
| GO_NEGATIVE_REGULATION_OF_MITOTIC_NUCLEAR_DIVISION | 33 | -2.30299 | 0 | 0 |
| GO_REGULATION_OF_SISTER_CHROMATID_SEGREGATION | 66 | -2.28293 | 0 | 0 |
| GO_DNA_BIOSYNTHETIC_PROCESS | 111 | -2.27913 | 0 | 0 |
| GO_METAPHASE_PLATE_CONGRESSION | 39 | -2.26097 | 0 | 0 |
| GO_RECOMBINATIONAL_REPAIR | 69 | -2.26032 | 0 | 0 |
| GO_CHROMOSOME_LOCALIZATION | 58 | -2.25564 | 0 | 0 |
| GO_DNA_GEOMETRIC_CHANGE | 78 | -2.25531 | 0 | 0 |
| GO_MITOTIC_CELL_CYCLE_CHECKPOINT | 132 | -2.24564 | 0 | 1.60E-05 |
| GO_DNA_REPAIR | 431 | -2.2425 | 0 | 1.56E-05 |
| GO_TRNA_METABOLIC_PROCESS | 159 | -2.20474 | 0 | 3.06E-05 |
| GO_REGULATION_OF_NUCLEAR_DIVISION | 155 | -2.19369 | 0 | 2.99E-05 |
| GO_SPINDLE_CHECKPOINT | 24 | -2.16244 | 0 | 1.01E-04 |
| GO_DNA_INTEGRITY_CHECKPOINT | 139 | -2.15756 | 0 | 9.84E-05 |
| GO_TRNA_PROCESSING | 103 | -2.15732 | 0 | 9.64E-05 |
| GO_KERATINOCYTE_DIFFERENTIATION | 80 | -2.14752 | 0 | 1.22E-04 |
| GO_ATP_DEPENDENT_CHROMATIN_REMODELING | 63 | -2.14473 | 0 | 1.32E-04 |
| GO_ANAPHASE_PROMOTING_COMPLEX_DEPENDENT_CATABOLIC_PROCESS | 72 | -2.131 | 0 | 1.43E-04 |
| GO_NEGATIVE_REGULATION_OF_CHROMOSOME_SEGREGATION | 28 | -2.12754 | 0 | 1.78E-04 |
| GO_CHROMOSOME_CONDENSATION | 29 | -2.12348 | 0 | 1.87E-04 |
| GO_RIBONUCLEOPROTEIN_COMPLEX_LOCALIZATION | 105 | -2.10676 | 0 | 2.21E-04 |
| GO_DEOXYRIBONUCLEOTIDE_METABOLIC_PROCESS | 32 | -2.1061 | 0 | 2.29E-04 |
| GO_CHROMATIN_ASSEMBLY_OR_DISASSEMBLY | 141 | -2.10239 | 0 | 2.60E-04 |
| GO_NEGATIVE_REGULATION_OF_CELL_DIVISION | 59 | -2.09686 | 0 | 2.67E-04 |
| GO_NEGATIVE_REGULATION_OF_NUCLEAR_DIVISION | 45 | -2.09366 | 0 | 2.63E-04 |
| GO_MITOCHONDRIAL_TRANSLATION | 102 | -2.09049 | 0 | 2.70E-04 |
| GO_TRANSLATIONAL_ELONGATION | 104 | -2.08747 | 0 | 2.88E-04 |
| GO_MEIOSIS_I | 81 | -2.0849 | 0 | 2.83E-04 |
| GO_PROTEIN_LOCALIZATION_TO_CHROMOSOME | 42 | -2.07601 | 0 | 2.99E-04 |
| GO_REGULATION_OF_WATER_LOSS_VIA_SKIN | 16 | -2.06992 | 0 | 3.68E-04 |
| GO_EPIDERMAL_CELL_DIFFERENTIATION | 119 | -2.06156 | 0 | 4.03E-04 |
| GO_PROTEIN_DNA_COMPLEX_SUBUNIT_ORGANIZATION | 193 | -2.06022 | 0 | 3.97E-04 |
| GO_NON_RECOMBINATIONAL_REPAIR | 59 | -2.04522 | 0 | 5.22E-04 |
| GO_EPIDERMIS_DEVELOPMENT | 227 | -2.03549 | 0 | 6.53E-04 |
| GO_BASE_EXCISION_REPAIR | 38 | -2.0339 | 0 | 6.53E-04 |
| GO_REGULATION_OF_SIGNAL_TRANSDUCTION_BY_P53_CLASS_MEDIATOR | 158 | -2.01227 | 0 | 9.70E-04 |
| GO_REGULATION_OF_DNA_DEPENDENT_DNA_REPLICATION | 40 | -2.00853 | 0 | 0.001032 |
| GO_TRANSLATIONAL_TERMINATION | 90 | -2.00544 | 0 | 0.001064 |
| GO_REGULATION_OF_CYTOKINESIS | 56 | -2.0039 | 0 | 0.001059 |
| GO_FEMALE_MEIOTIC_DIVISION | 22 | -2.00337 | 0 | 0.001062 |
| GO_CHROMATIN_REMODELING | 137 | -2.00207 | 0 | 0.001084 |
| GO_TRNA_MODIFICATION | 53 | -1.9774 | 0 | 0.001749 |
| GO_COENZYME_BIOSYNTHETIC_PROCESS | 113 | -1.97135 | 0 | 0.001866 |
| GO_INTERSTRAND_CROSS_LINK_REPAIR | 39 | -1.96699 | 0.002174 | 0.001953 |
| GO_MITOTIC_CYTOKINESIS | 30 | -1.96634 | 0 | 0.001954 |
| GO_MEIOTIC_CHROMOSOME_SEGREGATION | 58 | -1.96163 | 0 | 0.002063 |
| GO_NCRNA_METABOLIC_PROCESS | 468 | -1.95415 | 0 | 0.002269 |
| GO_REGULATION_OF_CELL_DIVISION | 252 | -1.94971 | 0 | 0.002445 |
| GO_PYRIMIDINE_DEOXYRIBONUCLEOTIDE_METABOLIC_PROCESS | 15 | -1.94794 | 0 | 0.002513 |
| GO_MITOTIC_DNA_INTEGRITY_CHECKPOINT | 96 | -1.94489 | 0 | 0.002642 |
| GO_NEGATIVE_REGULATION_OF_MITOTIC_CELL_CYCLE | 188 | -1.94482 | 0 | 0.002611 |
| GO_REGULATION_OF_CENTROSOME_CYCLE | 36 | -1.94467 | 0 | 0.00258 |
| GO_POSITIVE_REGULATION_OF_CYTOKINESIS | 31 | -1.94465 | 0 | 0.002558 |
| GO_RNA_LOCALIZATION | 163 | -1.94112 | 0 | 0.002597 |
| GO_POSITIVE_REGULATION_OF_CHROMOSOME_SEGREGATION | 25 | -1.93771 | 0 | 0.002704 |
| GO_NUCLEOTIDE_EXCISION_REPAIR_DNA_GAP_FILLING | 24 | -1.93537 | 0 | 0.002755 |
| GO_SKIN_DEVELOPMENT | 185 | -1.93119 | 0 | 0.002873 |
| GO_REGULATION_OF_LIGASE_ACTIVITY | 121 | -1.93032 | 0 | 0.002878 |
| GO_DNA_DAMAGE_RESPONSE_DETECTION_OF_DNA_DAMAGE | 36 | -1.92837 | 0 | 0.002963 |
| GO_SPINDLE_ASSEMBLY | 66 | -1.9198 | 0 | 0.003266 |
| GO_NCRNA_PROCESSING | 337 | -1.91931 | 0 | 0.003259 |
| GO_POSITIVE_REGULATION_OF_CELL_CYCLE_PROCESS | 234 | -1.9191 | 0 | 0.003225 |
| GO_REGULATION_OF_EXIT_FROM_MITOSIS | 15 | -1.91546 | 0.002012 | 0.003364 |
| GO_RNA_MODIFICATION | 107 | -1.91341 | 0 | 0.003459 |
| GO_DEOXYRIBONUCLEOSIDE_TRIPHOSPHATE_METABOLIC_PROCESS | 16 | -1.91332 | 0 | 0.003437 |
| GO_NEGATIVE_REGULATION_OF_CHROMOSOME_ORGANIZATION | 94 | -1.91055 | 0 | 0.003563 |
| GO_DNA_DAMAGE_RESPONSE_SIGNAL_TRANSDUCTION_RESULTING_IN_TRANSCRIPTION | 15 | -1.89543 | 0.001949 | 0.004437 |
| GO_COENZYME_A_METABOLIC_PROCESS | 17 | -1.89483 | 0 | 0.004433 |
| GO_DNA_REPLICATION_DEPENDENT_NUCLEOSOME_ORGANIZATION | 22 | -1.89312 | 0 | 0.004519 |
| GO_RIBOSOME_BIOGENESIS | 270 | -1.89048 | 0 | 0.004643 |
| GO_NEGATIVE_REGULATION_OF_PROTEIN_MODIFICATION_BY_SMALL_PROTEIN_CONJUGATION_OR_REMOVAL | 125 | -1.88662 | 0 | 0.004885 |
| GO_REGULATION_OF_MITOTIC_CELL_CYCLE | 443 | -1.88403 | 0 | 0.005167 |
| GO_ACTIVATION_OF_ANAPHASE_PROMOTING_COMPLEX_ACTIVITY | 15 | -1.87805 | 0 | 0.005682 |
| GO_REGULATION_OF_PROTEIN_UBIQUITINATION_INVOLVED_IN_UBIQUITIN_DEPENDENT_PROTEIN_CATABOLIC_PROCESS | 96 | -1.87497 | 0 | 0.005895 |
| GO_CELL_CYCLE_G2_M_PHASE_TRANSITION | 131 | -1.86906 | 0 | 0.006276 |
| GO_RECIPROCAL_DNA_RECOMBINATION | 36 | -1.86536 | 0 | 0.006558 |
| GO_PROTEIN_COMPLEX_LOCALIZATION | 50 | -1.86266 | 0 | 0.006717 |
| GO_DNA_DEPENDENT_DNA_REPLICATION_MAINTENANCE_OF_FIDELITY | 19 | -1.85764 | 0.001988 | 0.006978 |
| GO_RIBONUCLEOPROTEIN_COMPLEX_BIOGENESIS | 388 | -1.85284 | 0 | 0.007344 |
| GO_COFACTOR_BIOSYNTHETIC_PROCESS | 152 | -1.8519 | 0 | 0.007396 |
| GO_CYTOSKELETON_DEPENDENT_CYTOKINESIS | 38 | -1.85038 | 0 | 0.007541 |
| GO_NUCLEIC_ACID_PHOSPHODIESTER_BOND_HYDROLYSIS | 222 | -1.84506 | 0 | 0.008035 |
| GO_SIGNAL_TRANSDUCTION_IN_RESPONSE_TO_DNA_DAMAGE | 92 | -1.83113 | 0 | 0.009657 |
| GO_TELOMERE_MAINTENANCE_VIA_TELOMERE_LENGTHENING | 25 | -1.83093 | 0 | 0.009598 |
| GO_FOLIC_ACID_CONTAINING_COMPOUND_METABOLIC_PROCESS | 27 | -1.82888 | 0.002037 | 0.009696 |
| GO_FOLIC_ACID_METABOLIC_PROCESS | 16 | -1.82786 | 0.001961 | 0.00972 |
| GO_CHROMOSOME_ORGANIZATION_INVOLVED_IN_MEIOTIC_CELL_CYCLE | 46 | -1.82767 | 0 | 0.00965 |
| GO_RNA_METHYLATION | 48 | -1.82646 | 0 | 0.009746 |
| GO_PTERIDINE_CONTAINING_COMPOUND_METABOLIC_PROCESS | 33 | -1.81188 | 0.002105 | 0.011497 |
| GO_POSITIVE_REGULATION_OF_LIGASE_ACTIVITY | 101 | -1.81158 | 0 | 0.011446 |
| GO_POSTREPLICATION_REPAIR | 49 | -1.81088 | 0 | 0.011429 |
| GO_CYTOKINESIS | 79 | -1.81062 | 0.002457 | 0.011379 |
| GO_CLEAVAGE_INVOLVED_IN_RRNA_PROCESSING | 17 | -1.80553 | 0 | 0.012034 |
| GO_DEOXYRIBOSE_PHOSPHATE_CATABOLIC_PROCESS | 20 | -1.80205 | 0.002058 | 0.012456 |
| GO_G1_DNA_DAMAGE_CHECKPOINT | 70 | -1.79991 | 0 | 0.012721 |
| GO_POSITIVE_REGULATION_OF_CELL_CYCLE | 313 | -1.79666 | 0 | 0.013073 |
| GO_TRNA_TRANSPORT | 32 | -1.79579 | 0 | 0.013094 |
| GO_PYRIMIDINE_NUCLEOTIDE_METABOLIC_PROCESS | 40 | -1.79562 | 0.002141 | 0.013035 |
| GO_PREASSEMBLY_OF_GPI_ANCHOR_IN_ER_MEMBRANE | 15 | -1.79555 | 0.001938 | 0.012951 |
| GO_NEGATIVE_REGULATION_OF_DNA_REPLICATION | 53 | -1.79418 | 0 | 0.013109 |
| GO_NUCLEOBASE_CONTAINING_COMPOUND_TRANSPORT | 177 | -1.78635 | 0 | 0.01415 |
| GO_POSITIVE_REGULATION_OF_CELL_CYCLE_PHASE_TRANSITION | 67 | -1.78546 | 0 | 0.014187 |
| GO_MEMBRANE_DISASSEMBLY | 45 | -1.78393 | 0 | 0.014307 |
| GO_MITOTIC_SPINDLE_ORGANIZATION | 64 | -1.78367 | 0 | 0.014207 |
| GO_NUCLEAR_EXPORT | 130 | -1.78283 | 0 | 0.014229 |
| GO_REGULATION_OF_TELOMERE_MAINTENANCE_VIA_TELOMERE_LENGTHENING | 46 | -1.76686 | 0.002137 | 0.016918 |
| GO_MITOCHONDRIAL_RNA_METABOLIC_PROCESS | 23 | -1.75795 | 0.00198 | 0.018351 |
| GO_ESTABLISHMENT_OF_MITOTIC_SPINDLE_LOCALIZATION | 23 | -1.75566 | 0.006048 | 0.018646 |
| GO_PROTEIN_HETEROTETRAMERIZATION | 28 | -1.75086 | 0.006303 | 0.019607 |
| GO_POSITIVE_REGULATION_OF_MITOTIC_CELL_CYCLE | 120 | -1.74775 | 0 | 0.020156 |
| GO_CENTROSOME_CYCLE | 45 | -1.74613 | 0 | 0.020361 |
| GO_DNA_DOUBLE_STRAND_BREAK_PROCESSING | 18 | -1.74323 | 0.008316 | 0.020971 |
| GO_RNA_PHOSPHODIESTER_BOND_HYDROLYSIS | 101 | -1.73905 | 0 | 0.021713 |
| GO_MATURATION_OF_5_8S_RRNA | 26 | -1.73745 | 0.006024 | 0.021962 |
| GO_REGULATION_OF_CELL_CYCLE_PHASE_TRANSITION | 308 | -1.73717 | 0 | 0.021872 |
| GO_MICROTUBULE_ORGANIZING_CENTER_ORGANIZATION | 81 | -1.73514 | 0 | 0.022242 |
| GO_G2_DNA_DAMAGE_CHECKPOINT | 33 | -1.73423 | 0 | 0.022279 |
| GO_CELLULAR_PROTEIN_COMPLEX_DISASSEMBLY | 118 | -1.73383 | 0 | 0.02221 |
| GO_ERROR_FREE_TRANSLESION_SYNTHESIS | 18 | -1.73345 | 0.004141 | 0.022138 |
| GO_GLUTAMATE_METABOLIC_PROCESS | 26 | -1.73303 | 0.006452 | 0.022071 |
| GO_NUCLEAR_TRANSCRIBED_MRNA_CATABOLIC_PROCESS_EXONUCLEOLYTIC | 30 | -1.73107 | 0.001992 | 0.02241 |
| GO_GLUTAMINE_FAMILY_AMINO_ACID_CATABOLIC_PROCESS | 23 | -1.72718 | 0.002088 | 0.023175 |
| GO_MATURATION_OF_5_8S_RRNA_FROM_TRICISTRONIC_RRNA_TRANSCRIPT_SSU_RRNA_5_8S_RRNA_LSU_RRNA_ | 17 | -1.72693 | 0.003945 | 0.023112 |
| GO_NUCLEOTIDE_EXCISION_REPAIR | 107 | -1.71719 | 0 | 0.0255 |
| GO_NEGATIVE_REGULATION_OF_CELL_CYCLE_PROCESS | 204 | -1.71695 | 0 | 0.02541 |
| GO_SPINDLE_LOCALIZATION | 37 | -1.71503 | 0 | 0.025768 |
| GO_NUCLEAR_ENVELOPE_ORGANIZATION | 77 | -1.70987 | 0 | 0.026962 |
| GO_RRNA_METABOLIC_PROCESS | 221 | -1.70969 | 0 | 0.026865 |
| GO_SPLICEOSOMAL_SNRNP_ASSEMBLY | 35 | -1.70904 | 0 | 0.026834 |
| GO_REGULATION_OF_MICROTUBULE_BASED_PROCESS | 223 | -1.70863 | 0 | 0.026783 |
| GO_MICROTUBULE_CYTOSKELETON_ORGANIZATION | 320 | -1.70752 | 0 | 0.026931 |
| GO_NUCLEAR_TRANSCRIBED_MRNA_CATABOLIC_PROCESS_DEADENYLATION_DEPENDENT_DECAY | 54 | -1.70504 | 0.006834 | 0.027539 |
| GO_BLASTOCYST_DEVELOPMENT | 55 | -1.70343 | 0.004474 | 0.027802 |
| GO_NCRNA_TRANSCRIPTION | 79 | -1.70291 | 0 | 0.027762 |
| GO_CHROMOSOME_SEPARATION | 17 | -1.70038 | 0.010142 | 0.028356 |
| GO_NEGATIVE_REGULATION_OF_PROTEIN_CATABOLIC_PROCESS | 102 | -1.69423 | 0.002439 | 0.029987 |
| GO_PEPTIDYL_LYSINE_MODIFICATION | 288 | -1.6863 | 0 | 0.032224 |
| GO_MITOTIC_G2_M_TRANSITION_CHECKPOINT | 18 | -1.68563 | 0.012146 | 0.032257 |
| GO_CELLULAR_PROTEIN_COMPLEX_LOCALIZATION | 22 | -1.6854 | 0.004098 | 0.03212 |
| GO_REGULATION_OF_CENTROSOME_DUPLICATION | 29 | -1.68299 | 0.004149 | 0.032738 |
| GO_HOMOLOGOUS_CHROMOSOME_SEGREGATION | 43 | -1.68055 | 0.008811 | 0.033376 |
| GO_ONE_CARBON_METABOLIC_PROCESS | 34 | -1.68 | 0.006316 | 0.03333 |
| GO_REGULATION_OF_TELOMERE_MAINTENANCE | 60 | -1.67944 | 0.002123 | 0.033276 |
| GO_POSITIVE_REGULATION_OF_CELL_CYCLE_ARREST | 82 | -1.67892 | 0 | 0.033267 |
| GO_RETROGRADE_VESICLE_MEDIATED_TRANSPORT_GOLGI_TO_ER | 72 | -1.67662 | 0.002165 | 0.033914 |
| GO_HISTONE_H4_ACETYLATION | 41 | -1.67623 | 0.004301 | 0.033843 |
| GO_REGULATION_OF_CELL_CYCLE_ARREST | 103 | -1.67125 | 0.002421 | 0.035396 |
| GO_THIOESTER_BIOSYNTHETIC_PROCESS | 47 | -1.67118 | 0.00216 | 0.03524 |
| GO_NUCLEOBASE_CONTAINING_SMALL_MOLECULE_INTERCONVERSION | 19 | -1.66981 | 0.016736 | 0.035507 |
| GO_REGULATION_OF_SISTER_CHROMATID_COHESION | 17 | -1.66946 | 0.012739 | 0.0354 |
| GO_RRNA_TRANSCRIPTION | 16 | -1.66568 | 0.014257 | 0.036444 |
| GO_INTRACELLULAR_ESTROGEN_RECEPTOR_SIGNALING_PATHWAY | 17 | -1.65451 | 0.017822 | 0.040326 |
| GO_BINDING_OF_SPERM_TO_ZONA_PELLUCIDA | 30 | -1.65217 | 0.006211 | 0.041054 |
| GO_MICROTUBULE_BASED_PROCESS | 480 | -1.65161 | 0 | 0.040984 |
| GO_REGULATION_OF_CELL_CYCLE_CHECKPOINT | 26 | -1.64746 | 0.010417 | 0.042289 |
| GO_ERROR_PRONE_TRANSLESION_SYNTHESIS | 19 | -1.64428 | 0.016097 | 0.04334 |
| GO_REGULATION_OF_KERATINOCYTE_DIFFERENTIATION | 26 | -1.64375 | 0.008639 | 0.043297 |
| GO_SNRNA_PROCESSING | 21 | -1.64308 | 0.010081 | 0.043375 |
| GO_TRNA_METHYLATION | 20 | -1.64079 | 0.022945 | 0.04412 |
| GO_REGULATION_OF_MICROTUBULE_POLYMERIZATION_OR_DEPOLYMERIZATION | 164 | -1.63984 | 0.002747 | 0.044271 |
| GO_POSITIVE_REGULATION_OF_GENE_EXPRESSION_EPIGENETIC | 63 | -1.63853 | 0.002151 | 0.04463 |
| GO_POST_TRANSLATIONAL_PROTEIN_MODIFICATION | 34 | -1.63327 | 0.012121 | 0.046516 |
| GO_MATURATION_OF_SSU_RRNA_FROM_TRICISTRONIC_RRNA_TRANSCRIPT_SSU_RRNA_5_8S_RRNA_LSU_RRNA_ | 32 | -1.63303 | 0.010482 | 0.046398 |
